# Supplementary material for: Implementation of national antenatal hypertension guidelines: a multicentre multiple methods study
Source: BMJ Open. 2020 Oct 23;10(10):e035762. doi: 10.1136/bmjopen-2019-035762 (PMC7590365; doi:10.1136/bmjopen-2019-035762)
Supplement: Supplementary data [file bmjopen-2019-035762supp001.pdf]

Supplementary file 1

Chronic hypertension in pregnancy – healthcare professional survey

**Respondents**	**Number (97)**	**Percentage %**
Obstetrician<br>Of which are consultants	69<br>53	71.1<br>55
Midwife<br>Of which are specialist/ senior midwife	28<br>22	28.9<br>22.7
NHS hospital trusts represented<br>(including England, Northern Ireland, Scotland and Wales)	69	-

Question 1: If you see a pregnant woman with chronic hypertension who is currently taking either ACEIs or ARBs (e.g. at the beginning of pregnancy), how often would you ask her to stop taking them?

**Response**	**Number (97)**	**Percentage (%)**
Always	57	57.8
Almost always	27	27.8
About two thirds of the time	1	1
About half of the time	4	4.1
About a third of the time	0	0
Very rarely	1	1
Never	3	3.1
Missing	4	4.1

Question 2: What do you usually use as your first line anti-hypertensive treatment(s) for women with chronic hypertension in pregnancy?

**Anti-hypertensive (non-exclusive)**	**Number (97)**	**Percentage (%)**
Labetalol	85	87.6
Methyldopa	29	29.9
Nifedipine	32	33.0
Amlodipine	2	2.1

Question 3: What additional anti-hypertensive medication do you use for treating women with chronic hypertension in pregnancy?

**Anti-hypertensive (non-exclusive)**	**Number (97)**	**Percentage (%)**
Amlodipine	37	38.1
Atenolol	2	2.1
Doxazosin	23	23.7
Enalapril	1	1.0
Hydralazine (oral)	2	2.1
Labetalol	38	39.2
Methyldopa	60	61.9
Metoprolol	1	1.0
Nifedipine	79	81.4

Question 4: How frequently do you set a blood pressure target for women with chronic hypertension in pregnancy who need anti-hypertensive treatment (assuming no other co-morbidity) (mmHg)?

**Answer**	**Number (97)**	**Percentage %**
Always	36	37.1
Almost always	36	37.1
About two thirds of the time	8	8.2
About half of the time	3	3.1
About a third of the time	4	4.1
Very rarely	3	3.1
Never	1	1.0
Other<br>In the guidelines but compliance unknown<br>Frequency not described	6<br>2<br>4	6.2

Question 5: What blood pressure target do you usually set for pregnant women with chronic hypertension (assuming no other co-morbidity) (mmHg)?

**Systolic**	**Number (97)**	**Percentage %**	**Median (IQR1-IQR3)**
120	2	2.1	
125	0	0.0	
130	6	6.2	
135	2	2.1	
140	33	34.0	
145	0	0.0	
150	40	41.2	
155	1	1.0	
160	8	8.2	
Missing	4	4.1	
Median			150 (140-150)

**Diastolic**	**Number (97)**	**Percentage %**	**Median (IQR1-IQR3)**
80	9	9.3	
85	7	7.2	
90	37	38.1	
95	8	8.2	
100	27	27.8	
110	3	3.1	
Missing	5	5.2	
Median			90 (90-100)

Question 6: How often do you prescribe Aspirin for women with chronic hypertension in pregnancy?

**Answer**	**Number (97)**	**Percentage %**
Always	53	54.6
Almost always	36	37.1
About two thirds of the time	5	5.2
About half of the time	2	2.1
Very rarely	1	1.0

Question 7: At what gestation do these women usually receive their first Aspirin prescription?

**Answer**	**Number (97)**	**Percentage %**
Before 12 weeks	41	42.3
12-15+6 weeks	52	53.6
16-19+6 weeks	1	1.0
Missing answer	3	3.1

Question 8: For a woman with uncomplicated chronic hypertension in pregnancy (i.e. no additional risk factors), how many routine fetal growth scans do they receive (excluding nuchal and anomaly scans)?

**Additional scans**	**Number (97)**	**Percentage %**	**Median (IQR1-IQR3)**
None	4	4.1	
1	12	12.4	
2	23	23.7	
3	37	38.1	
4	21	21.6	
>4	1	1.0	
			3 (2-3)

Question 9: When do you usually plan birth for women with chronic hypertension whose blood pressure is controlled below 160/110?

**Gestation**	**Number (97)**	**Percentage (%)**	**Median (IQR1-IQR3)**
Before 34 weeks	3	3.1	
34-34+6 weeks	2	2.1	
35-35+6 weeks	2	2.1	
36-36+6 weeks	4	4.1	
37-37+6 weeks	27	27.8	
38-38+6 weeks	36	37.1	
39-39+6 weeks	41	42.3	
40-41 weeks	28	28.9	
Await spontaneous labour	5	5.2	
Other – individualised	4	4.2	
			38.5<br>(37-39)

Question 10: Involving pregnant women who have chronic hypertension in their pregnancy and birth planning is an important part of the consultation?

Sentiment	Number (97)	Percentage (%)
Agree Strongly	79	81.4
Agree	15	15.5
Slightly Agree	2	2.1
Slightly disagree	0	0.0
Disagree	0	0.0
Disagree Strongly	1	1.0

Question 11: If you wish, can you give an example of how you enable women to be actively involved in their care?

Themes	Number (47)	Percentage %
Total responses	47	
SDM in the following areas		
• Home BP	10	21
• Monitoring BP	6	12.8
• Anti-hypertensives	5	10.6
• Planning birth (induction of labour)	17	36
• Organisation of care	4	8.5
Discussing risks and benefits	2	4.3
How to identify pre-eclampsia	2	4.3

Question 12: In your maternity unit what term/s best describes the antenatal care provided to most women with chronic hypertension?

Care provision	Number (97)	Percentage %
Named consultant-led general antenatal clinic<br>(maternal medicine clinic)	63<br>(7)	64.9<br>(7.2)
Consultant-led specialist hypertension in pregnancy clinic	25	25.8
Multi-disciplinary clinic with additional medical professional	20	20.6
Consultant obstetrician and midwife antenatal clinic	15	15.5
Shared-care GP/ obstetrician/ midwife	7	7.2
Specialist midwifery care (e.g. medical conditions team)	6	6.2
Hospital midwifery care	1	1.0
Community based midwifery care	4	4.1
Day assessment unit	2	2.1

Question 13: In your maternity unit when do the pregnant women with chronic hypertension usually first get seen by an obstetrician?

Gestation	Number (97)	Percentage %
Before 12 weeks	24	24.7
12-15+6 weeks	63	64.9
16-27+6 weeks	9	9.3
Missing data	1	1.0

Question 14: Do you or someone in your unit specifically collect and analyse the outcomes of women with chronic hypertension in pregnancy annually?

Response	Number (97)	Percentage (%)
Yes	24	24.7
No	67	69.0
Unsure	4	4.1
Some aspects	2	2.0
