# Supplementary material for: Implementation of national antenatal hypertension guidelines: a multicentre multiple methods study
Source: BMJ Open. 2020 Oct 23;10(10):e035762. doi: 10.1136/bmjopen-2019-035762 (PMC7590365; doi:10.1136/bmjopen-2019-035762)
Supplement: Supplementary data [file bmjopen-2019-035762supp002.pdf]

Supplementary file 2

Interview topic guide for clinicians:

- Descriptions of the general approach to practice and how clinicians approach treatment decisions
- Discussion about the sources of evidence and knowledge that influence practice in general
- Participants' beliefs and experiences of using or having contact with clinical guidance (NICE in particular),
- Participants' views regarding how EBM and clinical guidelines could be better mobilised into practice

Interview schedule:

- Introductions
- Confidentiality
- I am interviewing you today for the CHAMPION study about chronic hypertension, you provide antenatal care for women with CHP is that right?
- Can you tell me a about your CHP clinic and your clinical practice in relation to chronic hypertension in pregnancy?
- How do you approach decision-making, for example commencing or changing hypertensive medication or delivery the baby early?
- What are you views and experiences of involving women in decision about their care or treatment plan?
- How do you source evidence and develop knowledge around hypertension in pregnancy?
- What are you experiences of clinical guidance e.g. NICE/ RCOG?
- How do you think we could better implement evidence-based medicine into clinical practice?

Reference: Grove, A., Clarke, A. and Currie, G. (2015) 'The barriers and facilitators to the implementation of clinical guidance in elective orthopaedic surgery: a qualitative study protocol', Implementation Science, 10(1), 81.

Women's experience of their care

- Introductions
- Confidentiality
- During this pregnancy you have been treated for chronic hypertension is that right?
- Can you tell me a bit about your high blood pressure and your pregnancy?
- How satisfied are you with the results of your care during your pregnancy?
- Thinking about your care during your pregnancy…Were you given information about your choices for maternity care?
- Were you given enough information to help you decide about your care?
- Were you given information at the right time to help you decide about your care?
- Did you have confidence and trust in the staff caring for you?

Reference: International Consortium for Health Outcomes Measurement. Pregnancy and Childbirth Standard Set and Reference Guide. 2016. http://www.ichom.org/medical-conditions/pregnancy-and-childbirth/.
