# Supplementary material for: Implementation of national antenatal hypertension guidelines: a multicentre multiple methods study
Source: BMJ Open. 2020 Oct 23;10(10):e035762. doi: 10.1136/bmjopen-2019-035762 (PMC7590365; doi:10.1136/bmjopen-2019-035762)
Supplement: Supplementary data [file bmjopen-2019-035762supp003.pdf]

Supplementary file 3

Maternal demographics of women observed, interviewed and included for case-note review. Women interviewed are a subset of those observed. Case-notes identified for review are a different cohort of women.

Women demographics	Observed n=28 (%)	Interviewed n=18 (%)	Case-notes n=55 (%)
**Ethnicity**			
White British	9 (32.0)	7 (39.0)	15 (27.3)
White Other	6 (21.0)	4 (22.0)	8 (14.5)
Black	9 (32.0)	5 (28.0)	18 (32.7)
Asian	2 (7.0)	1 (5.5)	8 (14.5)
Any other	2 (7.0)	1 (5.5)	6 (10.9)
**Parity at booking**			
0	9 (32.0)	7 (39.0)	15 (27.3)
1	11(39.0)	7 (39.0)	21 (38.2)
2	7 (25.0)	4 (22.0)	10 (18.2)
3	0 (0.0)	0 (0.0)	6 (10.9)
4	0 (0.0)	0 (0.0)	2 (3.6)
5	1 (4.0)	0 (0.0)	1 (1.8)
**Age**			
20-34	17 (61.0)	11 (61.0)	23 (41.8)
35-39	7 (25.0)	5 (28.0)	21 (38.9)
40-44	4 (14.0)	2 (11.0)	11 (20.4)
**BMI**			
<18.5	0 (0.0)	0 (0.0)	1/52 (1.9)
18.5-24.9	7 (25)	6 (33.3)	13/52 (25.0)
25-29.9	10 (36)	6 (33.3)	13/52 (25.0)
30-34.9	9 (32)	5 (28.0)	11/52 (21.2)
35-39.0	2 (7)	1 (5.5)	6/52 (11.5)
>40.0	0 (0)	0 (0.0)	8/52 (7.7)
