# Supplementary material for: Implementation of national antenatal hypertension guidelines: a multicentre multiple methods study
Source: BMJ Open. 2020 Oct 23;10(10):e035762. doi: 10.1136/bmjopen-2019-035762 (PMC7590365; doi:10.1136/bmjopen-2019-035762)
Supplement: Supplementary data [file bmjopen-2019-035762supp004.pdf]

Supplementary file 4

Pregnancy and birth outcomes – Case notes review

**Outcomes**	**Case notes review**<br>**Nominator/denominator (%)**
Women with episode of severe hypertension	25/55 (45.5)
1st trimester episode	2/40 (5.0)
2nd trimester episode	13/40 (32.5)
3rd trimester episode	25/40 (62.5)
Birth weight - median<br>(IQR1 – IQR3)	2927.5<br>(2592.5 - 3200)
Admission to NNU	9/55 (16.4)
