# Supplementary material for: Implementation of national antenatal hypertension guidelines: a multicentre multiple methods study
Source: BMJ Open. 2020 Oct 23;10(10):e035762. doi: 10.1136/bmjopen-2019-035762 (PMC7590365; doi:10.1136/bmjopen-2019-035762)
Supplement: Supplementary data [file bmjopen-2019-035762supp005.pdf]

Supplementary file 5

Target blood pressure setting and prescribing practices per Trust – as derived from case-note review

	**Hospital Trust 1 n=29 (%)**	**Hospital Trust 2 n=13 (%)**	**Hospital Trust 3 n=13 (%)**
**Target BP documented <150/100mmHg**	20/26 (77.0)	3/13 (23.0)	5 (38.0)
**Labetalol**	12/26 (46.0)	7/12 (58.3)	9/11 (82.0)
**Nifedipine**	9/26 (34.5)	0/12 (0.0)	0/11 (0.0)
**Methyldopa**	3/26 (11.5)	4/12 (33.3)	1/11 (9.0)
**Other**	2/26 (8.0)	1/12 (8.3)	1/11 (9.0)
