# Supplementary material for: Implementation of national antenatal hypertension guidelines: a multicentre multiple methods study
Source: BMJ Open. 2020 Oct 23;10(10):e035762. doi: 10.1136/bmjopen-2019-035762 (PMC7590365; doi:10.1136/bmjopen-2019-035762)
Supplement: Author's manuscript [file bmjopen-2019-035762.draft_revisions.pdf]

BMJ Open is committed to open peer review. As part of this commitment we make the peer review history of every article we publish publicly available.

When an article is published we post the peer reviewers' comments and the authors' responses online. We also post the versions of the paper that were used during peer review. These are the versions that the peer review comments apply to.

The versions of the paper that follow are the versions that were submitted during the peer review process. They are not the versions of record or the final published versions. They should not be cited or distributed as the published version of this manuscript.

BMJ Open is an open access journal and the full, final, typeset and author-corrected version of record of the manuscript is available on our site with no access controls, subscription charges or pay-per-view fees (http://bmjopen.bmj.com).

If you have any questions on BMJ Open's open peer review process please email info.bmjopen@bmj.com

BMJ Open

# Implementation of national hypertension in pregnancy guidelines: a multi-centre mixed methods study



| | |
|---|---|
| Journal: | *BMJ Open* |
| Manuscript ID | bmjopen-2019-035762 |
| Article Type: | Original research |
| Date Submitted by the Author: | 19-Nov-2019 |





| | |
|---|---|
| Complete List of Authors: | Whybrow, rebecca; King's College London, Women and Children's Health; Guy's and Saint Thomas' Hospitals NHS Trust, Division of Women and Children's Health<br>Webster, Louise; King's College London, Women and Children's Health<br>Girling, Joanna; Chelsea and Westminster Hospital NHS Foundation Trust<br>Brown, Heather; Brighton and Sussex University Hospitals NHS Trust<br>Wilson, Hannah; King’s College London, Women and Children's Health<br>Sandall, Jane; Kings College, London, Women and Children's Health<br>Chappell, Dr Lucy; King’s College London, Department of Women and Children’s Health; Guy's and Saint Thomas' Hospitals NHS Trust, Division of Women and Children's Health |





| | |
|---|---|
| Keywords: | Maternal medicine < OBSTETRICS, OBSTETRICS, Hypertension < CARDIOLOGY |
| | |



SCHOLARONE™
Manuscripts

*I, the Submitting Author has the right to grant and does grant on behalf of all authors of the Work (as defined in the below author licence), an exclusive licence and/or a non-exclusive licence for contributions from authors who are: i) UK Crown employees; ii) where BMJ has agreed a CC-BY licence shall apply, and/or iii) in accordance with the terms applicable for US Federal Government officers or employees acting as part of their official duties; on a worldwide, perpetual, irrevocable, royalty-free basis to BMJ Publishing Group Ltd ("BMJ") its licensees and where the relevant Journal is co-owned by BMJ to the co-owners of the Journal, to publish the Work in this journal and any other BMJ products and to exploit all rights, as set out in our licence.*

*The Submitting Author accepts and understands that any supply made under these terms is made by BMJ to the Submitting Author unless you are acting as an employee on behalf of your employer or a postgraduate student of an affiliated institution which is paying any applicable article publishing charge ("APC") for Open Access articles. Where the Submitting Author wishes to make the Work available on an Open Access basis (and intends to pay the relevant APC), the terms of reuse of such Open Access shall be governed by a Creative Commons licence – details of these licences and which Creative Commons licence will apply to this Work are set out in our licence referred to above.*

*Other than as permitted in any relevant BMJ Author's Self Archiving Policies, I confirm this Work has not been accepted for publication elsewhere, is not being considered for publication elsewhere and does not duplicate material already published. I confirm all authors consent to publication of this Work and authorise the granting of this licence.*

For peer review only

**Title:** Implementation of national hypertension in pregnancy guidelines: a multi-centre mixed methods study

Rebecca Whybrow[1], Louise Webster[1], Joanna Girling[2], Heather Brown[3], Hannah Wilson[1], Jane Sandall[1], Lucy Chappell[1]

1 Department of Women and Children's Health, King's College London, St Thomas' Hospital, London, UK.

2 Chelsea and Westminster Hospital NHS Foundation Trust, London, UK.

3 Brighton and Sussex Universities Hospital Trust, East Sussex, UK.

Corresponding author: Rebecca Whybrow, Department of Women and Children's Health, King's College London, St Thomas' Hospital, London, UK. Rebecca.whybrow@kcl.ac.uk Tel: 07804690276

**Word Count:** 4225

**Abstract:**

**Objective** To evaluate the implementation of NICE hypertension in pregnancy guidelines, to identify strategies to reduce incidences of severe hypertension and associated maternal and perinatal morbidity and mortality in pregnant women with chronic hypertension.

**Methods** We used a multi-method multi-site approach to establish implementation of guidelines and the associated barriers and facilitators. We used a national survey (n=97), case-notes review (n=55) and structured observations (n=42) to assess implementation. The barriers and facilitators to implementation were identified from semi-structured qualitative interviews with professionals (n=13) and pregnant women (n=18) using inductive thematic analysis. The findings were integrated and evaluated using the Consolidated Framework for Implementation Research (CFIR).

**Setting and participants** Pregnant women with chronic hypertension and their principle carers (obstetricians, midwives and physicians), at three NHS hospital trusts with different models of care.

**Results** We found severe hypertension to be prevalent (46% of case notes reviewed) and target blood pressure practices to be sub-optimal (56% of women had an antenatal blood pressure target documented). Women were infrequently given information (52%) or offered choice (19%) regarding antihypertensives. Women (14/18) reported conflict in taking antihypertensives and non-adherence was prevalent (8/18). Women who were concordant with treatment recommendations described having mutual trust with professionals mediated through appropriate information, side-effect management and involvement in decision-making. Professionals reported needing updates and tools



For peer review only - http://bmjopen.bmj.com/site/about/guidelines.xhtml

for target blood pressure setting and shared decision-making underpinned by antihypertensive safety and effectiveness research.

**Conclusions** Women's nonadherence to antihypertensives is higher than anticipated, which is likely to be contributing to adverse perinatal outcomes, as is sub-optimal target setting practices. Education and decision-making strategies are needed to address both clinician and women's behaviour. Further research into the effectiveness and long-term safety of common antihypertensives is also required.

**Strengths and limitations of this study**

- Multi-methodological approaches and an implementation framework improved the reliability, validity and generalisability of the study.
- Structured observations were carried out using a validated tool with high interrater reliability.
- Women's medication behaviours were explored in-depth using a novel qualitative interview approach and have identified antihypertensive side-effects to be a determinant of non-adherence in pregnant women.
- The study is limited by the population size (and hence statistical power) for each of the methods.
- Respondents to the survey were self-selecting and may represent a relatively interested group of healthcare professionals.



For peer review only - http://bmjopen.bmj.com/site/about/guidelines.xhtml

**BACKGROUND**

Hypertension in pregnancy is one of the leading causes of maternal mortality worldwide[1] and although mortality is declining in the UK,[2] women can still experience substantial morbidity from complications such as eclampsia and stroke[3]. Additionally, perinatal mortality remains high, with the UK population-attributable risk of stillbirth from chronic hypertension at 14%[4] and around half of all neonates born to mothers who have had severe hypertension in pregnancy being admitted to the neonatal unit[5]. The morbidity and mortality attributable to hypertension, in many cases, may be modifiable through optimal use of antihypertensive agents during pregnancy.

The National Institute for Health and Care Excellence (NICE) hypertension in pregnancy guidelines (2010)[6] and linked quality statements (2013)[7] contain a quality statement regarding the provision of information on the use of safe antihypertensive medication in pregnancy and has related guidance that recommends discontinuation of teratogenic medications such as angiotensin-converting-enzyme inhibitors or angiotensin II receptor blockers with prescribing of safe alternatives. Any prescribing of alternative antihypertensive medication should be dependent on pre-pregnancy treatment, side-effect profiles and teratogenicity. A second quality statement advocates that women taking antihypertensive medication should have a blood pressure target (usually of less than 150/100mmHg) set in pregnancy. All NICE guidelines are underpinned by the recommendation of enabling patients to actively participate in their care which includes adopting a shared decision-making approach to treatment decisions[8].

Despite publication of the guideline almost a decade ago, the implementation and evaluation of associated determinants of uptake have not been nationally evaluated. As a result, targeted strategies to reduce maternal and perinatal morbidity (and mortality) resulting from severe hypertension remain unidentified. The study draws on the Consolidated Framework for Implementation Research (CFIR) as a guide for analysing, interpreting, and reporting implementation-related findings. Without a theoretical framework to guide data collection, analysis, and interpretation, implementation researchers are less able to generalise the findings beyond the context in which the data were collected. [9] The aim of this study was to evaluate the variance in provision of, and the barriers and facilitators involved in, the delivery of the national guidelines for the management of hypertensive disorders of pregnancy, using this framework.



For peer review only - http://bmjopen.bmj.com/site/about/guidelines.xhtml

## RESEARCH DESIGN AND METHODS

### Study setting and overall methodology

The CHAMPION study (Chronic Hypertension in pregnAncy iMPlementatION study) is a multi-methods evaluation of the implementation of the NICE hypertension in pregnancy guidelines (2010 and updated in 2013).[6 7] Ethical approval for the CHAMPION study was provided by the National Research Ethics Service (17/LO/2041). The study aimed to evaluate the variance in implementation of hypertension management practices set out in the NICE hypertension in pregnancy guidelines (2010).[6] As all guidelines should be underpinned by the 'Patient experience in adult NHS services guideline'[8] which specifies 'actively involving patient in decisions about their care through 'information provision' and 'shared decision-making' the provision of information and women's involvement in decision-making was also evaluated. The involvement of women in decision-making was considered integral to the implementation study because successful hypertension management strategies involve the adherence to, alongside the prescribing of, antihypertensive medication.

Implementation was assessed through multiple methods: an online national survey of healthcare professionals, designed to describe general trends in guideline implementation; through case-notes review, a method that assessed the documentation of hypertension management occurrence in each woman's maternity record. Aspects of care that would not normally be documented or are more difficult to capture, such as in-consultation discussions and occurrence of shared decision-making were assessed through observations carried out by a midwife researcher (RW). The evaluation of the barriers and facilitators to implementation of NICE guidelines was assessed through qualitative interviews (with women and healthcare professionals) using the Consolidated Framework for Implementation Research (CFIR). The CFIR framework specifically evaluates five key domains that influence implementation; each domain has several subgroups to it, although only those relevant to this study have been identified. These include the intervention characteristics (the NICE guidelines), the outer context (the pregnant women), the inner context (NHS maternity services), individual context (the healthcare professionals) and the process of implementation (potential strategies).

Implementation of guidelines was assessed between November 2017 to December 2018 at three NHS Trusts with typical configurations of services for pregnant women with hypertension in the UK. Hospital Trust 1 was a tertiary city centre hospital with a newly formed specialist service that included consultant obstetricians, obstetric physicians and midwives who provided antenatal and intrapartum care to women with chronic hypertension within a specialist clinic; Hospital Trust 2 was a suburban district general hospital with a consultant-led antenatal clinic with antenatal midwives alongside providing care to women with a variety of pre-existing medical conditions; and Hospital Trust 3 had

For peer review only



For peer review only - http://bmjopen.bmj.com/site/about/guidelines.xhtml

both a tertiary and a semi-rural hospital with a joint obstetric and physician led clinic and usual community-based midwifery care. The NICE hypertension in pregnancy guidelines (2010)[6] had been adopted into local clinical guidelines at all three participating NHS Trusts for several years prior to the assessment of implementation.

**The National Survey**

The implementation of evidence-based practices for the management of hypertension in pregnancy was assessed through self-reporting using an online survey (surveygizmo/s3). We embedded questions relating to the uptake of the NICE hypertension in pregnancy guidelines (2010)[6] using the TIDieR framework.[10] The 12-item TIDieR checklist (brief name, why, what (materials), what (procedure), who provided, how, where, when and how much, tailoring, modifications, how well (planned), how well (actual)) is an extension of the CONSORT 2010 statement (item 5) and the SPIRIT 2013 statement (item 11). Although the emphasis of the checklist is on trials, the guidance was adopted for this study as it is also intended to apply across all evaluative study designs.[10] National organisations including British Maternal and Fetal Medicine Society (BMFMS), Macdonald UK Obstetric Medicine Society (MOMS) and Royal College of Midwives (RCM) distributed the survey (April to September 2018). A convenience sample of ninety-seven healthcare professionals from sixty-nine NHS Trusts was obtained, including 53 consultant obstetricians (55%), 16 doctors in training (16%), 22 specialist midwives (23%) and six community midwives (6%) (full copy of survey questions shown in supplementary material 1).

**Case-notes review**

The implementation of NICE guidelines (2010)[6] was also assessed through review of 100 maternity case-notes from the maternity electronic databases (32, 33, 35 per Trust). At Hospital Trust 1 this consisted of all women who had given birth over the last three months of 2017, at the other two Trusts this consisted of all women who had given birth in 2017 as they had approximately a quarter of the number of women with chronic hypertension per annuum compared to Hospital Trust 1. The inclusion criteria consisted of women with chronic hypertension in pregnancy (defined as hypertension present at the booking visit or before 20 weeks or already taking antihypertensive medication when referred to maternity services)[6] who had been booked for care at one of the participating NHS Trusts. Women who miscarried before the first obstetric appointment or who delivered at another hospital and or had incomplete health records were excluded from the study. A total of 55 sets of notes (29, 13, 13 per Trust) met the inclusion and exclusion criteria. Data extraction based on the NICE hypertension in



For peer review only - http://bmjopen.bmj.com/site/about/guidelines.xhtml

pregnancy guidelines (2010)[6] was completed by two midwife researchers (RW, HW), and discrepancies were resolved by discussion between the two researchers. Unclear or absent documentation was recorded as missing data.

**Observations**

Forty-two antenatal appointments involving 23 women with chronic hypertension and their respective doctors (nine) and midwives (five) were observed by a midwife researcher (RW) at the three NHS Trusts. Staff and women gave written informed consent. During observations, data about antenatal care provision were recorded using the Calgary-Cambridge communication guide[11] chosen for its high interrater reliability. For example, offering choice is a sub-section of shared decision-making and is defined as "encourages patient to make choices and decisions to the level that they wish". Attainment of each section and sub-sections was established through the analysis of all 42 appointments using descriptive statistics.

**Semi-structured interviews**

Views about barriers and facilitators to implementation of evidence-based guidelines were collected from nine doctors and four midwives who were providing antenatal care for women with chronic hypertension. The interviews were carried out by a midwife researcher (RW) following informed consent and took place in privacy away from the clinical setting. The interviews were audio transcribed, coded and thematically analysed using inductive reasoning. The codes generated formed small themes which were organised into the CFIR evaluation guide [12]. As formal implementation strategies had not been adopted beyond producing local guidance, interviewees were asked how they thought they could improve the implementation in the future.

Semi-structured interviews with 18 women recruited for antenatal observations were carried out in the third trimester with informed consent. Women were asked about their antenatal care experiences using an interview schedule which reflected the concepts from the International Consortium for Health Outcome Measure (ICHOM) maternity standards sets[13] which include women's overall satisfaction with their care during pregnancy; satisfaction with information provision and their relationships with their care providers. ICHOM standards are internationally recognised measures that evaluate health outcomes that are important to patients (or pregnant women) and are used to improve local healthcare and compare outcomes internationally. The closed survey questions were turned into open ended questions to explore in-depth the quality of antenatal care provided. The interviews were carried out by a midwife researcher (RW) and took place away from the clinical setting, with assurance that discussions would not be shared with healthcare professionals and that

For peer review only



For peer review only - http://bmjopen.bmj.com/site/about/guidelines.xhtml

participation or non-participation would not influence their care. The interviews were audio transcribed, coded and thematically analysed using an inductive approach. Women's experiences were analysed to improve understanding of their antenatal care needs, which included how their hypertension was managed and the barriers and facilitators to the uptake of antihypertensives in pregnancy.

**Data analysis**

The quantitative and qualitative data were initially analysed separately to generate independent results. Descriptive analysis and summary statistics were used for the quantitative data. The semi-structured interviews were thematically analysed using inductive techniques.[14] The mixed-methods data were integrated and analysed using the CFIR evaluation framework.[12] The interpretation of the intervention constructs (characteristics the inner and outer settings, the individual characteristics and the implementation processes) was carried out initially by the midwife researcher (RW) who collected the data, then with a second and third researcher (LC, JS) interpreting and agreeing final interpretation of integrated data. Rigour was maintained through member reflection, attention to interview and transcription quality and systematic analysis. Rigour was improved using multiple data sources and analytic integration methods.

**Patient and Public Involvement**

A patient participant involvement (PPI) group consisting of women with experience of hypertension in pregnancy and a maternity voices partnership group provided feedback on the design of the study, research questions and outcome measures. Women in both groups wanted measures of information provision and shared decision-making to be included in the evaluation of the national guidelines implementation as both underpinned the prescribing practices being measured. The advisory groups reviewed and approved the patient information leaflet and recommended women participants be asked to provide their email addresses if they would like to receive a copy of the study results.

**RESULTS**

Antenatal care for women with chronic hypertension was provided by consultant obstetricians and midwives at all three hospitals. In two of the hospitals, women with chronic hypertension had designated midwives attached to the obstetric clinic. Approximately one-third of those recruited to the study had a BMI over 30kg/m$^2$, approximately one-third were over the age of 35 and approximately one-third were of black minority ethnic backgrounds (shown in supplementary material 2). Hospital Trust 1 had four times the population of women with chronic hypertension compared to



For peer review only - http://bmjopen.bmj.com/site/about/guidelines.xhtml

the other two units, comprising a large black minority ethnic population (many with associated co-morbidities). Perinatal outcomes from the fifty-five pregnancies showed that just under half of the women (46%) developed severe hypertension and that one in six babies were admitted to the neonatal unit (16%) (shown in supplementary material 3). At all three hospitals electronic coding of women with chronic hypertension was inaccurate and episodes of severe hypertension were not electronically recorded.

**Implementation of NICE hypertension in pregnancy 2010 guidelines and 2013 quality standards**

Setting a blood pressure target (quality statement 3)

Both the survey and the case-notes review found the practice of setting an antenatal target blood pressure to be variable (table 1). Just over half of women with chronic hypertension had a target blood pressure documented in maternity notes (44% did not) yet substantial variation in practice between hospitals existed. At Hospital Trust 1, 77% of women had a target blood pressure documented in pregnancy compared to 23% and 38% at Hospital Trusts 2 and 3 respectively (supplementary material 4). The survey results support these findings as only a third of healthcare professional respondents reported always setting a target. The practice of undocumented 'unshared' target setting was identified through case-notes review, as for about three quarters of women whose blood pressure rose above systolic 150mmHg and or diastolic 100mmHg action was taken by professionals to lower it (24% did not) (table 1).

Table 1. Variation in implementation of evidence-based care evaluated through a national survey of obstetricians and midwives and women's case-notes review at three representative NHS Trusts.

| **Care quality indicators** | **National Survey n=97 (%)** | **Case-notes review n=55 (%)** |
|---|---|---|
| **Blood pressure target setting (QS3)** | | |
| Target blood pressure 'always' set | 36 (37.1) | |
| Target blood pressure 'almost always' set | 36 (37.1) | |
| Target blood pressure 'never' set/ not documented | 1 (1.0) | 26 (43.6) |
| Target blood pressure set at first opportunity (whichever first: booking or commencement of AHT) | - | 9 (18.0) |
| **Systolic target blood pressure** | | |
| <160mmHg | 8 (8.2) | |
| <150mmHg | 89 (91.8) | 2 (7.4) |



For peer review only - http://bmjopen.bmj.com/site/about/guidelines.xhtml

| | | |
|---|---|---|
| ≤140mmHg | | 27 (49.0) |
| **Diastolic target blood pressure** | | |
| <100mmHg | 94 (96.9) | 2 (7.4) |
| ≤90mmHg | | 27 (49.0) |
| Action taken to reduce blood pressure if above 150/100mmHg | | 13/17 (76.5) |
| **Safe antihypertensive prescribing (linked to QS1)** | | |
| **ACEi and ARBs cessation** | | |
| On ACEis or ARBs at antenatal booking appointment | | 4 (7.3) |
| Stopping ACEi or ARBs at first app if woman on either | | |
| Always | 57/86 (66.3) | - |
| Almost always | 27/86 (31.4) | - |
| ACEis or ARBs stopped at 1st obstetric appointment | | 4/4 (100.0) |
| **1st line AHT prescribing (non-exclusive)** | | |
| Labetalol | 85 (87.6) | 28 (50.9) |
| Nifedipine | 32 (33.0) | 9 (16.4) |
| Methyldopa | 29 (29.9) | 8 (14.5) |
| Other e.g. amlodipine | 2 (2.1) | 4 (7.3) |
| None | - | 6 (10.9) |
| **2nd line AHT prescribing (non-exclusive)** | | |
| Nifedipine | 79 (81.4) | 9 (16.4) |
| Methyldopa | 60 (61.9) | 4 (7.3) |
| Labetalol | 38 (39.2) | 3 (5.4) |
| Amlodipine | 37 (38.1) | 2 (3.6) |
| Doxazosin | 23 (23.7) | 0 (0.0) |
| Other | 5 (5.2) | 0 (0.0) |
| None | - | 37 (I67.3) |

Antihypertensive information provision, decision-making and prescribing (quality statement 1 and associated guidance)

Variation in practice regarding first- and second-line prescribing was identified through both the notes review and survey (table 1). In both, labetalol was the most commonly prescribed first line and nifedipine the most commonly used second line antihypertensive agent; nevertheless, in about half



For peer review only

For peer review only - http://bmjopen.bmj.com/site/about/guidelines.xhtml

of the cases reviewed labetalol was not the first line antihypertensive prescribed. Variation in prescribing practice existed when comparing the different Hospital Trusts, possibly reflecting women's ethnicity (supplementary material 4), clinician preference or women indicating their medication preferences through shared decision-making.

Information provision about antihypertensive prescribing

Across all three Trusts, 52% (41/79) of the time the correct type and amount of information was provided during the consultation (measured using the Calgary-Cambridge Guide). Visual techniques such as drawing or using charts to provide information occurred during consultation in 14% (3/21) of cases.

Achieving a shared understanding: incorporating the patient's perspective

Of the survey respondents 96.9% strongly agreed or agreed that involving women with chronic hypertension in management plans during pregnancy was important. However, when asked to give examples of how they involve women, only 4.3% identified discussing risks and benefits of treatment choice and 10% of respondents identified that women could be involved in plans about antihypertensive prescribing. The observations in the three hospital trusts found that 43% of the time (41/96) shared decision-making occurred and 19% of women (3/16) were offered a choice regarding their hypertensive plans (including choice of antihypertensive).

**Barriers and Facilitators to implementation (CFIR)**

Intervention characteristics (evidence and guideline)

All professionals interviewed, except one, saw value in having national guidance and understood that the local guidelines had been adapted from the 2010 national guideline[6]. Midwives relied more on local guidelines compared to obstetricians who referred more commonly to NICE guidelines. Some of the medical professionals had been involved in the development of a NICE guideline and were aware of the strengths and limitations of producing evidence-based recommendations. Professionals described difficulties in creating guidelines where there is a paucity of robust data as is sometimes the case in maternity care. Weak, out of date or absent evidence influenced doctors' decisions to implement guidelines. Those working in hospital Trust 1 described the weaknesses in the evidence underpinning the hypertension guidelines and described relying more on recent research compared to older national guidelines (table 2). The professionals identified that further research is necessary to support evidenced-based national guidelines (figure 1).



For peer review only - http://bmjopen.bmj.com/site/about/guidelines.xhtml

Table 2. Barriers to healthcare professional's implementation of hypertension in pregnancy guidelines

| Barriers | Frequency | Items | Representative answer |
|---|---|---|---|
| **Intervention characteristics** | | | |
| Evidence strength, quality, source and adaptability | 17 | AHT prescribing; target setting; | - "I think the fact that it says use labetalol first line is not what we do, I don't believe the evidence for labetalol being better than methyldopa is there."[H]<br>- "we can't get away from the fact that there aren't the source data there to make evidence-based guidelines."[B]<br>- So, I kept a close track of what was happening with the CHIPS study…I got a lot of information and knowledge from it."[A] |
| **Inner setting** | | | |
| Structural characteristics | 43 | Information provision; pathways and models; training and education; time | - "I don't think we have a hand-out for, to give to hypertensive women about hypertension in pregnancy"[L]<br>- "we don't have a dedicated hypertension clinic here. So, most of these women will get seen in general antenatal clinic"[I]<br>- "you have people coming in three times weekly or something for their blood pressure, really? And other people who perhaps aren't being seen enough"[I] |
| Relative priority | 26 | Guidelines; self-study; beliefs; experience; | - "Well actually I don't even know what the NICE guidelines are for hypertension, I'm not a… as my colleagues will tell you, not a huge fan of NICE, in many ways."[L]<br>- "I'm not just interested in guidelines; I'm interested in people's clinical experience…and that feel."[C] |
| Culture of decision-making | 19 | Patriarchy; shared decision-making; type of decision: | - "Doctors… see it as patients not doing what they're told"[A]<br>- "I think that there's a balance to be had between involving women in the decisions, versus, them coming for expert recommendations"[F] |

For peer review only



For peer review only - http://bmjopen.bmj.com/site/about/guidelines.xhtml

| | | | |
|---|---|---|---|
| | | emergency, urgent and non-urgent | - “If I have a clinical situation where I want to start antihypertensives because she’s got a dangerously high blood pressure, then that discussion is inevitably truncated.” [B] |
| **Individual characteristic** | | | |
| Beliefs about the intervention | 35 | AHT medication; AHT safety and side-effects; target setting | - “National guidelines do not sanction any particular antihypertensive, or that the, the drug licenses do not sanction any particular antihypertensive”[B]<br>- “I think that might be something we’re not quite as good at as we should be about defining a target for women….I suspect it’s something we don’t really document and clarify”[H] |
| Self-efficacy | 17 | Women’s concordance/ desire for involvement/ first language | - “I think sometimes women don’t necessarily want to make the decision”[D]<br>- “There’s a lot of ‘mumsnet’….and I would say they take a, that advice just as seriously as they do the advice that we give them here.”[C] |
| **Process of implementation** | | | |
| Engaging people and process of implementation | 16 | Using guidelines; updates, toolkits and information; shared decision-making | - “Awareness for people, if you’re a busy jobbing healthcare practitioner, keeping up to date with each new area”[H]<br>- “Practical toolkits to help with that consultation”[B]<br>- Evidenced based information having it more readily available for patient”[D] |
| Opinion leaders; Champions; | 5 | Utilisation of opinion leaders/ champions in implementation | - “I find as a midwife sometimes you’re a bit powerless, you know what the guidelines are, but depending on the doctor you’re working with, tends to be the influencing factor on the decisions that are made… so it seems to be clinician-based guidelines sometimes, rather than the trust or national guidelines”[D][1] |

[1] Letters [A-M] represent the healthcare professionals interviewed



For peer review only - http://bmjopen.bmj.com/site/about/guidelines.xhtml

Inner setting (organisation structure and culture)

The most frequently cited barriers to implementing high quality care for women with chronic hypertension were linked to the structure and organisation of antenatal care. Interviewees reported that a lack of consensus and guidance exists relating to models of care (such as whether specialist services would improve outcomes through better implementation) and pathways of care (such as frequency of blood pressure and medication reviews) (table 2). Evidence-based recommendations on models, and pathways of care, were identified as future facilitators to providing optimal antenatal care (figure 1). Whilst most healthcare professionals initially described the uptake of the guidelines as a clinical priority during the interviews, clinicians identified difficulty with keeping up with recommendations and using them alongside clinical judgement as barriers to implementation (table 2).

Healthcare professionals considered the absence of written information a barrier to the uptake of antihypertensives in women with hypertension (table 2). A degree of paternalism exists in relation to involving women in decisions about their care. In principle, clinicians would like to involve women in decision-making, yet they gave many examples of situations where they would exercise restraint in doing so (table 2). Education and tools to support shared decision-making were identified as facilitators to optimizing antenatal care for women with hypertension (figure 1).

Characteristics of individuals (beliefs, knowledge and self-efficacy)

Interview analysis identified doctors' and midwives' knowledge and beliefs as the second most frequently cited barrier and facilitator to the implementation of hypertension management guidelines (table 2). There existed confusion about whether the guidelines sanction one antihypertensive medication over another for the management of chronic hypertension and if so, what evidence was used to support this. Likewise, confusion about blood pressure targets was described frequently as outcomes from a recent randomised controlled trial superseded the pre-dated national guidelines (table 2). Whilst midwives experienced less self-efficacy than the doctors, doctors still experienced difficulties in this area. They occasionally described the women's beliefs and views as a barrier to implementing the recommendations (table 2).

Outer setting (women's views and experiences)



For peer review only - http://bmjopen.bmj.com/site/about/guidelines.xhtml

300 The quality of antenatal care experience was affected by women's internal conflict. There was also a
301 high degree of variance in medication adherence and concordance. Analysis identified that women
302 require quality information about antihypertensives and their side-effects, blood pressure ranges in
303 pregnancy, as well as support to actively participate in decision-making.

304

305 *Conflict*

306 The majority (14 of 18) of women experienced internal conflict relating to the management of their
307 hypertension during pregnancy, defined as a state of uncertainty about the course of action to take
308 often in relation to making choices involving risk or uncertainty of outcomes (8) (figure 2a). The causes
309 of conflict were identified as a lack of information provision, poorly managed side-effects, women's
310 personal beliefs and factors relating to the healthcare professional (table 3).

311



For peer review only - http://bmjopen.bmj.com/site/about/guidelines.xhtml

Table 3. Barriers to women's uptake of hypertension in pregnancy guidelines

| Women's sources of conflict | Frequency | Items | Representative answer |
|---|---|---|---|
| Information | 30 | Medication (choices, dose, effectiveness, safety, interactions); severity of HTN; effect of HTN on pregnancy | - "[I wanted to know] how safe it is, about the dosage, about the, taking the med-, this medication, about the side-effects and so and so and so, if they think any other option for me, or if this medication is not working, what will be the other option for me"[J]<br>- "He was, you still need to carry on with your ramipril. I know I can't take it. It says in the leaflet not to take once you've hit 6 weeks, you need to stop. So, he was like oh, and then he phoned here, and he said oh well just take what you took before"[H] |
| Side-effects | 21 | Maternal side-effects; fetal side-effects; Interactions ; allergies; choices | - "They gave me first three, twice a day, then I was so giddy where I couldn't, if I take, I had to sleep all day for two days…Then I complained, but they still say to still take tablet."[I]<br>- "I'm on 18 pills a day, I do worry a bit about how they kind of potentially interact with each other and affect the baby"[F] |
| Beliefs | 17 | Hypertension status; understanding HTN; effectiveness AHT; safety AHT | - "I felt like I had to justify why I wasn't taking my tablet, which to me didn't seem right, 'cause if it, if my blood pressure was normal, and I took a tablet, surely my blood pressure then would be low?"[Q]<br>- "cause everything I take my baby takes. So, it's like, what happens if my child comes out and then they're addicted to something, or they're high-strung because of something, or they're really moody and they're crying all the time because of the medicine I've had to take for the past 4 months"[L] |



For peer review only - http://bmjopen.bmj.com/site/about/guidelines.xhtml

| | | | |
|---|---|---|---|
| | | | |
| HCP factors | 17 | Continuity; listening to women; explaining regimes, mutual trust; communication | - "My issue has been where I've seen somebody who doesn't know the history, and typically they are a more junior doctor, and typically they are ticking a box and following a flow chart….the doctor said, you know, we're going to come to an agreement together but there was absolutely no discussion, she had no interest in what I had to say."[K] |
| External factors | 7 | Family and friends; internet; access to services | - "My dad had been on beta blockers, which is what labetalol is, when he was younger, and he found, he was very ill on them, so he gave me a really negative impression of them"[P2] |

[2] Letters [A-R] represent the pregnant women interviewed



For peer review only - http://bmjopen.bmj.com/site/about/guidelines.xhtml

*Concordance*

All women identified as concordant with healthcare professional management plans described being adherent to their antihypertensives. Facilitators to concordance included trust in the healthcare professional, mediated through information about safety of antihypertensives in pregnancy, knowledge about target blood pressure in pregnancy hypertension, acknowledgement of medication side-effects and a positive interaction with the healthcare professional (including communication and approach to decision-making) (figure 2b).

*Adherence*

Internal conflict was an important determinant of non-adherence (figure 2a) as only the women who expressed conflict reported non-adherence to antihypertensive medication. Around half (8 of 18) the women interviewed described non-adherence to prescribed antihypertensives at some point during pregnancy with three women non-adherent at the time of interview (third trimester). However, nine of 14 women describing internal conflict were adherent at the time of interview which was mediated by the 'responsibility of motherhood' rather than concordance with the hypertension management plan (figure 2b).

Process of implementation (implementation strategies)

All three Trusts had a consultant obstetrician who led the care of women with chronic hypertension and could be considered the opinion leader. Two of three Trusts had a named midwife or team of midwives who specialised in the care of these women and were potential champions. However, influencers and champions were not always utilised to support guideline implementation. Further, as implementation of the guidelines had not been audited in any of the Trusts, although some outcome data was routinely collected and analysed, opportunities to address unwanted variance were being missed. These findings are supported by the national survey which found only a quarter of the Trusts collected and analysed the outcomes of women with chronic hypertension in pregnancy.

For peer review only



For peer review only - http://bmjopen.bmj.com/site/about/guidelines.xhtml

## DISCUSSION

Women in this study (14/18) reported conflict relating to the uptake of prescribed antihypertensives in pregnancy and in many cases (8/14) internal conflict resulted in non-adherence. The most commonly cited reasons for conflict were lack of information provision, the side-effects experienced from the medication, beliefs about safety of medication and uncertainty about normal blood pressure ranges in pregnancy. Adherence to antihypertensives in conflicted pregnant women was mediated through a responsibility to motherhood rather than through a trusting partnership with healthcare professionals (supported by information provision, management of side-effects and relational factors) as found in concordant adherent women. Despite this, our findings demonstrated that optimal information provision about antihypertensives and shared decision-making occurred infrequently during antenatal consultations. Our findings also illustrated that the implementation of blood pressure target setting was sub-optimal as a result of 'unshared' or undocumented target setting and in some cases an absence of target setting.

A major strength of the study is the use of multi-methodological approaches and an implementation framework in order to improve reliability, validity and generalisability. However, the study is limited by the population size for each of the methods and the limited statistical power for further analysis of the quantitative results. Results from the national survey may overstate compliance with national guidance. The survey was sent out to healthcare professionals from professional organisations; respondents were therefore self-selecting and may represent a relatively interested group of healthcare professionals. The non-response rate is also unknown. The structured observations were carried out using a validated tool with high interrater reliability.[11] However, the observations were carried out by one midwife researcher which may affect the validity of the findings. Finally, the purposive sampling of healthcare professionals providing routine antenatal care for women with chronic hypertension resulted in a focus on lead carers (consultant obstetricians, obstetric medicine specialists and named midwives) being interviewed, rather than doctors in training and midwives in acute areas such as the maternity assessment unit.

The emergence of implementation science in recent years has identified that a gap between research findings and clinical practice exists, and that clinical guideline production does not ensure evidence-based practices are routinely adopted.[15] A recent study in British Colombia evaluated the implementation of recently published pregnancy hypertension guidelines and its associated effect on maternal and perinatal outcomes.[16] Following guideline dissemination the study reported a fall of about a third in combined adverse maternal health outcomes (3.1% to 1.9%) but did not report a significant reduction in adverse perinatal outcomes.[16] However, the wanted and unwanted variance



For peer review only - http://bmjopen.bmj.com/site/about/guidelines.xhtml

in guidance uptake was not reported and the underlying mechanisms that influenced outcomes is not described. Our study uses an implementation framework by which variance in the implementation of existing guidelines could be described and mechanisms that support and hinder their uptake can be analysed, uniquely identifying strategies to improve the uptake of guidance and reduce maternal and fetal morbidity. Critically, although the NICE hypertension in pregnancy guidelines[6] have been recently updated, the core hypertension management recommendations remain unchanged, as do the quality statements. Therefore, the findings of this study remain important and relevant to those wanting to improve implementation.

The study also adds to the small body of antihypertensive adherence in pregnancy research that has found antihypertensive side-effects are a determinant of non-adherence. One recent randomised controlled trial identified 11% of those included in randomisation discontinued the antihypertensive due to side-effects.[17] Our study found about 40% of all women did not adhere to their prescribed antihypertensives at some point during pregnancy. This number compared more similarly to an internet-based study of 210 pregnant women undertaken in Europe, America and Australia which identified a 32.9% non-adherence rate in women taking cardiovascular medications in pregnancy.[18] These findings are supported by similar smaller questionnaire-based studies of pregnant women's medication adherence. [19 20] Our study may have identified higher rates of non-adherence due to the nature of qualitative interviewing that explore in-depth women's experiences and therefore unpick medication behaviours in a way that quantitative studies cannot.

Women's adherence to antihypertensives in pregnancy was found to be sub-optimal, and strategies to improve adherence are likely to reduce incidences of severe hypertension and prevent associated morbidity (and mortality). [21] These include improved information provision about anti-hypertensives and blood pressure targets as well as embedding shared decision-making into practice. Improvements in target blood pressure setting practices overall are also likely to reduce incidences of severe hypertension and prevent associated morbidity (and mortality). [3 5]

This study adds to the body of research that already exists outside of pregnancy which demonstrates that implementation of guidelines is not optimally achieved through the process of diffusion.[15] Although there was some evidence that some aspects of implementation were improved by having a specialist service for hypertension, this is likely to be most easily justified in areas where there is a high prevalence of chronic hypertension. Therefore, strategies to improve implementation in wider settings are required. Professionals require guideline updates, implementation toolkits (to improve target blood pressure setting practices, standardised information about antihypertensives and in consultation aids to support decision-making) but also need to buy into the evidence that underpins



For peer review only - http://bmjopen.bmj.com/site/about/guidelines.xhtml

the guidance. Maternal and perinatal outcomes, which includes episodes of severe hypertension, should be collected annually and used to support informed discussions about optimising antenatal care for this group of women.

Further research into the effectiveness and long-term safety of common antihypertensives in pregnancy to support evidenced-based guidelines is required. Future research may also wish to evaluate strategies to reduce women's conflict regarding their antihypertensive use in pregnancy and establish the effect of interventions on maternal concordance and health outcomes. However, without further evidence relating to the safety and effectiveness of common antihypertensives it is unclear if further reductions in maternal and fetal morbidity can be achieved through prescribing practices. Future research should also focus on active implementation of blood pressure target setting and pathways for those with outside of target blood pressure readings. This is likely to reduce morbidity as target blood pressure setting in pregnancy has been shown to reduce incidences of severe hypertension.[3 5] Policymakers may also wish to consider further studies that identify effective models and pathways of care for reducing adverse perinatal outcomes within the context of pregnancy hypertension.

## CONCLUSION

Maternal and neonatal morbidity resulting from severe hypertension in pregnancy is prevalent. [1 4 5] This evaluation of the implementation of the NICE hypertension in pregnancy guidelines (2010)[6] addresses strategies to reduce the number of episodes of severe hypertension and has identified sub-optimal target setting practices, poor information provision for pregnant women and variance in prescribing practices. Women's non-adherence to antihypertensives is higher than previously reported and this is likely to be contributing to adverse perinatal outcomes. Analysis of the domains that influence implementation of the guidelines have identified that education and decision-making strategies are needed to address both clinician and women's behaviour. Further research into the effectiveness and long-term safety of common antihypertensives is also required.



For peer review only - http://bmjopen.bmj.com/site/about/guidelines.xhtml

a. Contributorship statement – RW, LC and JS conceived of the study, the manuscript and analyses, with contributions from LW, JG, HB and HW. RW was responsible for data management and data analysis. All authors reviewed, critically revised and approved the manuscript.

b. Competing interests – None declared

c. Funding – This work was supported National Institute for Health Research (Research Professorship RP-2014-05-019) and by the National Institute for Health Research (NIHR) Collaboration for Leadership in Applied Health Research and Care South London (NIHR CLAHRC South London) at King's College Hospital NHS Foundation Trust.

d. Data sharing statement - All data relevant to the study are included in the article.

For peer review only



For peer review only - http://bmjopen.bmj.com/site/about/guidelines.xhtml

References

1. Bramham K, Parnell B, Nelson-Piercy C, et al. Chronic hypertension and pregnancy outcomes: systematic review and meta-analysis. *BMJ : British Medical Journal* 2014;348:g2301. doi: 10.1136/bmj.g2301

2. Knight M NM, Tuffnell D, Kenyon S, Shakespeare J, Brocklehurst P, Kurinczuk JJ (Eds.) on behalf of MBRRACE-UK. . Saving Lives, Improving Mothers' Care – Surveillance of maternal deaths in the UK 2012–14 and lessons learned to inform maternity care from the UK and Ireland Confidential Enquiries into Maternal Deaths and Morbidity 2009-14, 2016.

3. Magee LA, von Dadelszen P, Rey E, et al. Less-tight versus tight control of hypertension in pregnancy. *N Engl J Med* 2015;372(5):407-17. doi: 10.1056/NEJMoa1404595 [published Online First: 2015/01/30]

4. Flenady V, Koopmans L, Middleton P, et al. Major risk factors for stillbirth in high-income countries: a systematic review and meta-analysis. *Lancet* 2011;377(9774):1331-40. doi: 10.1016/S0140-6736(10)62233-7 [published Online First: 2011/04/19]

5. Magee LA, von Dadelszen P, Singer J, et al. The CHIPS Randomized Controlled Trial (Control of Hypertension in Pregnancy Study): Is Severe Hypertension Just an Elevated Blood Pressure? *Hypertension* 2016;68(5):1153-59. doi: 10.1161/HYPERTENSIONAHA.116.07862 [published Online First: 2016/09/14]

6. (NICE) NIfHCE. Hypertension in pregnancy CG107 2010 [Available from: https://www.nice.org.uk/guidance/cg107

7. (NICE) NIfHCE. Hypertension in pregnancy. Quality Standard (QS35), 2013.

8. (NICE) NIfHCe. Patient Experience in Adult NHS Services: Improving the Experience of Care for People Using Adult NHS Services: Patient Experience in Generic Terms. London2012.

9. Kirk MA, Kelley C, Yankey N, et al. A systematic review of the use of the Consolidated Framework for Implementation Research. *Implementation Science* 2016;11(1):72. doi: 10.1186/s13012-016-0437-z

10. Hoffmann TC, Glasziou PP, Boutron I, et al. Better reporting of interventions: template for intervention description and replication (TIDieR) checklist and guide. *BMJ : British Medical Journal* 2014;348:g1687. doi: 10.1136/bmj.g1687

11. Burt J, Abel G, Elmore N, et al. Assessing communication quality of consultations in primary care: initial reliability of the Global Consultation Rating Scale, based on the Calgary-Cambridge Guide to the Medical Interview. *BMJ Open* 2014;4(3):e004339. doi: 10.1136/bmjopen-2013-004339



For peer review only - http://bmjopen.bmj.com/site/about/guidelines.xhtml

12. Damschroder LJ, Aron DC, Keith RE, et al. Fostering implementation of health services research findings into practice: a consolidated framework for advancing implementation science. *Implementation science : IS* 2009;4:50. doi: 10.1186/1748-5908-4-50 [published Online First: 2009/08/12]

13. International consortium for health outcome measurements i. Pregnancy and childbirth standard set. 2016

14. Pope C, Ziebland S, Mays N. Qualitative research in health care. Analysing qualitative data. *BMJ* 2000;320(7227):114-16. doi: 10.1136/bmj.320.7227.114

15. Rangachari P, Rissing P, Rethemeyer K. Awareness of evidence-based practices alone does not translate to implementation: insights from implementation research. *Quality management in health care* 2013;22(2):117-25. doi: 10.1097/QMH.0b013e31828bc21d [published Online First: 2013/04/02]

16. von Dadelszen P, Sawchuck D, McMaster R, et al. The Active Implementation of Pregnancy Hypertension Guidelines in British Columbia. *Obstetrics & Gynecology* 2010;116(3):659-66. doi: 10.1097/AOG.0b013e3181eb669d

17. Webster Louise M, Myers Jenny E, Nelson-Piercy C, et al. Labetalol Versus Nifedipine as Antihypertensive Treatment for Chronic Hypertension in Pregnancy. *Hypertension* 2017;70(5):915-22. doi: 10.1161/HYPERTENSIONAHA.117.09972

18. Lupattelli A, Spigset O, Nordeng H. Adherence to medication for chronic disorders during pregnancy: results from a multinational study. *International Journal of Clinical Pharmacy* 2014;36(1):145-53. doi: 10.1007/s11096-013-9864-y

19. Matsui D. Adherence with Drug Therapy in Pregnancy. *Obstetrics and Gynecology International* 2012;2012:5. doi: 10.1155/2012/796590

20. Abheiden CNH, van Reuler AVR, Fuijkschot WW, et al. Aspirin adherence during high-risk pregnancies, a questionnaire study. *Pregnancy Hypertension: An International Journal of Women's Cardiovascular Health* 2016;6(4):350-55. doi: https://doi.org/10.1016/j.preghy.2016.08.232

21. Abalos E, Duley L, Steyn DW, et al. Antihypertensive drug therapy for mild to moderate hypertension during pregnancy. *Cochrane Database Syst Rev* 2018;10:CD002252. doi: 10.1002/14651858.CD002252.pub4 [published Online First: 2018/10/03]

For peer review only



For peer review only - http://bmjopen.bmj.com/site/about/guidelines.xhtml

505 Figure 1. Interpretation of integrated analysis: a strategy for improved implementation of evidence-based hypertension in pregnancy management

507 Figure 2a. Women's adherence and concordance with prescribed antihypertensives. 2b. Facilitators of women's adherence and of concordance.

For peer review only



For peer review only - http://bmjopen.bmj.com/site/about/guidelines.xhtml

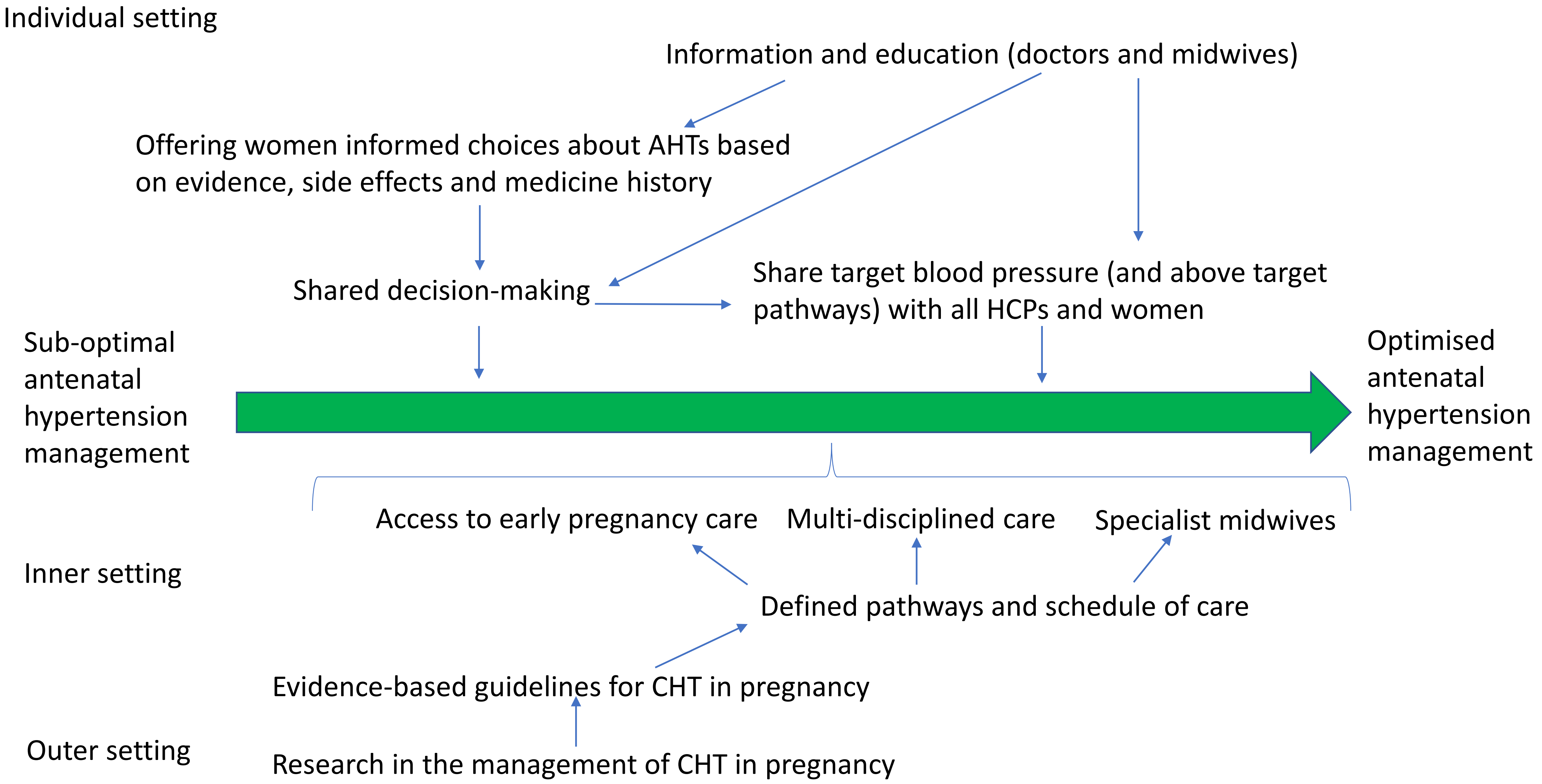
Individual setting
Information and education (doctors and midwives)
Offering women informed choices about AHTs based on evidence, side effects and medicine history
Shared decision-making
Share target blood pressure (and above target pathways) with all HCPs and women
Sub-optimal antenatal hypertension management
Optimised antenatal hypertension management
Access to early pregnancy care
Multi-disciplined care
Specialist midwives
Inner setting
Defined pathways and schedule of care
Evidence-based guidelines for CHT in pregnancy
Outer setting
Research in the management of CHT in pregnancy

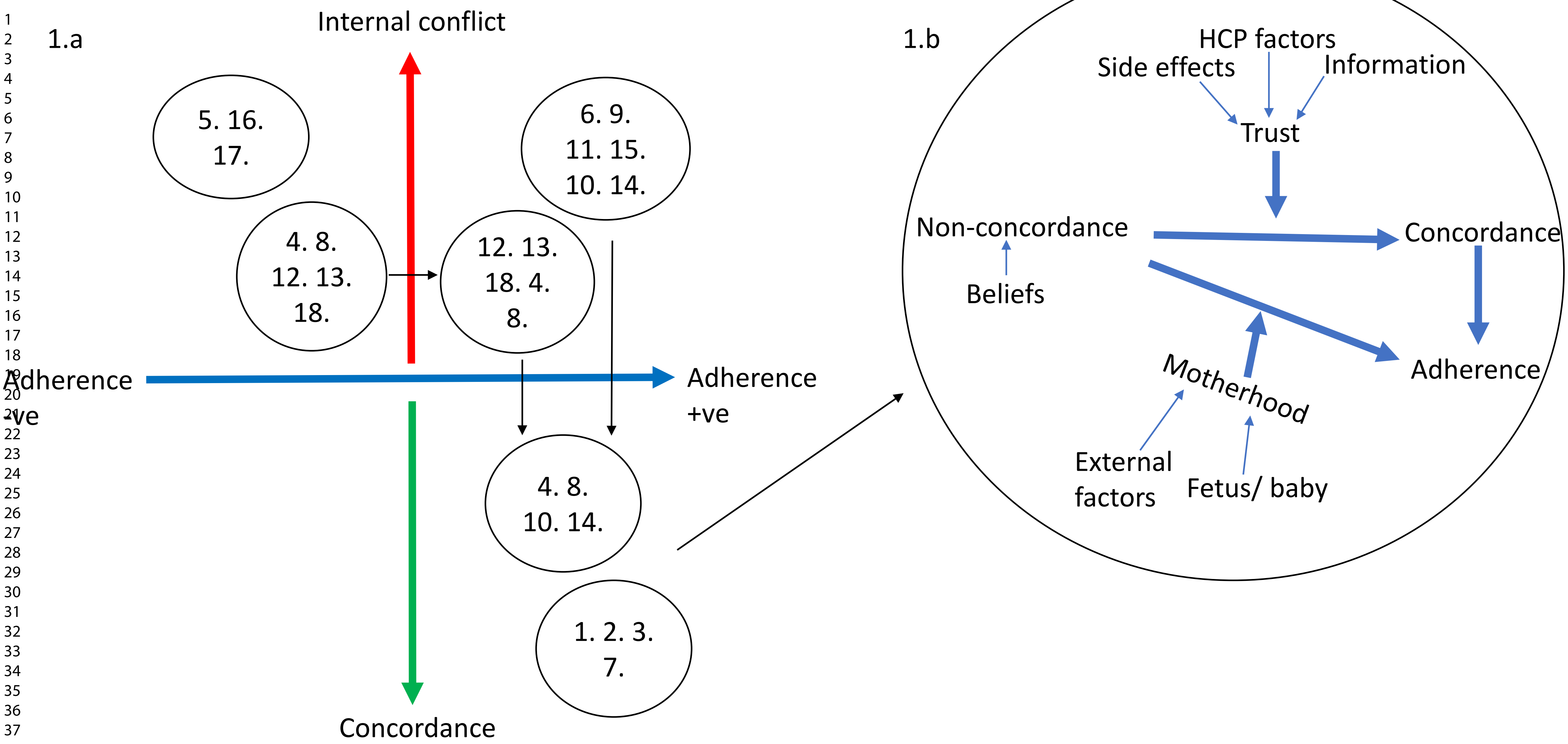
1.a
Internal conflict
5. 16. 17.
6. 9. 11. 15. 10. 14.
4. 8. 12. 13. 18.
12. 13. 18. 4. 8.
Adherence -ve
Adherence +ve
4. 8. 10. 14.
1. 2. 3. 7.
Concordance
1.b
HCP factors
Side effects
Information
Trust
Non-concordance
Concordance
Beliefs
Motherhood
Adherence
External factors
Fetus/ baby

Supplementary file 1

Chronic hypertension in pregnancy – healthcare professional survey

| Respondents | Number (97) | Percentage % |
|---|---|---|
| Obstetrician<br>Of which are consultants | 69<br>53 | 71.1<br>55 |
| Midwife<br>Of which are specialist/ senior midwife | 28<br>22 | 28.9<br>22.7 |
| NHS hospital trusts represented<br>(including England, Ireland, Scotland and Wales) | 69 | - |

Question 1: If you see a pregnant woman with chronic hypertension who is currently taking either ACEIs or ARBs (e.g. at the beginning of pregnancy), how often would you ask her to stop taking them?

| Response | Number (97) | Percentage (%) |
|---|---|---|
| Always | 57 | 57.8 |
| Almost always | 27 | 27.8 |
| About two thirds of the time | 1 | 1 |
| About half of the time | 4 | 4.1 |
| About a third of the time | 0 | 0 |
| Very rarely | 1 | 1 |
| Never | 3 | 3.1 |
| Missing | 4 | 4.1 |

Question 2: What do you usually use as your first line anti-hypertensive treatment(s) for women with chronic hypertension in pregnancy?

| Anti-hypertensive | Number (97) | Percentage (%) |
|---|---|---|
| Labetalol | 85 | 87.6 |
| Methyldopa | 29 | 29.9 |
| Nifedipine | 32 | 33.0 |
| Amlodipine | 2 | 2.1 |

Question 3: What additional anti-hypertensive medication do you use for treating women with chronic hypertension in pregnancy?

| Anti-hypertensive | Number (97) | Percentage (%) |
|---|---|---|
| Amlodipine | 37 | 38.1 |
| Atenolol | 2 | 2.1 |
| Doxazosin | 23 | 23.7 |
| Enalapril | 1 | 1.0 |
| Hydralazine (oral) | 2 | 2.1 |
| Labetalol | 38 | 39.2 |
| Methyldopa | 60 | 61.9 |
| Metoprolol | 1 | 1.0 |
| Nifedipine | 79 | 81.4 |

For peer review only

Question 4: How frequently do you set a blood pressure target for women with chronic hypertension in pregnancy who need anti-hypertensive treatment (assuming no other co-morbidity) (mmHg)?

| **Answer** | **Number (97)** | **Percentage %** |
|---|---|---|
| Always | 36 | 37.1 |
| Almost always | 36 | 37.1 |
| About two thirds of the time | 8 | 8.2 |
| About half of the time | 3 | 3.1 |
| About a third of the time | 4 | 4.1 |
| Very rarely | 3 | 3.1 |
| Never | 1 | 1.0 |
| Other<br>In the guidelines but compliance unknown<br>Frequency not described | 6<br>2<br>4 | 6.2 |

Question 5: What blood pressure target do you usually set for pregnant women with chronic hypertension (assuming no other co-morbidity) (mmHg)?

| **Systolic** | **Number (97)** | **Percentage %** | **Median (IQR1-IQR3)** |
|---|---|---|---|
| 120 | 2 | 2.1 | |
| 125 | 0 | 0.0 | |
| 130 | 6 | 6.2 | |
| 135 | 2 | 2.1 | |
| 140 | 33 | 34.0 | |
| 145 | 0 | 0.0 | |
| 150 | 40 | 41.2 | |
| 155 | 1 | 1.0 | |
| 160 | 8 | 8.2 | |
| Missing | 4 | 4.1 | |
| Median | | | 150 (140-150) |

| **Diastolic** | **Number (97)** | **Percentage %** | **Median (IQR1-IQR3)** |
|---|---|---|---|
| 80 | 9 | 9.3 | |
| 85 | 7 | 7.2 | |
| 90 | 37 | 38.1 | |
| 95 | 8 | 8.2 | |
| 100 | 27 | 27.8 | |
| 110 | 3 | 3.1 | |
| Missing | 5 | 5.2 | |
| Median | | | 90 (90-100) |

For peer review only

Question 6: How often do you prescribe Aspirin for women with chronic hypertension in pregnancy?

| Answer | Number (97) | Percentage % |
|---|---|---|
| Always | 53 | 54.6 |
| Almost always | 36 | 37.1 |
| About two thirds of the time | 5 | 5.2 |
| About half of the time | 2 | 2.1 |
| Very rarely | 1 | 1.0 |

Question 7: At what gestation do these women usually receive their first Aspirin prescription?

| Answer | Number (97) | Percentage % |
|---|---|---|
| Before 12 weeks | 41 | 42.3 |
| 12-15+6 weeks | 52 | 53.6 |
| 16-19+6 weeks | 1 | 1.0 |
| Missing answer | 3 | 3.1 |

Question 8: For a woman with uncomplicated chronic hypertension in pregnancy (i.e. no additional risk factors), how many routine fetal growth do they receive (excluding nuchal and anomaly scans)?

| Additional scans | Number (97) | Percentage % | Median (IQR1-IQR3) |
|---|---|---|---|
| None | 4 | 4.1 | |
| 1 | 12 | 12.4 | |
| 2 | 23 | 23.7 | |
| 3 | 37 | 38.1 | |
| 4 | 21 | 21.6 | |
| >4 | 1 | 1.0 | |
| | | | 3 (2-3) |

Question 9: When do you usually plan birth for women with chronic hypertension whose blood pressure is controlled below 160/110?

| Gestation | Number (97) | Percentage (%) | Median (IQR1-IQR3) |
|---|---|---|---|
| Before 34 weeks | 3 | 3.1 | |
| 34-34+6 weeks | 2 | 2.1 | |
| 35-35+6 weeks | 2 | 2.1 | |
| 36-36+6 weeks | 4 | 4.1 | |
| 37-37+6 weeks | 27 | 27.8 | |
| 38-38+6 weeks | 36 | 37.1 | |
| 39-39+6 weeks | 41 | 42.3 | |
| 40-41 weeks | 28 | 28.9 | |
| Await spontaneous labour | 5 | 5.2 | |
| Other – individualised | 4 | 4.2 | |
| | | | 38.5 (37-39) |

For peer review only

Question 10: Involving pregnant women who have chronic hypertension in their pregnancy and birth planning is an important part of the consultation?

| Sentiment | Number (97) | Percentage (%) |
|---|---|---|
| Agree Strongly | 79 | 81.4 |
| Agree | 15 | 15.5 |
| Slightly Agree | 2 | 2.1 |
| Slightly disagree | 0 | 0.0 |
| Disagree | 0 | 0.0 |
| Disagree Strongly | 1 | 1.0 |

Question 11: If you wish, can you give an example of how you enable women to be actively involved in their care?

| Themes | Number (47) | Percentage % |
|---|---|---|
| Total responses | 47 | |
| SDM in the following areas<br>• Home BP<br>• Monitoring BP<br>• Anti-hypertensives<br>• Planning birth (IOL)<br>• Organisation of care | <br>10<br>6<br>5<br>17<br>4 | <br>21<br>12.8<br>10.6<br>36<br>8.5 |
| Discussing risks and benefits | 2 | 4.3 |
| How to identify pre-eclampsia | 2 | 4.3 |

Question 12: In your maternity unit what term/s best describes the antenatal care provided to most women with chronic hypertension?

| Care provision | Number (97) | Percentage % |
|---|---|---|
| Named consultant-led general antenatal clinic (maternal medicine clinic) | 63 (7) | 64.9 (7.2) |
| Consultant-led specialist hypertension in pregnancy clinic | 25 | 25.8 |
| Multi-disciplinary clinic with additional medical professional | 20 | 20.6 |
| Consultant obstetrician and midwife antenatal clinic | 15 | 15.5 |
| Shared-care GP/ obstetrician/ midwife | 7 | 7.2 |
| Specialist midwifery care (e.g. medical conditions team) | 6 | 6.2 |
| Hospital midwifery care | 1 | 1.0 |
| Community based midwifery care | 4 | 4.1 |
| Day assessment unit | 2 | 2.1 |

For peer review only

Question 13: In your maternity unit when do the pregnant women with chronic hypertension usually first get seen by an obstetrician?

| Gestation | Number (97) | Percentage % |
|---|---|---|
| Before 12 weeks | 24 | 24.7 |
| 12-15+6 weeks | 63 | 64.9 |
| 16-27+6 weeks | 9 | 9.3 |
| Missing data | 1 | 1.0 |

Question 14: Do you or someone in your unit specifically collect and analyse the outcomes of women with chronic hypertension in pregnancy annually?

| Response | Number (97) | Percentage (%) |
|---|---|---|
| Yes | 24 | 24.7 |
| No | 67 | 69.0 |
| Unsure | 4 | 4.1 |
| Some aspects | 2 | 2.0 |

For peer review only

Supplementary file 2

Demographics of women

| **Women demographics** | **Observed n=28 (%)** | **Interviewed n=18 (%)** | **Case notes n=55 (%)** |
|---|---|---|---|
| **Ethnicity** | | | |
| White British | 9 (32.0) | 7 (39.0) | 15 (27.3) |
| White Other | 6 (21.0) | 4 (22.0) | 8 (14.5) |
| Black | 9 (32.0) | 5 (28.0) | 18 (32.7) |
| Asian | 2 (7.0) | 1 (5.5) | 8 (14.5) |
| Any other | 2 (7.0) | 1 (5.5) | 6 (10.9) |
| **Parity at booking** | | | |
| 0 | 9 (32.0) | 7 (39.0) | 15 (27.3) |
| 1 | 11(39.0) | 7 (39.0) | 21 (38.2) |
| 2 | 7 (25.0) | 4 (22.0) | 10 (18.2) |
| 3 | 0 (0.0) | 0 (0.0) | 6 (10.9) |
| 4 | 0 (0.0) | 0 (0.0) | 2 (3.6) |
| 5 | 1 (4.0) | 0 (0.0) | 1 (1.8) |
| **Age** | | | |
| <20 | 0 (0.0) | 0 (0.0) | 0 (0.0) |
| 20-34 | 17 (61.0) | 11 (61.0) | 23 (41.8) |
| 35-39 | 7 (25.0) | 5 (28.0) | 21 (38.9) |
| 40-44 | 4 (14.0) | 2 (11.0) | 11 (20.4) |
| 45-49 | 0 (0.0) | 0 (0.0) | 0 (0.0) |
| **BMI** | | | |
| <18.5 | 0 (0.0) | 0 (0.0) | 1/52 (1.9) |
| 18.5-24.9 | 7 (25) | 6 (33.3) | 13/52 (25.0) |
| 25-29.9 | 10 (36) | 6 (33.3) | 13/52 (25.0) |
| 30-34.9 | 9 (32) | 5 (28.0) | 11/52 (21.2) |
| 35-39.0 | 2 (7) | 1 (5.5) | 6/52 (11.5) |
| >40.0 | 0 (0) | 0 (0.0) | 8/52 (7.7) |

For peer review only

Supplementary file 3

Pregnancy and birth outcomes – Case notes review

| **Outcomes** | **Case notes review**<br>**Nominator/denominator (%)** |
|---|---|
| Women with episode of severe hypertension | 25/55 (45.5) |
| 1st trimester episode | 2/40 (5.0) |
| 2nd trimester episode | 13/40 (32.5) |
| 3rd trimester episode | 25/40 (62.5) |
| Birth weight - median<br>(IQR1 – IQR3) | 2927.5<br>(2592.5 - 3200) |
| Admission to NNU | 9/55 (16.4) |

For peer review only

Supplementary file 4

Target blood pressure setting and prescribing practices per Trust

| | **Hospital Trust 1 n=29 (%)** | **Hospital Trust 2 n=13 (%)** | **Hospital Trust 3 n=13 (%)** |
|---|---|---|---|
| **Target BP documented <150/100mmHg** | 20/26 (77.0) | 3/13 (23.0) | 5 (38.0) |
| **Labetalol** | 12/26 (46.0) | 7/12 (58.3) | 9/11 (82.0) |
| **Nifedipine** | 9/26 (34.5) | 0/12 (0.0) | 0/11 (0.0) |
| **Methyldopa** | 3/26 (11.5) | 4/12 (33.3) | 1/11 (9.0) |
| **Other** | 2/26 (8.0) | 1/12 (8.3) | 1/11 (9.0) |

For peer review only

Original research

# The quality of mixed methods studies in health services research

**Alicia O'Cathain, Elizabeth Murphy[1], Jon Nicholl**

Medical Care Research Unit, School of Health and Related Research, University of Sheffield, Sheffield; [1]School of Sociology and Social Policy, University of Nottingham, Nottingham, UK

***Objectives*: To assess the quality of mixed methods studies in health services research (HSR).**

***Methods*: We identified 118 mixed methods studies funded by the Department of Health in England between 1994 and 2004, and obtained proposals and/or final reports for 75. We applied a set of quality questions to both the proposal and report of each study, addressing the success of the study, the mixed methods design, the individual qualitative and quantitative components, the integration between methods and the inferences drawn from completed studies.**

***Results*: Most studies were completed successfully. Researchers mainly ignored the mixed methods design and described only the separate components of a study. There was a lack of justification for, and transparency of, the mixed methods design in both proposals and reports, and this had implications for making judgements about the quality of individual components in the context of the design used. There was also a lack of transparency of the individual methods in terms of clear exposition of data collection and analysis, and this was more a problem for the qualitative than the quantitative component: 42% (19/45) versus 18% (8/45) of proposals ($p = 0.011$). Judgements about integration could rarely be made due to the absence of an attempt at integration of data and findings from different components within a study.**

***Conclusions*: The HSR community could improve mixed methods studies by giving more consideration to describing and justifying the design, being transparent about the qualitative component, and attempting to integrate data and findings from the individual components.**

*Journal of Health Services Research & Policy* Vol 13 No 2, 2008: 92–98 © The Royal Society of Medicine Press Ltd 2008

## Introduction

Mixed methods studies are common in health services research (HSR).[1] They consist of two separate components of data collection and analysis within a single study: at least one quantitative method with structured data collection and statistical analysis, and at least one qualitative method with less structured data collection and thematic analysis.[2] Commissioners and consumers of research, as well as researchers themselves, need to judge whether a mixed methods study has been undertaken well or poorly, assessing whether it is good mixed methods research as well as good research. The quality of mixed methods research has been considered explicitly in health, educational and social research,[3–8] and implicitly when researchers have discussed the challenges of designing and implementing these studies.[9,10] However, the issue has received little consideration overall, with a recent search for quality criteria for mixed methods research concluding that there were none available,[7] even though attempts have been made to develop them.[3] Given that there are no agreed criteria for assessing the quality of these studies,[8] and that researchers are still debating the meaning of quality for mixed methods research,[6] it is premature to attempt to develop definitive criteria. Instead, it seems sensible to follow an approach taken by researchers considering quality in the context of synthesizing qualitative and quantitative evidence[11] and devise a set of questions which could be applied to mixed methods primary research to facilitate judgements about quality. We devised a set of 'quality questions' and applied them to proposals and reports of mixed methods studies to assess the quality of mixed methods studies in HSR.

## Methods

This research was part of a wider study exploring the use of mixed methods research in HSR. The wider study consisted of a quantitative documentary analysis of 75 mixed methods studies to determine the type

**Alicia O'Cathain PhD**, MRC Fellow, **Jon Nicholl MSc**, Professor, Medical Care Research Unit, School of Health and Related Research, University of Sheffield, Regent Street, Sheffield S1 4DA, UK; **Elizabeth Murphy PhD**, Professor, School of Sociology and Social Policy, University of Nottingham, Nottingham, UK.

Correspondence to: a.ocathain@sheffield.ac.uk

 DOI: 10.1258/jhsrp.2007.007074

For peer review only - http://bmjopen.bmj.com/site/about/guidelines.xhtml

and quality of mixed methods research undertaken, and qualitative interviews with 20 researchers to explore facilitators and barriers to exploiting the potential of this approach.[1,12]

## Devising questions about quality

We devised a framework for the quality assessment based on detailed consideration of the literature on mixed methods research in the fields of health, social and educational research. We searched the health databases MEDLINE and CINAHL. We then sought expert opinion encapsulated in key textbooks.[10,13–20] Finally we searched the Social Science Citations Index, PsycINFO, ERIC and the British Education Index to identify social, behavioural and educational research. The search for literature took place in 2003 and was updated in 2006. Quality was one of 11 issues identified in this review.

Within the literature, one suggested assessment criterion for mixed methods studies was whether they had been completed successfully in terms of adequately addressing the research questions with allocated resources.[5] Other researchers focused on the quality of methods. There was no suggestion of using a tool developed for generic use across all designs. Rather, researchers attempted to develop quality criteria by devising separate lists of criteria for the quantitative and the qualitative research.[7] Their assumption was that methods are linked to paradigms and therefore the criteria used to assess different methods should also be linked to paradigms.[7] However, not everyone agrees that methods are paradigm-specific[18] or that different criteria are needed for qualitative and quantitative research.[21] The same criteria have been proposed for both[21] although the appropriate means for judging against these criteria may differ because of the research practices employed in different methodological approaches. The mixed methods design[10] and the integration between methods[3] can be assessed as well as the individual methods. A good mixed methods study clearly justifies why a mixed methods approach is necessary or superior to another, offers transparency of the mixed methods design, and offers appropriate sampling, data collection and analysis of individual components relating to that design.[3,4,10] Thus the design may determine the criteria used to make judgements about the individual components of the study. Integration of data or findings from each component is a key part of mixed methods research,[10] distinguishing it from qualitative and quantitative studies undertaken independently. When integration occurs, it is important that data transformations are defensible, that contradictory findings are explained and convergent findings are not related to shared bias between methods.[3] Expertise may be needed within a research team to integrate at the analysis stage.[22] Finally, researchers have discussed the importance of inferences from mixed methods studies being trustworthy[6] and appropriate in the light of the design used.[3] As yet there are no criteria for assessing the quality of inferences from mixed methods research, although researchers are considering the complexity of this issue.[23]

When developing the framework for our quality questions we chose not to use a generic tool because they have variable applicability across different research designs.[24] We chose to assess the qualitative and quantitative components separately because they each contribute to the study as a whole and because the quality of one or both components may suffer as a consequence of being part of a mixed methods study.[25–27] In addition to the individual components, we included an assessment of the success of the study, the design, the integration and the inferences. Within this framework we constructed questions based on the literature review and reading the proposals and reports from four mixed methods studies in HSR.

## Identifying mixed methods studies

In 2004, mixed methods studies were identified through a systematic search of summaries of studies funded by the Department of Health, a key commissioner of health services research in England at that time. The methods have been described elsewhere[1,12] and are summarized here. Summaries of single studies funded between 1994 and 2004 through 10 programmes were read. The programmes were: Health Technology Assessment; Service Delivery and Organization; New and Emerging Applications of Technology; Policy Research Programme; and the NHS Research & Development programmes of maternal and child health, primary and secondary care interface, cardiovascular disease and stroke, forensic mental health, primary dental care, and promoting implementation of research findings. A total of 118 mixed methods studies were identified. The lead researcher of each study was written to with a request for the research proposal, the final report for completed studies and a list of any emerging publications.

## Application of quality questions

A data extraction form was devised which consisted of the quality questions with the tick box options of 'yes', 'yes, but improvements are possible', 'no', 'not enough information (NEI)' and 'not applicable (N/A)'. Space for open comments was available alongside each question, where the assessor (AOC) could record details of good and poor practice. The data extraction form was applied to each study by one researcher, first to the proposal and then to the report. Finally, any differences between the proposal and report were noted.

## Analysis

The structured data were entered into SPSS. The main analysis was descriptive, displaying the proportions of proposals and reports falling into each category of each question. The chi-squared test was used when

For peer review only - http://bmjopen.bmj.com/site/about/guidelines.xhtml

comparing results for the individual qualitative and quantitative components. Open comments were quantitized[28] by transcribing them into Word, grouping them into themes, and counting the number of studies in which a theme occurred.[29]

## Results

Documentation was received for 75 mixed methods studies. Full proposals were obtained for 60% (45/75) of the studies. Final reports were only available for the 52 studies completed by the time of data collection, and were obtained for 92% (48), although one was a summary report that was too brief for inclusion in the assessment of quality, leaving 47 reports. Both a proposal and report was available for 20 studies.

### Success

The potential to produce a successfully completed study was assessed using the research proposals. In most proposals, the quantitative methods appeared to be feasible within the time and money allocated (Table 1). However, even recognizing that some aspects of qualitative research cannot be fixed at the design stage (e.g. sample size for theoretical sampling), there was not enough detail to determine the feasibility of the qualitative methods in one-third of studies – for example, no indication of numbers of interviews to be undertaken or no indication of when the qualitative research would be conducted in the study timetable. We had concerns about the feasibility of the qualitative component in another one-third of proposals. From the open comments we identified 14 proposals where a large number of qualitative interviews were planned in a short time scale – for example, 40 interviews in four months without specifying the depth of interview and analysis. In nine of these studies the report was available and in four cases considerably fewer interviews were undertaken than planned. However, concerns highlighted about the feasibility of the qualitative research did not necessarily translate into shortfalls in the final study.

We defined a successful study as one that produced everything that had been planned at the proposal stage. A direct comparison of the final study report with the proposal was only possible on the subset of 20 studies for which both were available. In other cases the assessment relied on researchers detailing the planned and implemented study within their final report. Non-completion of a whole component of a study was rare (Table 1). However, in one-fifth of reports, one of the methods within a component was not executed as planned. This tended to be due to a range of problems in the field.

### Mixed methods design

A justification for using mixed methods research was only given in one-third of proposals and reports (Table 2). A minority of studies explicitly articulated the design in terms of the priority of methods, the purpose of combining methods, the sequence of methods and the stage at which integration would or did occur. It was particularly helpful for the subsequent quality assessment of individual components if researchers were explicit about the priority of methods and the role of any less dominant method. For example, it seemed inappropriate to have 40 in-depth interviews as a preliminary aid to develop a questionnaire, but appropriate if these interviews were also to be used as a primary means of investigating the issue under study. A lack of transparency of the overall design could occur in the context of excellent description of individual components.

When the design was not discussed explicitly it was usually possible to work out the key elements from reading the documentation. In most cases the design was assessed as appropriate for addressing the research question. However, researchers rarely discussed issues of rigour in relation to the design employed. An example of addressing rigour for the design was where researchers proposed that qualitative findings would not be shared with quantitative colleagues undertaking a randomized controlled trial to minimize the possibility of contamination of that trial; in another two studies, the qualitative research was undertaken with people not participating in the trial in order to avoid contaminating the trial. While the extent to which this attention to contamination avoidance was necessary may be debatable, it constitutes some evidence that researchers had given serious consideration to design issues related to mixed methods research.

**Table 1** Assessment of the success of mixed methods studies in HSR

| | Proposal (*n* = 45) | | | | Report (*n* = 47) | | | |
|---|---|---|---|---|---|---|---|---|
| | Yes | Yes, but | No | NEI or N/A | Yes | Yes, but | No | NEI or N/A |
| 1 Is the quantitative component feasible? | 82% | 2% | 4% | 11% | | | | |
| 2 Is the qualitative component feasible? | 38% | 20% | 13% | 29% | | | | |
| 3 Is the mixed methods design feasible? | 51% | 0% | 7% | 42% | | | | |
| 4 Have both qualitative and quantitative components been completed? | | | | | 87% | 6% | 2% | 4% |
| 5 Were some quantitative methods planned but not executed? | | | | | 19% | 0% | 45% | 36% |
| 6 Were some qualitative methods planned but not executed? | | | | | 21% | 2% | 38% | 38% |
| 7 Did the mixed methods design work in practice? | | | | | 85% | 0% | 2% | 13% |

NEI, not enough information; N/A, not applicable


For peer review only - http://bmjopen.bmj.com/site/about/guidelines.xhtml

**Table 2** Assessment of the mixed methods design of studies in HSR

| | Proposal (*n* = 45) | | | | Report (*n* = 47) | | | |
|---|---|---|---|---|---|---|---|---|
| | Yes | Yes, but | No | NEI or N/A | Yes | Yes, but | No | NEI or N/A |
| 1 Is the use of mixed methods research justified? | 31% | 3% | 60% | 4% | 30% | 2% | 66% | 2% |
| 2 Is the design for mixing methods described? | | | | | | | | |
| Priority | 16% | 2% | 78% | 4% | 15% | 0 | 83% | 2% |
| Purpose | 42% | 0 | 53% | 4% | 34% | 4% | 60% | 2% |
| Sequence | 56% | 0 | 40% | 4% | 49% | 0 | 49% | 2% |
| Stage of integration | 24% | 0 | 71% | 4% | 21% | 0 | 77% | 2% |
| 3 Is the design clearly communicated? | 80% | 0 | 16% | 4% | 81% | 4% | 9% | 6% |
| 4 Is the design appropriate for addressing the research questions? | 87% | 2% | 2% | 9% | 87% | 0% | 2% | 11% |
| 5 Has rigour of the design been considered (proposal) or adhered to (report)? | 7% | 0 | 93% | 0% | 21% | 0% | 0% | 79% |

NEI, not enough information; N/A, not applicable

## Quantitative component

The roles of the quantitative methods were usually communicated well within proposals and reports (Table 3). However, sufficient details were sometimes not given about these methods. In eight proposals the quantitative methods were only sketchily described and in a further 13 proposals some aspects of the quantitative methods were not described, in particular, the analysis (8) and the numbers involved (5). This was less of an issue for reports but nonetheless there were still problems with sketchy description overall (4) or little or no description of the analysis (5). This lack of transparency made it difficult to assess other aspects of quality.

Validity of the methods within the quantitative components was assessed by considering the attention researchers gave to issues such as confounding and bias. Validity was explicitly discussed in two-thirds of proposals, with little evidence that the rigour of any method was compromised (Table 3). There were few examples of an individual method being compromised by the mixed methods approach. One example was a Delphi exercise which was restricted in order to fit the timetable of the qualitative fieldwork.

It was difficult to determine the sophistication of proposed analyses due to the lack of detail about analysis in the research proposals. There was more information about analyses available in research reports and here concerns were identified about the sophistication of one-quarter of quantitative analyses. We identified 12 studies where the reported quantitative results seemed simplistic, sometimes only presenting descriptive statistics with no statistical tests and in two cases using an experimental design which was then ignored in the analysis.

## Qualitative component

The roles of the qualitative methods were usually communicated well within proposals and reports (Table 4). However, qualitative methods were often not described in sufficient detail and this occurred more frequently than for the quantitative components, both within proposals ($p = 0.011$) and reports ($p = 0.08$). First, there was sketchy description of the qualitative methods overall (15 proposals and 11 reports). In three of these reports there was no description of the qualitative methods at all, only the findings. Second, there were no details about an important aspect of the qualitative research, particularly the analysis (six proposals and nine reports). Third, one method was described in detail, usually interviews with a particular group, but a further qualitative method such as observation or focus groups appeared to be 'tagged on' with no description (six proposals). Fourth, the overall size of the qualitative component was not clear, with a few

For peer review only

**Table 3** Assessment of the quantitative component of mixed methods studies in HSR

| | Proposal (*n* = 45) | | | | Report (*n* = 47) | | | |
|---|---|---|---|---|---|---|---|---|
| | Yes | Yes, but | No | NEI or N/A | Yes | Yes, but | No | NEI or N/A |
| 1 Is the role of each method clear? | 98% | 0% | 2% | 0% | 96% | 2% | 0% | 2% |
| 2 Is each method described in sufficient detail? | 53% | 29% | 18% | 0% | 68% | 13% | 15% | 4% |
| 3 Is each method appropriate for addressing the research question? | 93% | 0 | 2% | 4% | 98% | 0% | 0% | 2% |
| 4 Is the approach to sampling and analysis appropriate for its purpose? | 67% | 4% | 4% | 24% | 70% | 9% | 6% | 15% |
| 5 Is there expertise among applicants/authors? | 67% | 2% | 7% | 24% | 30% | 0% | 0% | 70% |
| 6 Is there expertise on the team to undertake each method? | 60% | 0% | 2% | 24% | | | | |
| 7 Have issues of validity been addressed for each method? | 64% | 0% | 30% | 7% | 49% | 4% | 40% | 6% |
| 8 Has the rigour of any method been compromised? | 7% | 0% | 91% | 2% | 9% | 4% | 83% | 4% |
| 9 Is each method sufficiently developed for its purpose? | 84% | 0% | 7% | 9% | 83% | 0% | 4% | 13% |
| 10 Is the (intended) analysis sufficiently sophisticated? | 56% | 4% | 2% | 38% | 51% | 15% | 25% | 9% |

NEI, not enough information; N/A, not applicable

For peer review only - http://bmjopen.bmj.com/site/about/guidelines.xhtml

**Table 4** Assessment of the qualitative component of mixed methods studies in HSR

| | Proposal (*n* = 45) | | | | Report (*n* = 47) | | | |
|---|---|---|---|---|---|---|---|---|
| | Yes | Yes, but | No | NEI or N/A | Yes | Yes, but | No | NEI or N/A |
| 1 Is the role of each method clear? | 87% | 0% | 9% | 4% | 92% | 4% | 4% | 0% |
| 2 Is each method described in sufficient detail? | 24% | 29% | 42% | 4% | 38% | 28% | 30% | 4% |
| 3 Is each method appropriate for addressing the research question? | 87% | 7% | 2% | 4% | 91% | 2% | 2% | 4% |
| 4 Is the approach to sampling and analysis appropriate for its purpose? | 42% | 4% | 9% | 40% | 53% | 9% | 4% | 34% |
| 5 Is there expertise among the applicants/authors? | 56% | 2% | 11% | 31% | 32% | 4% | 0% | 64% |
| 6 Is there expertise on the team to undertake each method? | 44% | 9% | 7% | 40% | | | | |
| 7 Have issues of validity been addressed for each method? | 24% | 0% | 64% | 11% | 30% | 2% | 57% | 11% |
| 8 Has the rigour of any method been compromised? | 2% | 0% | 91% | 7% | 6% | 2% | 81% | 11% |
| 9 Is each method sufficiently developed for its purpose? | 64% | 0% | 9% | 27% | 77% | 2% | 9% | 13% |
| 10 Is the (intended) analysis sufficiently sophisticated? | 40% | 4% | 7% | 49% | 51% | 13% | 19% | 17% |

NEI, not enough information; N/A, not applicable

interviews here and there throughout the study adding up to a sizeable qualitative component of over 100 interviews (10 proposals).

Validity of the methods within the qualitative components was assessed by considering the attention researchers gave to issues such as reflexivity and negative cases. Validity was not addressed within proposals for more qualitative than quantitative components ($p = 0.001$), although any apparent difference in reports was not statistically significantly different ($p = 0.100$) (Table 4). Researchers did take the validity of qualitative methods seriously in some proposals, for example, paying attention to deviant cases and peer review of transcripts.

Concerns were identified with the sophistication of one-fifth of qualitative analyses. In nine studies the reported qualitative findings remained at a descriptive level, or reported findings in a quantitative manner only, or failed to distinguish between data collected using different methods such as focus groups and interviews.

## Integration

Integration of data or findings from the different methods received little attention in either proposals or reports, with researchers rarely discussing the type of integration, how it occurred in the context of team working and who was involved in it (Table 5). Because of the lack of integration, questions about the appropriateness of integration and the effect of integration on the rigour of individual methods were irrelevant.

## Inferences

In the reports, researchers were clear about which results had emerged from which methods, and inferences seemed appropriate (Table 6). For one-fifth of studies there was a concern that the inferences were based disproportionately on one method rather than the findings of all the methods. The imbalance was as likely to be towards qualitative findings as it was towards quantitative findings.

# Discussion

## The quality of studies in HSR

Mixed methods studies tend to be successful in HSR insofar that the qualitative and quantitative components are usually completed as planned. The main quality issue identified was a lack of transparency of the

**Table 5** Assessment of integration in mixed methods studies in HSR

| | Proposal (*n* = 45) | | | | Report (*n* = 47) | | | |
|---|---|---|---|---|---|---|---|---|
| | Yes | Yes, but | No | NEI or N/A | Yes | Yes, but | No | NEI or N/A |
| 1 Is the type of integration stated? | 11% | 0% | 84% | 4% | 2% | 2% | 94% | 2% |
| 2 Is the type of integration appropriate to the design? | 16% | 0% | 0% | 84% | 34% | 0% | 2% | 64% |
| 3 Has enough time been allocated for integration? | 2% | 0% | 13% | 85% | | | | |
| 4 Is the approach to integration detailed in terms of working together as a team? | 7% | 0% | 80% | 13% | | | | |
| 5 Does the dissemination strategy detail how the mixed methods will be reported in final reports and peer-reviewed publications? | 0% | 0% | 84% | 16% | | | | |
| 6 Are the personnel who participate in the integration clearly identified? | 9% | 0% | 80% | 11% | 6% | 0% | 70% | 23% |
| 7 Did appropriate members of the team participate in integration? | | | | | 0% | 0% | 2% | 98% |
| 8 Is there evidence of communication within the team? | | | | | 19% | 0% | 6% | 75% |
| 9 Has rigour been compromised by the process of integration? | | | | | 4% | 0% | 0% | 96% |

NEI, not enough information; N/A, not applicable

For peer review only


For peer review only - http://bmjopen.bmj.com/site/about/guidelines.xhtml

**Table 6** Assessment of the inferences made in completed reports of mixed methods studies in HSR

| | Report ($n$ = 47) | | | |
|---|---|---|---|---|
| | Yes | Yes, but | No | NEI or N/A |
| 1 Is there clarity about which results have emerged from which methods? | 87% | 2% | 6% | 4% |
| 2 Are inferences appropriate? | 83% | 4% | 9% | 4% |
| 3 Are the results of all the methods considered sufficiently in the interpretation? | 66% | 6% | 19% | 9% |

NEI, not enough information; N/A, not applicable

mixed methods aspects of the studies and the individual components. The qualitative components were more likely to be poorly described than the quantitative ones. To some extent the poor description of qualitative methods is not a surprising finding given the historical dominance of quantitative methods in HSR. However, it raises concerns that the HSR community may be failing on occasions to exploit the potential of qualitative methods within mixed methods studies. Where a qualitative component is in a supporting role to a more dominant method, and does not have stand-alone status in terms of independently addressing an aspect of the research question, then limited description is acceptable. However, because researchers were often not explicit about the status of methods within the study design, it was difficult to make judgements about the individual components in the context of the design used. Integration of data and findings is a key part of mixed methods research. There was no evidence that inappropriate integration was undertaken because there was a tendency for researchers to keep the qualitative and quantitative components separate rather than attempt to integrate data or findings in reports or publications.[12]

## Developing quality criteria for mixed methods studies in HSR

There was a lack of transparency in the reporting of mixed methods studies in HSR which made it difficult to assess other aspects of the quality of these studies. This has been identified as a problem facing the quality assessment of other types of studies[11] and has led to the development of guidelines for reporting studies. Creswell has suggested a list of issues to consider when designing a mixed methods study[10] and we have considered this in conjunction with the literature on the quality of mixed methods studies to suggest some guidelines for Good Reporting of A Mixed Methods Study (GRAMMS) (Box 1). We present this as guidance for researchers rather than as a formal checklist.

**Box 1** Good Reporting of A Mixed Methods Study (GRAMMS)

(1) Describe the justification for using a mixed methods approach to the research question
(2) Describe the design in terms of the purpose, priority and sequence of methods
(3) Describe each method in terms of sampling, data collection and analysis
(4) Describe where integration has occurred, how it has occurred and who has participated in it
(5) Describe any limitation of one method associated with the present of the other method
(6) Describe any insights gained from mixing or integrating methods

## Limitations

The study is based on mixed methods research funded by one commissioner in one country. The response rate to requests for documentation for mixed methods studies was good but non-responders may have been more likely to be problematic studies, biasing the findings towards higher quality studies. The questions were devised and applied by one researcher (AOC) in the context of team discussions which meant that the data extraction process was unchallenged by an external source. A coding protocol was devised to accompany the data extraction form to aid transparency and reduce intra-rater variability. However the studies could have been rated differently by another researcher. Finally, the studies included were funded between 1994 and 2004 and improvements may have occurred since then.

We have taken a technical stance in our discussions of quality in mixed methods research. However, the philosophical stance adopted by researchers may affect the quality criteria they use, and wish to see applied to their studies. Subtle realism[30] has been proposed as a philosophical position appropriate for qualitative and quantitative research in health technology assessment.[21] An implication of this stance is that researchers would need to consider whether reflexivity has been applied to the whole of a mixed methods study rather than simply the qualitative component.

## Conclusions

This is the first attempt to consider the quality of mixed methods studies in HSR. We are not offering this as a definitive approach to be used by others, but to start the debate about how to assess and improve quality. We recommend that if we use mixed methods studies in HSR then we need to be more transparent about the design and the individual components in the context of the design, and attempt to integrate data and findings from the qualitative and quantitative methods.

## Acknowledgements

Many thanks to the researchers who kindly sent copies of their study documents. The Medical Research Council funded the study through their Fellowship scheme.

For peer review only


For peer review only - http://bmjopen.bmj.com/site/about/guidelines.xhtml

## References

1 O'Cathain A, Murphy E, Nicholl J. Why, and how, mixed methods research is undertaken in health services research: a mixed methods study. *BMC Health Serv Res* 2007;**7**:85
2 Bryman A. Quantitative and qualitative research: further reflections on their integration. In: Brannen J, ed. *Mixing Methods: Qualitative and Quantitative Research*. Aldershot: Ashgate, 1992:57–78
3 Caracelli VJ, Riggin LJC. Mixed-method evaluation: developing quality criteria through concept mapping. *Eval Pract* 1994;**15**:139–52
4 Creswell JW, Fetters MD, Ivankova NV. Designing a mixed methods study in primary care. *Ann Fam Med* 2004;**2**:7–12
5 Datta L. A pragmatic basis for mixed-method designs. In: Greene JC, Caracelli VJ, eds. *Advances in Mixed-method Evaluation: The Challenges and Benefits of Integrating Diverse Paradigms*. San Francisco, CA: Jossey-Bass, 1997:33–46
6 Teddlie C, Tashakkori A. Major issues and controversies in the use of mixed methods in the social and behavioural sciences. In: Tashakkori A, Teddlie C, eds. *Handbook of Mixed Methods in Social and Behavioural Research*. London: Sage Publications, 2003:3–50
7 Sale JEM, Brazil K. A strategy to identify critical appraisal criteria for primary mixed method studies. *Quality and Quantity* 2004;**38**:351–65
8 Creswell JW, Plano-Clark V. *Designing and Conducting Mixed Methods Research*. Thousand Oaks, CA: Sage Publications, 2007
9 Brannen J. Combining qualitative and quantitative approaches: an overview. In: Brannen J, ed. *Mixing Methods: Qualitative and Quantitative Research*. Aldershot: Ashgate, 1992:3–38
10 Creswell JW. *Research Design. Qualitative, Quantitative, and Mixed Methods Approaches*. 2nd edn. London: Sage Publications, 2003
11 Mays N, Pope C, Popay J. Systematically reviewing qualitative and quantitative evidence to inform management and policy-making in the health field. *J Health Serv Res Policy* 2005;**10** (Suppl. 1):6–20
12 O'Cathain A, Murphy E, Nicholl J. Integration and publications as indicators of 'yield' from mixed methods studies. *Journal of Mixed Methods Research* 2007;**1**:147–63
13 Brewer J, Hunter A. *Multimethod Research*. London: Sage Publications, 1989
14 Tashakkori A, Teddlie C, eds. *Handbook of Mixed Methods in Social and Behavioural Research*. London: Sage, 2003
15 Tashakkori A, Teddlie C. *Mixed Methodology: Combining Qualitative and Quantitative Approaches*. London: Sage, 1998
16 Gorard S, Taylor C. *Combining Methods in Educational and Social Research*. Maidenhead: Open University Press, 2004
17 Brannen J, ed. *Mixing Methods: Qualitative and Quantitative Research*. Aldershot: Ashgate, 1992
18 Bryman A. *Quantity and Quality in Social Research*. London: Routledge, 1988
19 Greene JC, Caracelli VJ, eds. *Advances in Mixed-method Evaluation: The Challenges and Benefits of Integrating Diverse Paradigms*. San Francisco, CA: Jossey-Bass, 1997
20 Fielding NG, Fielding JL. *Linking Data*. Sage Publications, 1986
21 Murphy E, Dingwall R, Greatbatch D, Parker S, Watson P. Qualitative research methods in health technology assessment: a review of the literature. *Health Tech Assess* 1998;**2**:16
22 Mason J. Linking qualitative and quantitative data analysis. In: Bryman A, Burgess RG, eds. *Analysing Qualitative Data*. London: Routledge, 1994:89–110
23 Miller S. Impact of mixed methods and design on inference quality. In: Tashakkori A, Teddlie C, eds. *Handbook of Mixed Methods in Social and Behavioural Research*. London: Sage Publications, 2003:423–55
24 Katrak P, Bialocerkowski AE, Massy-Westropp N, Kumar VS, Grimmer KA. A systematic review of the content of critical appraisal tools. *BMC Med Res Meth* 2004;**4**:22
25 Silverman D. *Doing Qualitative Research. A Practical Handbook*. London: Sage Publications, 2000
26 Steckler A, Mcleroy KR, Goodman RM, Bird ST, McCormick L. Toward integrating qualitative and quantitative methods: an introduction. *Health Educ Q* 1992;**19**:1–8
27 Chen H. Applying mixed methods: a dominant methodology for the future? In: Greene JC, Caracelli VJ, eds. *Advances in Mixed-method Evaluation: The Challenges and Benefits of Integrating Diverse Paradigms*. San Francisco, CA: Jossey-Bass, 1997:61–72
28 Sandelowski M. Combining qualitative and quantitative sampling, data collection, and analysis techniques in mixed-method studies. *Res Nurs Health* 2000;**23**:246–55
29 O'Cathain A, Thomas KJ. "Any other comments?" Open questions on questionnaires – a bane or a bonus to research? *BMC Med Res Meth* 2004;**4**:25
30 Hammersley M. *What's Wrong with Ethnography?* London: Routledge, 1992

For peer review only


For peer review only - http://bmjopen.bmj.com/site/about/guidelines.xhtml

BMJ Open

# Implementation of national antenatal hypertension guidelines: a multi-centre mixed methods study

| | |
|---|---|
| Journal: | *BMJ Open* |
| Manuscript ID | bmjopen-2019-035762.R1 |
| Article Type: | Original research |
| Date Submitted by the Author: | 30-Jun-2020 |
| Complete List of Authors: | Whybrow, rebecca; King's College London, Women and Children's Health; Guy's and Saint Thomas' Hospitals NHS Trust, Division of Women and Children's Health<br>Webster, Louise; King's College London, Women and Children's Health<br>Girling, Joanna; Chelsea and Westminster Hospital NHS Foundation Trust<br>Brown, Heather; Brighton and Sussex University Hospitals NHS Trust<br>Wilson, Hannah; King’s College London, Women and Children's Health<br>Sandall, Jane; Kings College, London, Women and Children's Health<br>Chappell, Dr Lucy; King’s College London, Department of Women and Children’s Health; Guy's and Saint Thomas' Hospitals NHS Trust, Division of Women and Children's Health |
| <b>Primary Subject Heading</b>: | Obstetrics and gynaecology |
| Secondary Subject Heading: | Cardiovascular medicine |
| Keywords: | Maternal medicine < OBSTETRICS, OBSTETRICS, Hypertension < CARDIOLOGY |
| | |

SCHOLARONE™
Manuscripts

*I, the Submitting Author has the right to grant and does grant on behalf of all authors of the Work (as defined in the below author licence), an exclusive licence and/or a non-exclusive licence for contributions from authors who are: i) UK Crown employees; ii) where BMJ has agreed a CC-BY licence shall apply, and/or iii) in accordance with the terms applicable for US Federal Government officers or employees acting as part of their official duties; on a worldwide, perpetual, irrevocable, royalty-free basis to BMJ Publishing Group Ltd ("BMJ") its licensees and where the relevant Journal is co-owned by BMJ to the co-owners of the Journal, to publish the Work in this journal and any other BMJ products and to exploit all rights, as set out in our licence.*

*The Submitting Author accepts and understands that any supply made under these terms is made by BMJ to the Submitting Author unless you are acting as an employee on behalf of your employer or a postgraduate student of an affiliated institution which is paying any applicable article publishing charge ("APC") for Open Access articles. Where the Submitting Author wishes to make the Work available on an Open Access basis (and intends to pay the relevant APC), the terms of reuse of such Open Access shall be governed by a Creative Commons licence – details of these licences and which Creative Commons licence will apply to this Work are set out in our licence referred to above.*

*Other than as permitted in any relevant BMJ Author's Self Archiving Policies, I confirm this Work has not been accepted for publication elsewhere, is not being considered for publication elsewhere and does not duplicate material already published. I confirm all authors consent to publication of this Work and authorise the granting of this licence.*

For peer review only

**Title:** Implementation of national antenatal hypertension guidelines: a multi-centre mixed methods study

Rebecca Whybrow[1], Louise Webster[1], Joanna Girling[2], Heather Brown[3], Hannah Wilson[1], Jane Sandall[1], Lucy Chappell[1]

1 Department of Women and Children's Health, King's College London, St Thomas' Hospital, London, UK.

2 Chelsea and Westminster Hospital NHS Foundation Trust, London, UK.

3 Brighton and Sussex Universities Hospital Trust, East Sussex, UK.

Corresponding author: Rebecca Whybrow, Department of Women and Children's Health, King's College London, St Thomas' Hospital, London, UK. Rebecca.whybrow@kcl.ac.uk Tel: 07804690276

**Word Count:** 4796

**Abstract:**

**Objective** To evaluate the implementation of NICE antenatal hypertension in pregnancy guidelines, to identify strategies to reduce incidences of severe hypertension and associated maternal and perinatal morbidity and mortality in pregnant women with chronic hypertension.

**Methods** We used a multi-method multi-site approach to establish implementation of guidelines and the associated barriers and facilitators. We used a national survey of healthcare professionals (n=97), case-notes review (n=55) and structured observations (n=42) to assess implementation. The barriers and facilitators to implementation were identified from semi-structured qualitative interviews with healthcare professionals (n=13) and pregnant women (n=18) using inductive thematic analysis. The findings were integrated and evaluated using the Consolidated Framework for Implementation Research (CFIR).

**Setting and participants** Pregnant women with chronic hypertension and their principle carers (obstetricians, midwives, and physicians), at three NHS hospital trusts with different models of care.

**Results** We found severe hypertension to be prevalent (46% of case-notes reviewed) and target blood pressure practices to be sub-optimal (56% of women had an antenatal blood pressure target documented). Women were infrequently given information (52%) or offered choice (19%) regarding antihypertensives. Women (14/18) reported internal conflict in taking antihypertensives and non-adherence was prevalent (8/18). Women who were concordant with treatment recommendations described having mutual trust with professionals mediated through appropriate information, side-effect management and involvement in decision-making. Professionals reported needing updates and

For peer review only

tools for target blood pressure setting and shared decision-making underpinned by antihypertensive safety and effectiveness research.

**Conclusions** Women's nonadherence to antihypertensives is higher than anticipated. Sub-optional information provision around treatment, choice of antihypertensives and target setting practices by healthcare professionals may be contributary. Understanding the reasons for non-adherence will inform education and decision-making strategies needed to address both clinician and women's behaviour. Further research into the effectiveness and long-term safety of common antihypertensives is also required.

**Strengths and limitations of this study**

- Multi-methodological approaches and an implementation framework improved the reliability, validity, and generalisability of the study.
- Structured observations were carried out using a validated tool with high interrater reliability.
- Women's medication behaviours were explored in-depth using a qualitative interview approach and have identified antihypertensive side-effects to be a factor of non-adherence in pregnant women.
- About two-fifths of women who participated in this study were from Black, Asian and minority ethnic groups, providing a diverse range of voices.
- Respondents to the survey were self-selecting and may represent a relatively interested group of healthcare professionals.

For peer review only

**BACKGROUND**

Hypertension in pregnancy is one of the leading causes of maternal mortality worldwide[1] and although mortality is declining in the UK,[2] women can still experience substantial morbidity from complications such as eclampsia and stroke.[3] Additionally, perinatal mortality remains high, with the UK population-attributable risk of stillbirth from chronic hypertension at 14%[4] and around half of all neonates born to mothers who have had severe hypertension in pregnancy being admitted to the neonatal unit.[5] The morbidity and mortality attributable to hypertension, in many cases, may be modifiable through optimal use of antihypertensive agents during pregnancy.

The National Institute for Health and Care Excellence (NICE) hypertension in pregnancy guidelines (2010)[6] and linked quality statements (2013)[7] contain a quality statement regarding the provision of information on the use of safe antihypertensive medication in pregnancy and has related guidance that recommends discontinuation of teratogenic medications such as angiotensin-converting-enzyme inhibitors or angiotensin II receptor blockers with prescribing of safe alternatives. Any prescribing of alternative antihypertensive medication should be dependent on pre-pregnancy treatment, side-effect profiles and teratogenicity. A second quality statement advocates that women taking antihypertensive medication should have a blood pressure target (usually of less than 150/100mmHg) set in pregnancy. All NICE guidelines are underpinned by the recommendation of enabling patients to actively participate in their care which includes adopting a shared decision-making approach to treatment decisions.[8]

Despite publication of the guideline almost a decade ago, the implementation and evaluation of associated determinants of uptake have not been nationally evaluated. As a result, targeted strategies to reduce maternal and perinatal morbidity (and mortality) resulting from severe hypertension remain unidentified. Using the Consolidated Framework for Implementation Research (CFIR), [9] the aim of the study was to evaluate the implementation of NICE hypertension in pregnancy guidelines, to identify strategies to reduce incidence of severe hypertension and associated maternal and perinatal morbidity and mortality in pregnant women with chronic hypertension. In many countries, there is a movement toward establishing consensus-driven standardised clinical guidelines with the aim of improving patient safety and clinical outcomes. Whilst new research continually emerges, guidelines are periodically updated and therefore remain an appropriate standard for evaluating routine clinical practice.[10]

For peer review only

## RESEARCH DESIGN AND METHODS

### Study setting and overall methodology

The CHAMPION study (Chronic Hypertension in pregnAncy iMPlementatION study) is a multi-methods evaluation of the implementation of the NICE hypertension in pregnancy guidelines (2010 and updated in 2013) in women with chronic hypertension diagnosed before 20 weeks.[6 7] Ethical approval for the CHAMPION study was provided by the National Research Ethics Service (17/LO/2041). The study aimed to evaluate the variability in implementation of hypertension management practices set out in the NICE hypertension in pregnancy guidelines (2010).[6] As all guidelines should be underpinned by the 'Patient experience in adult NHS services guideline'[8] which specifies 'actively involving patient in decisions about their care through 'information provision' and 'shared decision-making' the provision of information and women's involvement in decision-making was also evaluated. The involvement of women in decision-making was considered integral to the implementation study because successful hypertension management strategies involve the adherence to, alongside the prescribing of, antihypertensive medication.

Implementation was assessed through multiple methods: an online national survey of healthcare professionals, designed to describe general trends in guideline implementation; through review of the maternity case-notes of women who had already given birth, a method that assessed the documentation of hypertension management occurrence in each woman's maternity record. Aspects of care that would not normally be documented or are more difficult to capture, such as in-consultation discussions and occurrence of shared decision-making were assessed through observations carried out by a midwife researcher (RW). The evaluation of the barriers and facilitators to implementation of NICE guidelines was assessed through qualitative interviews (with the same women and healthcare professionals who participated in the observation phase) using the Consolidated Framework for Implementation Research (CFIR). The study draws on CFIR as a theoretical framework to guide data collection, analysis, and interpretation. The CFIR framework specifically evaluates five key domains that influence implementation; each domain has several subgroups to it, although only those relevant to this study have been identified. These include the intervention characteristics (the NICE guidelines), the outer context (the pregnant women), the inner context (NHS maternity services), individual context (the healthcare professionals) and the process of implementation (potential strategies).

Implementation of guidelines was assessed between November 2017 to December 2018 at three NHS Trusts with typical configurations of services for pregnant women with hypertension in the UK. Hospital Trust 1 was a tertiary city centre hospital with a newly formed specialist service that included

For peer review only

consultant obstetricians, obstetric physicians and midwives who provided antenatal and intrapartum care to women with chronic hypertension within a specialist clinic; Hospital Trust 2 was a suburban district general hospital with a consultant-led antenatal clinic with antenatal midwives alongside providing care to women with a variety of pre-existing medical conditions; and Hospital Trust 3 had both a tertiary and a semi-rural hospital with a joint obstetric and physician led clinic and usual community-based midwifery care. No adjustment for clustering was required as no statistical comparison between sites was made. The NICE hypertension in pregnancy guidelines (2010)[6] had been adopted into local clinical guidelines at all three participating NHS Trusts for several years prior to the assessment of implementation.

**The National Survey**

The implementation of evidence-based practices for the management of hypertension in pregnancy was assessed through self-reporting using an online survey (surveygizmo/s3). We embedded questions relating to the uptake of the NICE hypertension in pregnancy guidelines (2010)[6] using the TIDieR framework.[11] The 12-item TIDieR checklist (brief name, why, what (materials), what (procedure), who provided, how, where, when and how much, tailoring, modifications, how well (planned), how well (actual) is an extension of the CONSORT 2010 statement (item 5) and the SPIRIT 2013 statement (item 11). Although the emphasis of the TIDieR checklist is on reporting interventions for trials, the checklist was used as a basis for this survey (but not as a reporting guideline) as it is also intended to apply across all evaluative study designs.[11] There is no single database of healthcare professionals' email addresses so national organisations including British Maternal and Fetal Medicine Society (BMFMS), Macdonald UK Obstetric Medicine Society (MOMS) and Royal College of Midwives (RCM) were asked to email the survey (April to September 2018) to their members. No fee was charged as members' contact details were not shared with us and as a result the response rate could not be calculated. Ninety-seven healthcare professionals from sixty-nine NHS Trusts was obtained, including 53 consultant obstetricians (55%), 16 doctors in training (16%), 22 specialist midwives (23%) and six community midwives (6%) (full copy of survey questions shown in supplementary material 1).

**Case-notes review**

The implementation of NICE guidelines (2010)[6] was also assessed through review of 100 maternity case-notes of women with chronic hypertension identified from the electronic maternity records (32, 33, 35 women per Trust). At two of the Trusts all women who had given birth in 2017 were included, whereas at the other Trust all women who had given birth over the final three months of 2017 were

For peer review only

included as this third Trust had approximately four times the number of women with chronic hypertension per annuum. In the UK, many women have abridged electronic maternity records and extensive handheld paper notes that are carried throughout pregnancy but are stored thereafter in the hospital. Both the electronic system and paper notes were obtained in the case-notes review of care. Due to use of varying terms for hypertension on the electronic system, some women identified for case-note review were excluded as they did not have chronic hypertension when the full case-notes were examined. Other reasons for exclusion included early miscarriage and transfer of care to another maternity unit. Data extraction based on the NICE hypertension in pregnancy guidelines (2010)[6] was completed by two midwife researchers (RW, HW), and minor discrepancies were resolved by discussion between the two researchers. It was not necessary to include a third reviewer as no major discrepancies were identified. Unclear or absent documentation including height, weight and body mass index or antenatal blood pressure recordings was recorded as missing data. Severe hypertension was defined as systolic blood pressure greater than or equal to 160 mmHg systolic or diastolic blood pressure greater than or equal to 110 mmHg. For the assessment of BP targets, the quality statement related to documentation of a target (or not), not to the specific numerical thresholds chosen.

**Observations**

Forty-two antenatal appointments involving 23 women with chronic hypertension and their respective doctors (nine) and midwives (five) were observed by a midwife researcher (RW) at the three NHS Trusts. Women with chronic hypertension were purposively sampled at their first obstetric antenatal appointment and based on the availability of the midwife researcher, were approached consecutively along with their respective healthcare professionals until data saturation occurred. Staff and women gave written informed consent. Two women declined recruitment to the study. During observations, data about antenatal care provision were recorded using the Calgary-Cambridge communication guide[12] chosen for validity in relation to the research question, and its high interrater reliability. For example, offering choice is a sub-section of shared decision-making and is defined as "encourages patient to make choices and decisions to the level that they wish". Attainment of each section and sub-sections was established through the analysis of all 42 appointments using descriptive statistics.

**Semi-structured interviews**

Views about barriers and facilitators to implementation of evidence-based guidelines were collected from nine doctors and four midwives who were providing antenatal care for women with chronic hypertension. The interviews were carried out by a midwife researcher (RW) following informed consent and took place in privacy away from the clinical setting. The interviews were audio

For peer review only

transcribed, coded and thematically analysed using inductive reasoning.[13] The codes generated formed small themes which were organised into the CFIR evaluation guide.[14] As formal implementation strategies had not been adopted beyond producing local guidance, interviewees were asked how they thought they could improve the implementation in the future.

Semi-structured interviews with 18 women recruited for antenatal observations were carried out in the third trimester with informed consent. Women were asked about their antenatal care experiences using an interview schedule which reflected the concepts from the International Consortium for Health Outcome Measure (ICHOM) maternity standards sets[15] which include women's overall satisfaction with their care during pregnancy; satisfaction with information provision and their relationships with their care providers (see supplementary material 2). ICHOM standards are internationally recognised measures that evaluate health outcomes that are important to patients (or pregnant women) and are used to improve local healthcare and compare outcomes internationally. The closed survey questions were turned into open ended questions to explore in-depth the quality of antenatal care provided. The interviews were carried out by a midwife researcher (RW) and took place away from the clinical setting, with assurance that discussions would not be shared with healthcare professionals and that participation or non-participation would not influence their care. The interviews were audio transcribed, coded, and thematically analysed using an inductive approach. Women's experiences were analysed to improve understanding of their antenatal care needs, which included how their hypertension was managed and the barriers and facilitators to the uptake of antihypertensives in pregnancy.

**Data analysis**

The quantitative and qualitative data were analysed separately before being integrated. Descriptive analysis and summary statistics were used for the quantitative data. The semi-structured interviews were thematically analysed by researchers (RW, JS and LC) using inductive techniques and typically lasted between 30 and 60 minutes.[16] The mixed-methods data were integrated and analysed using the CFIR evaluation framework.[14] This included probing the inductively generated qualitative themes that related to implementation. The interpretation of the intervention constructs (characteristics, the inner and outer settings, the individual characteristics and the implementation processes) was carried out initially by the midwife researcher (RW) who collected the data, then with a second and third researcher (LC, JS) interpreting and discussing final interpretation of integrated data. Rigour was maintained through member reflection, attention to interview and transcription quality and systematic analysis. Rigour was improved using multiple data sources, a comprehensive integration framework (CFIR) and a mixed methods integration checklist.[17] Researchers were aware of, and

For peer review only

sensitive to, the way in which their roles as midwives and doctor may have shaped the generation and analysis of the qualitative data.

**Patient and Public Involvement**

A patient participant involvement (PPI) group consisting of women with experience of hypertension in pregnancy (n=7) and a maternity voices partnership group (n=15) provided feedback on the design of the study, research questions and outcome measures. The views of Black, Asian and minority ethnic women were purposively sought as they are disproportionately represented in the chronic hypertension in pregnancy population. PPI focus groups discussed what aspects of care were important to evaluate, this included the information women were given during pregnancy and whether women were involved in decision about their care. They also provided constructively critical feedback on the patient information leaflets and consent forms.

**RESULTS**

Antenatal care for women with chronic hypertension was provided by consultant obstetricians and midwives at all three hospitals. In two of the hospitals, women with chronic hypertension had designated midwives attached to the obstetric clinic. Approximately one-third of those recruited to the study had a BMI over 30kg/m$^2$, approximately one-third were over the age of 35 and approximately two-fifths were of Black, Asian and minority ethnic backgrounds (shown in supplementary material 3). Hospital Trust 1 had four times the population of women with chronic hypertension compared to the other two units, comprising a large black minority ethnic population (many with associated co-morbidities). Perinatal outcomes from the fifty-five pregnancies identified for case-notes review showed that just under half of the women (46%) developed severe hypertension and that one in six babies were admitted to the neonatal unit (16%) (shown in supplementary material 4). At all three hospitals medical history of women with chronic hypertension was inaccurate in the maternity records system and episodes of severe hypertension were recorded only in hand-written notes.

**Implementation of NICE hypertension in pregnancy 2010 guidelines and 2013 quality standards**

Setting a blood pressure target (quality statement 3)

Both the survey and the case-notes review found the practice of setting an antenatal target blood pressure to be variable (table 1). Just over half of women with chronic hypertension had a target blood

For peer review only - http://bmjopen.bmj.com/site/about/guidelines.xhtml

pressure documented in maternity notes (44% did not) yet substantial variation in practice between hospitals existed. At Hospital Trust 1, 77% of women had a target blood pressure documented in pregnancy compared to 23% and 38% at Hospital Trusts 2 and 3 respectively (supplementary material 5). Whilst it is possible that undocumented discussions occurred during consultations, which could not be extracted from case-note review, such discussions would not be accessible on a longer term basis to the woman or to other healthcare professionals involved in her care. The survey results support the case-notes review findings as only a third of healthcare professional respondents reported always setting a target. The practice of undocumented 'unshared' target setting was identified through case-notes review. Evidence of blood pressure targets being used by healthcare professionals but not shared with the woman and other professionals ('unshared') was found in about three quarters of women whose blood pressure rose above systolic 150mmHg and or diastolic 100mmHg action was taken by professionals to lower it. Action was defined as making changes to blood pressure treatment, changing frequency of blood pressure monitoring or frequency of appointments (table 1).

Table 1. Variation in implementation of evidence-based care evaluated through a national survey of obstetricians and midwives and women's case-notes review at three representative NHS Trusts.

| **Care quality indicators** | **National Survey n=97 (%)** | **Case-notes review n=55 (%)** |
|---|---|---|
| **Blood pressure target setting (QS3)** | | |
| Target blood pressure 'always' set | 36 (37.1) | |
| Target blood pressure 'almost always' set | 36 (37.1) | |
| Target blood pressure 'never' set | 1 (1.0) | |
| Target blood pressure not applicable (midwife) | 24 (23.3) | |
| Target blood pressure set at first opportunity (whichever first: booking or commencement of AHT) | - | 9 (18.0) |
| Target blood pressure not documented | | 26 (43.6) |
| **Systolic target blood pressure** | | |
| <160mmHg | 8 (8.2) | |
| <150mmHg | 89 (91.8) | 2 (7.4) |
| ≤140mmHg | | 27 (49.0) |
| **Diastolic target blood pressure** | | |
| <100mmHg | 94 (96.9) | 2 (7.4) |
| ≤90mmHg | | 27 (49.0) |

For peer review only

| | | |
|---|---|---|
| Action taken to reduce blood pressure if above 150/100mmHg | | 13/17 (76.5) |
| **Safe antihypertensive prescribing (linked to QS1)** | | |
| **ACEi and ARBs cessation** | | |
| On ACEis or ARBs at antenatal booking appointment | | 4 (7.3) |
| Stopping ACEi or ARBs at first app if woman on either | | |
| Always | 57/86 (66.3) | - |
| Almost always | 27/86 (31.4) | - |
| ACEis or ARBs stopped at 1st obstetric appointment | | 4/4 (100.0) |
| **1st line AHT prescribing (non-exclusive)** | | |
| Labetalol | 85 (87.6) | 28 (50.9) |
| Nifedipine | 32 (33.0) | 9 (16.4) |
| Methyldopa | 29 (29.9) | 8 (14.5) |
| Other e.g. amlodipine | 2 (2.1) | 4 (7.3) |
| None | - | 6 (10.9) |
| **2nd line AHT prescribing (non-exclusive)** | | |
| Nifedipine | 79 (81.4) | 9 (16.4) |
| Methyldopa | 60 (61.9) | 4 (7.3) |
| Labetalol | 38 (39.2) | 3 (5.4) |
| Amlodipine | 37 (38.1) | 2 (3.6) |
| Doxazosin | 23 (23.7) | 0 (0.0) |
| Other | 5 (5.2) | 0 (0.0) |
| None | - | 37 (67.3) |

Antihypertensive information provision, decision-making and prescribing (quality statement 1 and associated guidance)

Variation in practice regarding first- and second-line prescribing was identified through both the notes review and survey (table 1). In both, labetalol was the most commonly prescribed first line and nifedipine the most commonly used second line antihypertensive agent; nevertheless, in about half of the case-notes reviewed labetalol was not the first line antihypertensive prescribed. First line prescribing is not always exclusive as it may vary by ethnicity (e.g. some doctors use labetalol as first line for many women, but nifedipine for Black women, in line with national guidelines for prescribing outside of pregnancy)[18] which may explain the variation in prescribing practice that existed

For peer review only

(supplementary material 5). Variation may also be explained by clinician preference or medication preference identified through shared decision-making.

Information provision about antihypertensive prescribing

Across all three Trusts, 52% (41/79) of the time the correct type and amount of information was provided during the consultation (measured using the Calgary-Cambridge Guide). Visual techniques such as drawing or using charts to provide information occurred during consultation in 14% (3/21) of cases.

Achieving a shared understanding: incorporating the woman's perspective

Of the survey respondents 96.9% strongly agreed or agreed that involving women with chronic hypertension in management plans during pregnancy was important. However, when asked to give examples of how they involve women, only 4.3% identified discussing risks and benefits of treatment choice and 10% of respondents identified that women could be involved in plans about antihypertensive prescribing. The observations in the three hospital trusts found that 43% of the time (41/96) shared decision-making occurred and 19% of women (3/16) were offered a choice regarding their hypertensive plans (including choice of antihypertensive).

**Barriers and Facilitators to implementation (CFIR)**

Intervention characteristics (evidence and guideline)

All professionals interviewed, except one, saw value in having national guidance and understood that the local guidelines had been adapted from the 2010 national guideline.[6] Midwives relied more on local guidelines compared to obstetricians who referred more commonly to NICE guidelines. Some of the medical professionals had been involved in the development of a NICE guideline and were aware of the strengths and limitations of producing evidence-based guidelines in terms of the need for timely updating. Professionals described difficulties in creating guidelines where there is a paucity of robust data as is sometimes the case in maternity care. Weak, out of date or absent evidence influenced doctors' decisions not to implement guidelines. Some doctors described the weaknesses in the evidence underpinning the hypertension guidelines and described relying more on recent research compared to older national guidelines (table 2). The professionals identified that further research is necessary to support evidenced-based national guidelines (figure 1).

For peer review only

Table 2. Barriers to healthcare professional's implementation of hypertension in pregnancy guidelines, based on Consolidated Framework for Implementation Research (CFIR) implementation themes.

| **CFIR implementation themes** | **Frequency** | **Codes** | **Representative answer** |
|---|---|---|---|
| **Intervention characteristics** | | | |
| Evidence strength, quality, source, and adaptability | 17 | AHT prescribing; target setting; | - "I think the fact that it says use labetalol first line is not what we do, I don't believe the evidence for labetalol being better than methyldopa is there."[H]<br>- "we can't get away from the fact that there aren't the source data there to make evidence-based guidelines."[B]<br>- So, I kept a close track of what was happening with the CHIPS study…I got a lot of information and knowledge from it."[A] |
| **Inner setting** | | | |
| Structural characteristics | 43 | Information provision; pathways and models; training and education; time | - "I don't think we have a hand-out for, to give to hypertensive women about hypertension in pregnancy"[L]<br>- "we don't have a dedicated hypertension clinic here. So, most of these women will get seen in general antenatal clinic"[I]<br>- "you have people coming in three times weekly or something for their blood pressure, really? And other people who perhaps aren't being seen enough"[I] |
| Relative priority | 26 | Guidelines; self-study; beliefs; experience; | - "Well actually I don't even know what the NICE guidelines are for hypertension, I'm not a… as my colleagues will tell you, not a huge fan of NICE, in many ways."[L]<br>- "I'm not just interested in guidelines; I'm interested in people's clinical experience…and that feel."[C] |

For peer review only

| | | | |
|---|---|---|---|
| Culture of decision-making | 19 | Patriarchy; shared decision-making; type of decision: emergency, urgent and non-urgent | - "Doctors… see it as patients not doing what they're told"[A]<br>- "I think that there's a balance to be had between involving women in the decisions, versus, them coming for expert recommendations"[F]<br>- "If I have a clinical situation where I want to start antihypertensives because she's got a dangerously high blood pressure, then that discussion is inevitably truncated." [B] |
| **Individual characteristic** | | | |
| Beliefs about the intervention | 35 | AHT medication; AHT safety and side-effects; target setting | - "National guidelines do not sanction any particular antihypertensive, or that the, the drug licenses do not sanction any particular antihypertensive"[B]<br>- "I think that might be something we're not quite as good at as we should be about defining a target for women….I suspect it's something we don't really document and clarify"[H] |
| Self-efficacy | 17 | Women's concordance/ desire for involvement/ first language | - "I think sometimes women don't necessarily want to make the decision"[D]<br>- "There's a lot of 'mumsnet'….and I would say they take a, that advice just as seriously as they do the advice that we give them here."[C] |
| **Process of implementation** | | | |
| Engaging people and process of implementation | 16 | Using guidelines; updates, toolkits, and information; shared decision-making | - "Awareness for people, if you're a busy jobbing healthcare practitioner, keeping up to date with each new area"[H]<br>- "Practical toolkits to help with that consultation"[B]<br>- Evidenced based information having it more readily available for patient"[D] |

For peer review only

| | | | |
|---|---|---|---|
| Opinion leaders; Champions; | 5 | Utilisation of opinion leaders/ champions in implementation | - "I find as a midwife sometimes you're a bit powerless, you know what the guidelines are, but depending on the doctor you're working with, tends to be the influencing factor on the decisions that are made… so it seems to be clinician-based guidelines sometimes, rather than the trust or national guidelines"[D1] |

[1] Letters [A-M] represent the healthcare professionals interviewed

For peer review only

Inner setting (organisation structure and culture)

The most frequently cited barriers to implementing high quality care for women with chronic hypertension were linked to the structure and organisation of antenatal care. Interviewees reported that a lack of consensus and guidance exists relating to models of care (such as whether specialist services would improve outcomes through better implementation) and pathways of care (such as frequency of blood pressure and medication reviews) (table 2). Evidence-based recommendations on models, and pathways of care, were identified as future facilitators to providing optimal antenatal care (figure 1). Whilst most healthcare professionals initially described the uptake of the guidelines as a clinical priority during the interviews, clinicians identified difficulty with keeping up with recommendations and using them alongside clinical judgement as barriers to implementation (table 2).

Healthcare professionals considered the absence of written information a barrier to the uptake of antihypertensives in women with hypertension (table 2). A degree of paternalism exists in relation to involving women in decisions about their care. In principle, clinicians would like to involve women in decision-making, yet they gave many examples of situations where they would exercise restraint in doing so (table 2). Education and tools to support shared decision-making were identified as facilitators to optimizing antenatal care for women with hypertension (figure 1).

Characteristics of individuals (beliefs, knowledge, and self-efficacy)

Interview analysis identified doctors' and midwives' knowledge and beliefs as the second most frequently cited barrier and facilitator to the implementation of hypertension management guidelines (table 2). There existed confusion about whether the guidelines sanction one antihypertensive medication over another for the management of chronic hypertension and if so, what evidence was used to support this. Likewise, confusion about blood pressure targets was described frequently as outcomes from a recent randomised controlled trial superseded the pre-dated national guidelines (table 2). Whilst midwives experienced less self-efficacy than the doctors, doctors still experienced difficulties in this area. They occasionally described the women's beliefs and views as a barrier to implementing the recommendations (table 2).

For peer review only

335 Outer setting (women's views and experiences)

The quality of antenatal care experience was affected by women's internal conflict. There was also a high degree of variability in medication adherence (defined as, a blanket term factoring the extent to which patients' drug dosing histories conform, or not, to their corresponding prescribed drug dosing regimen).[19] and concordance (defined as, an agreement after negotiation between a woman and a healthcare professional that respects the beliefs and wishes of the woman in determining whether, when, and how medicines are to be taken).[20] Analysis identified that women require quality information about antihypertensives and their side-effects, blood pressure ranges in pregnancy, as well as support to actively participate in decision-making.

*Internal Conflict*

The majority (14 of 18) of women experienced internal conflict relating to the management of their hypertension during pregnancy, defined as a state of uncertainty about the course of action to take often in relation to making choices involving risk or uncertainty of outcomes (8) (figure 2a). The causes of internal conflict were identified as a lack of information provision, poorly managed side-effects, women's personal beliefs and factors relating to the healthcare professional (table 3).

For peer review only

352 Table 3. Barriers to women's uptake of hypertension in pregnancy guidelines

| CFIR outer context themes - Women's internal conflict | Frequency | Codes | Representative answer |
|---|---|---|---|
| Information | 30 | Medication (choices, dose, effectiveness, safety, interactions); severity of HTN; effect of HTN on pregnancy | - "[I wanted to know] how safe it is, about the dosage, about the, taking the med-, this medication, about the side-effects and so and so and so, if they think any other option for me, or if this medication is not working, what will be the other option for me"[J]<br>- "He was, you still need to carry on with your ramipril. I know I can't take it. It says in the leaflet not to take once you've hit 6 weeks, you need to stop. So, he was like oh, and then he phoned here, and he said oh well just take what you took before"[H] |
| Side-effects | 21 | Maternal side-effects; fetal side-effects; Interactions ; allergies; choices | - "They gave me first three, twice a day, then I was so giddy where I couldn't, if I take, I had to sleep all day for two days…Then I complained, but they still say to still take tablet."[I]<br>- "I'm on 18 pills a day, I do worry a bit about how they kind of potentially interact with each other and affect the baby"[F] |

For peer review only

| | | | |
|---|---|---|---|
| Beliefs | 17 | Hypertension status; understanding HTN; effectiveness AHT; safety AHT | - "I felt like I had to justify why I wasn't taking my tablet, which to me didn't seem right, 'cause if it, if my blood pressure was normal, and I took a tablet, surely my blood pressure then would be low?"[Q]<br>- "cause everything I take my baby takes. So, it's like, what happens if my child comes out and then they're addicted to something, or they're high-strung because of something, or they're really moody and they're crying all the time because of the medicine I've had to take for the past 4 months"[L] |
| HCP factors | 17 | Continuity; listening to women; explaining regimes, mutual trust; communication | - "My issue has been where I've seen somebody who doesn't know the history, and typically they are a more junior doctor, and typically they are ticking a box and following a flow chart….the doctor said, you know, we're going to come to an agreement together but there was absolutely no discussion, she had no interest in what I had to say."[K] |
| External factors | 7 | Family and friends; internet; access to services | - "My dad had been on beta blockers, which is what labetalol is, when he was younger, and he found, he was very ill on them, so he gave me a really negative impression of them"[P2] |

[2] Letters [A-R] represent the pregnant women interviewed

For peer review only

*Concordance*

All women identified as concordant with healthcare professional management plans described being adherent to their antihypertensives. Facilitators to concordance included trust in the healthcare professional, mediated through information about safety of antihypertensives in pregnancy, knowledge about target blood pressure in pregnancy hypertension, acknowledgement of medication side-effects and a positive interaction with the healthcare professional (including communication and approach to decision-making) (figure 2b).

*Adherence*

Internal conflict was an important determinant of non-adherence (figure 2a) as only the women who expressed internal conflict reported non-adherence to antihypertensive medication. Around half (8 of 18) the women interviewed described non-adherence to prescribed antihypertensives at some point during pregnancy with three women non-adherent at the time of interview (third trimester). However, nine of 14 women describing internal conflict were adherent at the time of interview which was mediated by the 'responsibility of motherhood' rather than concordance with the hypertension management plan (figure 2b).

Process of implementation (implementation strategies)

All three Trusts had a consultant obstetrician who led the care of women with chronic hypertension and could be considered the opinion leader. Two of three Trusts had a named midwife or team of midwives who specialised in the care of these women and were potential champions. However, influencers and champions were not always utilised to support guideline implementation. Further, as implementation of the guidelines had not been audited in any of the Trusts, although some outcome data was routinely collected and analysed, opportunities to address unwanted variability were being missed. These findings are supported by the national survey which found only a quarter of the Trusts collected and analysed the outcomes of women with chronic hypertension in pregnancy.

For peer review only

380

381 **DISCUSSION**

382 Women in this study (14/18) reported conflict relating to the uptake of prescribed antihypertensives
383 in pregnancy and in many cases (8/14) internal conflict resulted in non-adherence. The most
384 commonly cited reasons for conflict were lack of information provision, the side-effects experienced
385 from the medication, beliefs about safety of medication and uncertainty about normal blood pressure
386 ranges in pregnancy. Adherence to antihypertensives in conflicted pregnant women was mediated
387 through a responsibility to motherhood rather than through a trusting partnership with healthcare
388 professionals (supported by information provision, management of side-effects and relational factors)
389 as found in concordant adherent women. Despite this, our findings demonstrated that optimal
390 information provision about antihypertensives and shared decision-making occurred infrequently
391 during antenatal consultations. Our findings also illustrated that the implementation of blood pressure
392 target setting was sub-optimal as a result of 'unshared' or undocumented target setting and in some
393 cases an absence of target setting.

394 A major strength of the study is the recruitment of Black, Asian and minority ethnic women to both
395 the research (40%) and in the PPI planning stage as these women are disproportionally represented
396 in the chronic hypertension in pregnancy population. A further strength is the use of multi-
397 methodological approaches and an implementation framework in order to improve reliability, validity
398 and generalisability. However, results from the national survey may overstate compliance with
399 national guidance. The survey was sent out to healthcare professionals from professional
400 organisations; respondents were therefore self-selecting and may represent a relatively interested
401 group of healthcare professionals. The non-response rate is also unknown. The structured
402 observations were carried out using a validated tool with high interrater reliability.[12] However, the
403 observations were carried out by one midwife researcher which may affect the validity of the findings.
404 Finally, the purposive sampling of healthcare professionals providing routine antenatal care for
405 women with chronic hypertension resulted in a focus on lead carers (consultant obstetricians,
406 obstetric medicine specialists and named midwives) being interviewed, rather than doctors in training
407 and midwives in acute areas such as the maternity assessment unit.

408 The emergence of implementation science in recent years has identified that a gap between research
409 findings and clinical practice exists, and that clinical guideline production does not ensure evidence-
410 based practices are routinely adopted.[21] A recent study in British Colombia evaluated the
411 implementation of recently published pregnancy hypertension guidelines and its associated effect on
412 maternal and perinatal outcomes.[22] Following guideline dissemination the study reported a fall of

For peer review only - http://bmjopen.bmj.com/site/about/guidelines.xhtml

about a third in combined adverse maternal health outcomes (3.1% to 1.9%) but did not report a significant reduction in adverse perinatal outcomes.[22] However, the wanted and unwanted variability in guidance uptake was not reported and the underlying mechanisms that influenced outcomes is not described. Our study uses an implementation framework by which variability in the implementation of existing guidelines could be described and mechanisms that support and hinder their uptake can be analysed, uniquely identifying strategies to improve the uptake of guidance and reduce maternal and fetal morbidity. Critically, although the NICE hypertension in pregnancy guidelines[6] have been recently updated, the core hypertension management recommendations remain unchanged, as do the quality statements. Therefore, the findings of this study remain important and relevant to those wanting to improve implementation.

The study also adds to the small body of antihypertensive adherence in pregnancy research that has found antihypertensive side-effects are a determinant of non-adherence. One recent randomised controlled trial identified 11% of those included in randomisation discontinued the antihypertensive due to side-effects.[23] Through the qualitative interview approach that enabled in depth exploration of women's medication behaviours, our study found about 40% of all women did not adhere to their prescribed antihypertensives at some point during pregnancy. This number compared more similarly to an internet-based study of 210 pregnant women undertaken in Europe, America and Australia which identified a 32.9% non-adherence rate in women taking cardiovascular medications in pregnancy.[24] These findings are supported by similar smaller questionnaire-based studies of pregnant women's medication adherence. [25 26] Our study may have identified higher rates of non-adherence due to the nature of qualitative interviewing that explore in-depth women's experiences and therefore unpick medication behaviours in a way that quantitative studies cannot.

Women's adherence to antihypertensives in pregnancy was found to be sub-optimal, and strategies to improve adherence are likely to reduce incidences of severe hypertension and prevent associated morbidity (and mortality). [27] These include improved information provision about anti-hypertensives and blood pressure targets as well as embedding shared decision-making into practice. Improvements in target blood pressure setting practices overall are also likely to reduce incidences of severe hypertension and prevent associated morbidity (and mortality). [3 5]

This study adds to the body of research that already exists outside of pregnancy which demonstrates that implementation of guidelines is not optimally achieved through the process of diffusion.[21] Although there was some evidence that some aspects of implementation were improved by having a specialist service for hypertension, this is likely to be most easily justified in areas where there is a high prevalence of chronic hypertension. Therefore, strategies to improve implementation in wider

For peer review only

settings are required. Professionals require guideline updates, implementation toolkits (to improve target blood pressure setting practices, standardised information about antihypertensives and in consultation aids) as well as support to have better conversations with their patients about medication choices and to improve the involvement of the women in the decision-making. Professionals also need to buy into the evidence that underpins the guidance. Maternal and perinatal outcomes, which includes episodes of severe hypertension, should be collected annually, and used to support informed discussions about optimising antenatal care for this group of women.

Further research into the effectiveness and long-term safety of common antihypertensives in pregnancy and breastfeeding to support evidenced-based guidelines is required.[28] Future research may also wish to evaluate strategies to reduce women's conflict regarding their antihypertensive use in pregnancy and establish the effect of interventions on maternal concordance and health outcomes. However, without further evidence relating to the safety and effectiveness of common antihypertensives it is unclear if further reductions in maternal and fetal morbidity can be achieved through prescribing practices. Future research should also focus on active implementation of blood pressure target setting and pathways for those with outside of target blood pressure readings. This is likely to reduce morbidity as target blood pressure setting in pregnancy has been shown to reduce incidences of severe hypertension.[3,5] Policymakers may also wish to consider further studies that identify effective models and pathways of care for reducing adverse perinatal outcomes within the context of pregnancy hypertension.

**CONCLUSION**

Maternal and neonatal morbidity resulting from severe hypertension in pregnancy is prevalent. [1,4,5] This evaluation of the implementation of the NICE hypertension in pregnancy guidelines (2010)[6] addresses strategies to reduce the number of episodes of severe hypertension and has identified sub-optimal target setting practices, poor information provision for pregnant women and variability in prescribing practices. Women's non-adherence to antihypertensives is higher than previously reported and this is likely to be contributing to adverse perinatal outcomes. Analysis of the domains that influence implementation of the guidelines have identified that education and decision-making strategies are needed to address both clinician and women's behaviour. Further research into the effectiveness and long-term safety of common antihypertensives is also required.

a. Contributor statement – RW, LC and JS conceived of the study, the manuscript, and analyses, with contributions from LW, JG, HB and HW. RW was responsible for data management and data analysis. All authors reviewed, critically revised, and approved the manuscript.

For peer review only

b. Competing interests – None declared

c. Funding – This work was supported by the National Institute for Health Research (Research Professorship RP-2014-05-019) and by the National Institute for Health Research (NIHR) Collaboration for Leadership in Applied Health Research and Care South London (NIHR CLAHRC South London) at King's College Hospital NHS Foundation Trust. Jane Sandall is an NIHR Senior Investigator and is supported by the National Institute for Health Research (NIHR) Applied Research Collaboration South London (NIHR ARC South London) at King's College Hospital NHS Foundation Trust. The views expressed are those of the author[s] and not necessarily those of the NIHR or the Department of Health and Social Care.

d. Data sharing statement - All data relevant to the study are included in the article.

For peer review only

References

1. Bramham K, Parnell B, Nelson-Piercy C, et al. Chronic hypertension and pregnancy outcomes: systematic review and meta-analysis. *BMJ : British Medical Journal* 2014;348:g2301. doi: 10.1136/bmj.g2301

2. Knight M NM, Tuffnell D, Kenyon S, Shakespeare J, Brocklehurst P, Kurinczuk JJ (Eds.) on behalf of MBRRACE-UK. . Saving Lives, Improving Mothers' Care – Surveillance of maternal deaths in the UK 2012−14 and lessons learned to inform maternity care from the UK and Ireland Confidential Enquiries into Maternal Deaths and Morbidity 2009-14, 2016.

3. Magee LA, von Dadelszen P, Rey E, et al. Less-tight versus tight control of hypertension in pregnancy. *N Engl J Med* 2015;372(5):407-17. doi: 10.1056/NEJMoa1404595 [published Online First: 2015/01/30]

4. Flenady V, Koopmans L, Middleton P, et al. Major risk factors for stillbirth in high-income countries: a systematic review and meta-analysis. *Lancet* 2011;377(9774):1331-40. doi: 10.1016/S0140-6736(10)62233-7 [published Online First: 2011/04/19]

5. Magee LA, von Dadelszen P, Singer J, et al. The CHIPS Randomized Controlled Trial (Control of Hypertension in Pregnancy Study): Is Severe Hypertension Just an Elevated Blood Pressure? *Hypertension* 2016;68(5):1153-59. doi: 10.1161/HYPERTENSIONAHA.116.07862 [published Online First: 2016/09/14]

6. (NICE) NIfHCE. Hypertension in pregnancy CG107 2010 [Available from: https://www.nice.org.uk/guidance/cg107

7. (NICE) NIfHCE. Hypertension in pregnancy. Quality Standard (QS35), 2013.

8. (NICE) NIfHCe. Patient Experience in Adult NHS Services: Improving the Experience of Care for People Using Adult NHS Services: Patient Experience in Generic Terms. London2012.

9. Kirk MA, Kelley C, Yankey N, et al. A systematic review of the use of the Consolidated Framework for Implementation Research. *Implementation Science* 2016;11(1):72. doi: 10.1186/s13012-016-0437-z

10. Kirkpatrick DH, Burkman RT. Does Standardization of Care Through Clinical Guidelines Improve Outcomes and Reduce Medical Liability? *Obstetrics & Gynecology* 2010;116(5):1022-26. doi: 10.1097/AOG.0b013e3181f97c62

11. Hoffmann TC, Glasziou PP, Boutron I, et al. Better reporting of interventions: template for intervention description and replication (TIDieR) checklist and guide. *BMJ : British Medical Journal* 2014;348:g1687. doi: 10.1136/bmj.g1687

12. Burt J, Abel G, Elmore N, et al. Assessing communication quality of consultations in primary care: initial reliability of the Global Consultation Rating Scale, based on the Calgary-Cambridge Guide to the Medical Interview. *BMJ Open* 2014;4(3):e004339. doi: 10.1136/bmjopen-2013-004339

13. Braun V, Clarke V. Using thematic analysis in psychology. *Qualitative Research in Psychology* 2006;3(2):77-101. doi: 10.1191/1478088706qp063oa

14. Damschroder LJ, Aron DC, Keith RE, et al. Fostering implementation of health services research findings into practice: a consolidated framework for advancing implementation science. *Implementation science : IS* 2009;4:50. doi: 10.1186/1748-5908-4-50 [published Online First: 2009/08/12]

15. International consortium for health outcome measurements i. Pregnancy and childbirth standard set. 2016

16. Pope C, Ziebland S, Mays N. Qualitative research in health care. Analysing qualitative data. *BMJ* 2000;320(7227):114-16. doi: 10.1136/bmj.320.7227.114

17. O'Cathain A, Murphy E, Nicholl J. The quality of mixed methods studies in health services research. *Journal of health services research & policy* 2008;13(2):92-8. doi: 10.1258/jhsrp.2007.007074 [published Online First: 2008/04/18]

For peer review only

18. excellence Nifhac. Hypertension in adults: diagnosis and management (NG136). 2019

19. Vrijens B, Vincze G, Kristanto P, et al. Adherence to prescribed antihypertensive drug treatments: longitudinal study of electronically compiled dosing histories. *BMJ* 2008;336(7653):1114-17. doi: 10.1136/bmj.39553.670231.25

20. Dickinson D, Wilkie P, Harris M. Taking medicines: concordance is not compliance. *BMJ* 1999;319(7212):787. doi: 10.1136/bmj.319.7212.787

21. Rangachari P, Rissing P, Rethemeyer K. Awareness of evidence-based practices alone does not translate to implementation: insights from implementation research. *Quality management in health care* 2013;22(2):117-25. doi: 10.1097/QMH.0b013e31828bc21d [published Online First: 2013/04/02]

22. von Dadelszen P, Sawchuck D, McMaster R, et al. The Active Implementation of Pregnancy Hypertension Guidelines in British Columbia. *Obstetrics & Gynecology* 2010;116(3):659-66. doi: 10.1097/AOG.0b013e3181eb669d

23. Webster Louise M, Myers Jenny E, Nelson-Piercy C, et al. Labetalol Versus Nifedipine as Antihypertensive Treatment for Chronic Hypertension in Pregnancy. *Hypertension* 2017;70(5):915-22. doi: 10.1161/HYPERTENSIONAHA.117.09972

24. Lupattelli A, Spigset O, Nordeng H. Adherence to medication for chronic disorders during pregnancy: results from a multinational study. *International Journal of Clinical Pharmacy* 2014;36(1):145-53. doi: 10.1007/s11096-013-9864-y

25. Matsui D. Adherence with Drug Therapy in Pregnancy. *Obstetrics and Gynecology International* 2012;2012:5. doi: 10.1155/2012/796590

26. Abheiden CNH, van Reuler AVR, Fuijkschot WW, et al. Aspirin adherence during high-risk pregnancies, a questionnaire study. *Pregnancy Hypertension: An International Journal of Women's Cardiovascular Health* 2016;6(4):350-55. doi: https://doi.org/10.1016/j.preghy.2016.08.232

27. Abalos E, Duley L, Steyn DW, et al. Antihypertensive drug therapy for mild to moderate hypertension during pregnancy. *Cochrane Database Syst Rev* 2018;10:CD002252. doi: 10.1002/14651858.CD002252.pub4 [published Online First: 2018/10/03]

28. Excellence NIfHaC. Hypertension in pregnancy: diagnosis and management

NICE guideline [NG133]. 2019

Figure 1. Interpretation of integrated analysis: a strategy for improved implementation of evidence-based hypertension in pregnancy management

Figure 2a. Women's adherence and concordance with prescribed antihypertensives. Numbers 1-18 represent interviewed women and their experiences of anti-hypertensive prescribing during pregnancy. Women who experienced a change in their adherence or in the reporting of internal conflict are plotted more than once in different bubbles. 2b. Facilitators of women's adherence and of concordance.

For peer review only

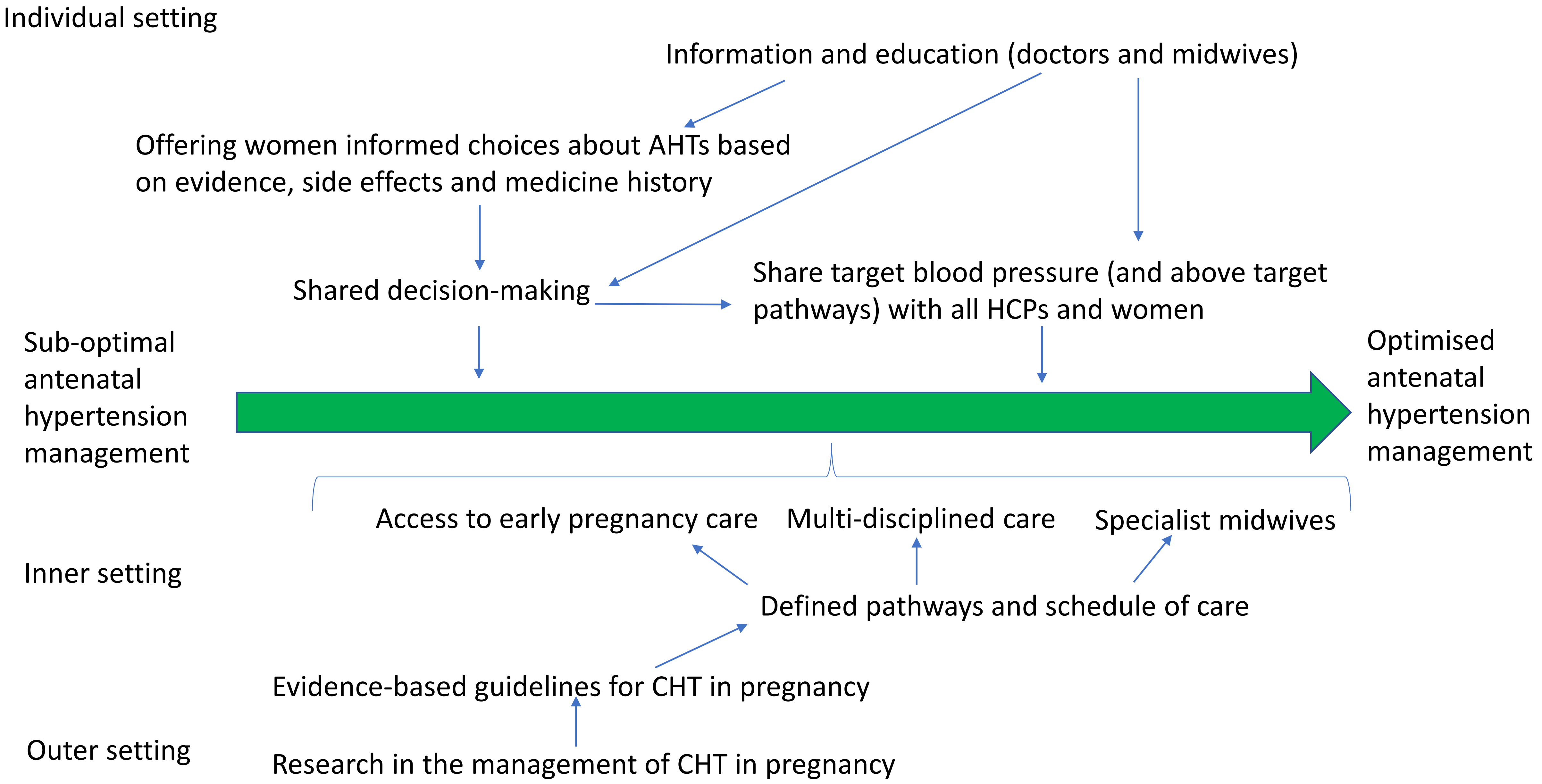
Individual setting
Information and education (doctors and midwives)
Offering women informed choices about AHTs based on evidence, side effects and medicine history
Shared decision-making
Share target blood pressure (and above target pathways) with all HCPs and women
Sub-optimal antenatal hypertension management
Optimised antenatal hypertension management
Access to early pregnancy care
Multi-disciplined care
Specialist midwives
Inner setting
Defined pathways and schedule of care
Evidence-based guidelines for CHT in pregnancy
Outer setting
Research in the management of CHT in pregnancy

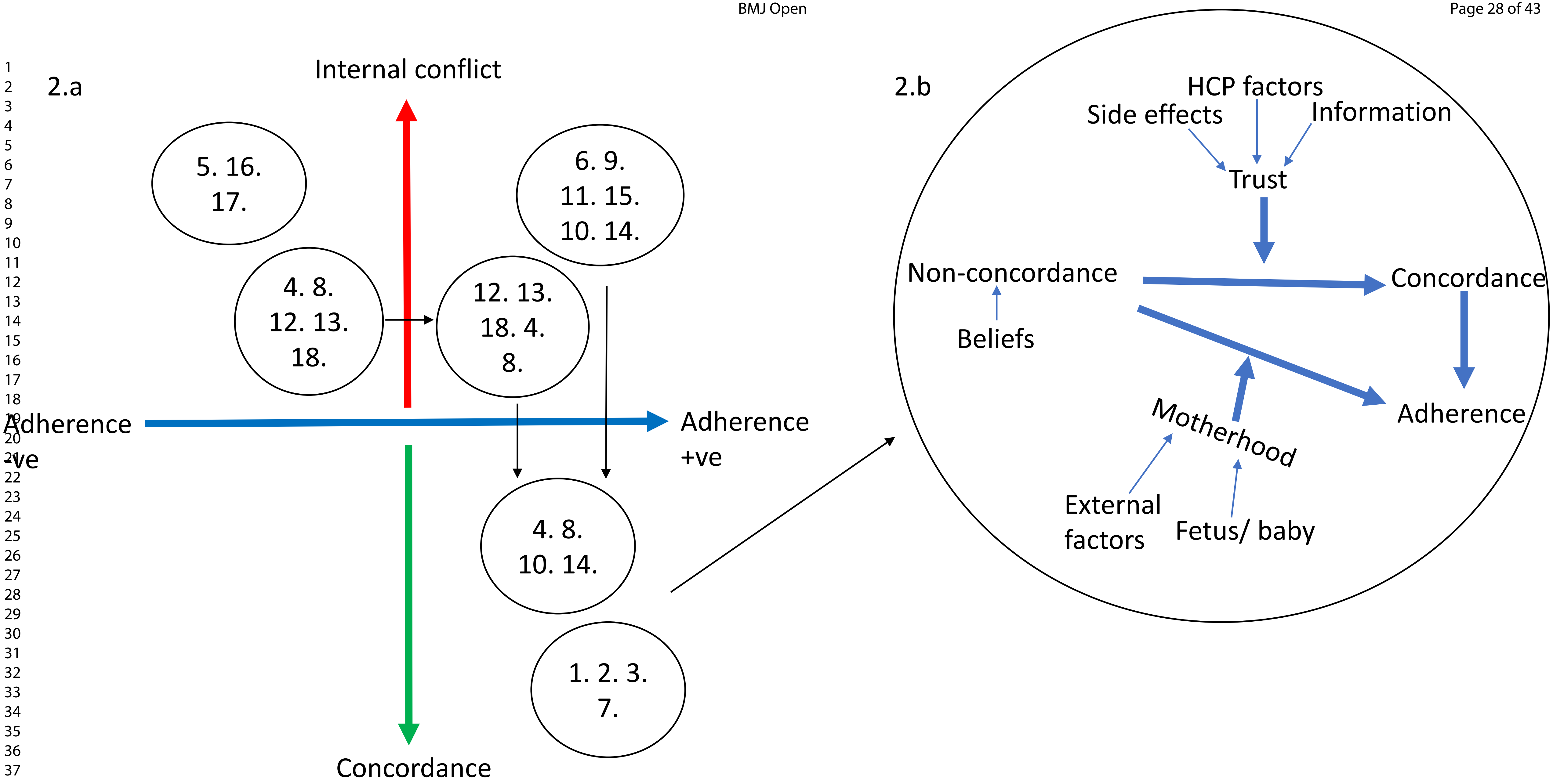
2.a
Internal conflict
5. 16. 17.
6. 9. 11. 15. 10. 14.
4. 8. 12. 13. 18.
12. 13. 18. 4. 8.
Adherence -ve
Adherence +ve
4. 8. 10. 14.
1. 2. 3. 7.
Concordance
2.b
HCP factors
Side effects
Information
Trust
Non-concordance
Concordance
Beliefs
Motherhood
Adherence
External factors
Fetus/ baby

Supplementary file 1

Chronic hypertension in pregnancy – healthcare professional survey

| Respondents | Number (97) | Percentage % |
|---|---|---|
| Obstetrician<br>Of which are consultants | 69<br>53 | 71.1<br>55 |
| Midwife<br>Of which are specialist/ senior midwife | 28<br>22 | 28.9<br>22.7 |
| NHS hospital trusts represented<br>(including England, Northern Ireland, Scotland and Wales) | 69 | - |

Question 1: If you see a pregnant woman with chronic hypertension who is currently taking either ACEIs or ARBs (e.g. at the beginning of pregnancy), how often would you ask her to stop taking them?

| Response | Number (97) | Percentage (%) |
|---|---|---|
| Always | 57 | 57.8 |
| Almost always | 27 | 27.8 |
| About two thirds of the time | 1 | 1 |
| About half of the time | 4 | 4.1 |
| About a third of the time | 0 | 0 |
| Very rarely | 1 | 1 |
| Never | 3 | 3.1 |
| Missing | 4 | 4.1 |

Question 2: What do you usually use as your first line anti-hypertensive treatment(s) for women with chronic hypertension in pregnancy?

| Anti-hypertensive (non-exclusive) | Number (97) | Percentage (%) |
|---|---|---|
| Labetalol | 85 | 87.6 |
| Methyldopa | 29 | 29.9 |
| Nifedipine | 32 | 33.0 |
| Amlodipine | 2 | 2.1 |

Question 3: What additional anti-hypertensive medication do you use for treating women with chronic hypertension in pregnancy?

| Anti-hypertensive (non-exclusive) | Number (97) | Percentage (%) |
|---|---|---|
| Amlodipine | 37 | 38.1 |
| Atenolol | 2 | 2.1 |
| Doxazosin | 23 | 23.7 |
| Enalapril | 1 | 1.0 |
| Hydralazine (oral) | 2 | 2.1 |
| Labetalol | 38 | 39.2 |
| Methyldopa | 60 | 61.9 |
| Metoprolol | 1 | 1.0 |
| Nifedipine | 79 | 81.4 |

For peer review only

Question 4: How frequently do you set a blood pressure target for women with chronic hypertension in pregnancy who need anti-hypertensive treatment (assuming no other co-morbidity) (mmHg)?

| Answer | Number (97) | Percentage % |
|---|---|---|
| Always | 36 | 37.1 |
| Almost always | 36 | 37.1 |
| About two thirds of the time | 8 | 8.2 |
| About half of the time | 3 | 3.1 |
| About a third of the time | 4 | 4.1 |
| Very rarely | 3 | 3.1 |
| Never | 1 | 1.0 |
| Other<br>In the guidelines but compliance unknown<br>Frequency not described | 6<br>2<br>4 | 6.2 |

Question 5: What blood pressure target do you usually set for pregnant women with chronic hypertension (assuming no other co-morbidity) (mmHg)?

| Systolic | Number (97) | Percentage % | Median (IQR1-IQR3) |
|---|---|---|---|
| 120 | 2 | 2.1 | |
| 125 | 0 | 0.0 | |
| 130 | 6 | 6.2 | |
| 135 | 2 | 2.1 | |
| 140 | 33 | 34.0 | |
| 145 | 0 | 0.0 | |
| 150 | 40 | 41.2 | |
| 155 | 1 | 1.0 | |
| 160 | 8 | 8.2 | |
| Missing | 4 | 4.1 | |
| Median | | | 150 (140-150) |

| Diastolic | Number (97) | Percentage % | Median (IQR1-IQR3) |
|---|---|---|---|
| 80 | 9 | 9.3 | |
| 85 | 7 | 7.2 | |
| 90 | 37 | 38.1 | |
| 95 | 8 | 8.2 | |
| 100 | 27 | 27.8 | |
| 110 | 3 | 3.1 | |
| Missing | 5 | 5.2 | |
| Median | | | 90 (90-100) |

For peer review only

Question 6: How often do you prescribe Aspirin for women with chronic hypertension in pregnancy?

| Answer | Number (97) | Percentage % |
|---|---|---|
| Always | 53 | 54.6 |
| Almost always | 36 | 37.1 |
| About two thirds of the time | 5 | 5.2 |
| About half of the time | 2 | 2.1 |
| Very rarely | 1 | 1.0 |

Question 7: At what gestation do these women usually receive their first Aspirin prescription?

| Answer | Number (97) | Percentage % |
|---|---|---|
| Before 12 weeks | 41 | 42.3 |
| 12-15+6 weeks | 52 | 53.6 |
| 16-19+6 weeks | 1 | 1.0 |
| Missing answer | 3 | 3.1 |

Question 8: For a woman with uncomplicated chronic hypertension in pregnancy (i.e. no additional risk factors), how many routine fetal growth scans do they receive (excluding nuchal and anomaly scans)?

| Additional scans | Number (97) | Percentage % | Median (IQR1-IQR3) |
|---|---|---|---|
| None | 4 | 4.1 | |
| 1 | 12 | 12.4 | |
| 2 | 23 | 23.7 | |
| 3 | 37 | 38.1 | |
| 4 | 21 | 21.6 | |
| >4 | 1 | 1.0 | |
| | | | 3 (2-3) |

Question 9: When do you usually plan birth for women with chronic hypertension whose blood pressure is controlled below 160/110?

| Gestation | Number (97) | Percentage (%) | Median (IQR1-IQR3) |
|---|---|---|---|
| Before 34 weeks | 3 | 3.1 | |
| 34-34+6 weeks | 2 | 2.1 | |
| 35-35+6 weeks | 2 | 2.1 | |
| 36-36+6 weeks | 4 | 4.1 | |
| 37-37+6 weeks | 27 | 27.8 | |
| 38-38+6 weeks | 36 | 37.1 | |
| 39-39+6 weeks | 41 | 42.3 | |
| 40-41 weeks | 28 | 28.9 | |
| Await spontaneous labour | 5 | 5.2 | |
| Other – individualised | 4 | 4.2 | |
| | | | 38.5 (37-39) |

For peer review only

Question 10: Involving pregnant women who have chronic hypertension in their pregnancy and birth planning is an important part of the consultation?

| **Sentiment** | **Number (97)** | **Percentage (%)** |
|---|---|---|
| Agree Strongly | 79 | 81.4 |
| Agree | 15 | 15.5 |
| Slightly Agree | 2 | 2.1 |
| Slightly disagree | 0 | 0.0 |
| Disagree | 0 | 0.0 |
| Disagree Strongly | 1 | 1.0 |

Question 11: If you wish, can you give an example of how you enable women to be actively involved in their care?

| **Themes** | **Number (47)** | **Percentage %** |
|---|---|---|
| Total responses | 47 | |
| SDM in the following areas | | |
| • Home BP | 10 | 21 |
| • Monitoring BP | 6 | 12.8 |
| • Anti-hypertensives | 5 | 10.6 |
| • Planning birth (induction of labour) | 17 | 36 |
| • Organisation of care | 4 | 8.5 |
| Discussing risks and benefits | 2 | 4.3 |
| How to identify pre-eclampsia | 2 | 4.3 |

Question 12: In your maternity unit what term/s best describes the antenatal care provided to most women with chronic hypertension?

| **Care provision** | **Number (97)** | **Percentage %** |
|---|---|---|
| Named consultant-led general antenatal clinic (maternal medicine clinic) | 63 (7) | 64.9 (7.2) |
| Consultant-led specialist hypertension in pregnancy clinic | 25 | 25.8 |
| Multi-disciplinary clinic with additional medical professional | 20 | 20.6 |
| Consultant obstetrician and midwife antenatal clinic | 15 | 15.5 |
| Shared-care GP/ obstetrician/ midwife | 7 | 7.2 |
| Specialist midwifery care (e.g. medical conditions team) | 6 | 6.2 |
| Hospital midwifery care | 1 | 1.0 |
| Community based midwifery care | 4 | 4.1 |
| Day assessment unit | 2 | 2.1 |

For peer review only

Question 13: In your maternity unit when do the pregnant women with chronic hypertension usually first get seen by an obstetrician?

| Gestation | Number (97) | Percentage % |
|---|---|---|
| Before 12 weeks | 24 | 24.7 |
| 12-15+6 weeks | 63 | 64.9 |
| 16-27+6 weeks | 9 | 9.3 |
| Missing data | 1 | 1.0 |

Question 14: Do you or someone in your unit specifically collect and analyse the outcomes of women with chronic hypertension in pregnancy annually?

| Response | Number (97) | Percentage (%) |
|---|---|---|
| Yes | 24 | 24.7 |
| No | 67 | 69.0 |
| Unsure | 4 | 4.1 |
| Some aspects | 2 | 2.0 |

For peer review only

Supplementary file 2

**Interview topic guide for clinicians:**

- Descriptions of the general approach to practice and how clinicians approach treatment decisions
- Discussion about the sources of evidence and knowledge that influence practice in general
- Participants' beliefs and experiences of using or having contact with clinical guidance (NICE in particular),
- Participants' views regarding how EBM and clinical guidelines could be better mobilised into practice

Interview schedule:

- Introductions
- Confidentiality
- I am interviewing you today for the CHAMPION study about chronic hypertension, you provide antenatal care for women with CHP is that right?
- Can you tell me a about your CHP clinic and your clinical practice in relation to chronic hypertension in pregnancy?
- How do you approach decision-making, for example commencing or changing hypertensive medication or delivery the baby early?
- What are you views and experiences of involving women in decision about their care or treatment plan?
- How do you source evidence and develop knowledge around hypertension in pregnancy?
- What are you experiences of clinical guidance e.g. NICE/ RCOG?
- How do you think we could better implement evidence-based medicine into clinical practice?

Reference: Grove, A., Clarke, A. and Currie, G. (2015) 'The barriers and facilitators to the implementation of clinical guidance in elective orthopaedic surgery: a qualitative study protocol', Implementation Science, 10(1), 81.

**Women's experience of their care**

- Introductions
- Confidentiality
- During this pregnancy you have been treated for chronic hypertension is that right?
- Can you tell me a bit about your high blood pressure and your pregnancy?
- How satisfied are you with the results of your care during your pregnancy?
- Thinking about your care during your pregnancy…Were you given information about your choices for maternity care?
- Were you given enough information to help you decide about your care?
- Were you given information at the right time to help you decide about your care?
- Did you have confidence and trust in the staff caring for you?

Reference: International Consortium for Health Outcomes Measurement. Pregnancy and Childbirth Standard Set and Reference Guide. 2016. http://www.ichom.org/medical-conditions/pregnancy-and-childbirth/.

For peer review only

Supplementary file 3

Maternal demographics of women observed, interviewed and included for case-note review. Women interviewed are a subset of those observed. Case-notes identified for review are a different cohort of women.

| Women demographics | Observed n=28 (%) | Interviewed n=18 (%) | Case-notes n=55 (%) |
|---|---|---|---|
| **Ethnicity** | | | |
| White British | 9 (32.0) | 7 (39.0) | 15 (27.3) |
| White Other | 6 (21.0) | 4 (22.0) | 8 (14.5) |
| Black | 9 (32.0) | 5 (28.0) | 18 (32.7) |
| Asian | 2 (7.0) | 1 (5.5) | 8 (14.5) |
| Any other | 2 (7.0) | 1 (5.5) | 6 (10.9) |
| **Parity at booking** | | | |
| 0 | 9 (32.0) | 7 (39.0) | 15 (27.3) |
| 1 | 11(39.0) | 7 (39.0) | 21 (38.2) |
| 2 | 7 (25.0) | 4 (22.0) | 10 (18.2) |
| 3 | 0 (0.0) | 0 (0.0) | 6 (10.9) |
| 4 | 0 (0.0) | 0 (0.0) | 2 (3.6) |
| 5 | 1 (4.0) | 0 (0.0) | 1 (1.8) |
| **Age** | | | |
| 20-34 | 17 (61.0) | 11 (61.0) | 23 (41.8) |
| 35-39 | 7 (25.0) | 5 (28.0) | 21 (38.9) |
| 40-44 | 4 (14.0) | 2 (11.0) | 11 (20.4) |
| **BMI** | | | |
| <18.5 | 0 (0.0) | 0 (0.0) | 1/52 (1.9) |
| 18.5-24.9 | 7 (25) | 6 (33.3) | 13/52 (25.0) |
| 25-29.9 | 10 (36) | 6 (33.3) | 13/52 (25.0) |
| 30-34.9 | 9 (32) | 5 (28.0) | 11/52 (21.2) |
| 35-39.0 | 2 (7) | 1 (5.5) | 6/52 (11.5) |
| >40.0 | 0 (0) | 0 (0.0) | 8/52 (7.7) |

For peer review only

Supplementary file 4

Pregnancy and birth outcomes – Case notes review

| Outcomes | Case notes review<br>Nominator/denominator (%) |
|---|---|
| Women with episode of severe hypertension | 25/55 (45.5) |
| 1st trimester episode | 2/40 (5.0) |
| 2nd trimester episode | 13/40 (32.5) |
| 3rd trimester episode | 25/40 (62.5) |
| Birth weight - median<br>(IQR1 – IQR3) | 2927.5<br>(2592.5 - 3200) |
| Admission to NNU | 9/55 (16.4) |

For peer review only

Supplementary file 5

Target blood pressure setting and prescribing practices per Trust – as derived from case-note review

| | **Hospital Trust 1 n=29 (%)** | **Hospital Trust 2 n=13 (%)** | **Hospital Trust 3 n=13 (%)** |
|---|---|---|---|
| **Target BP documented <150/100mmHg** | 20/26 (77.0) | 3/13 (23.0) | 5 (38.0) |
| **Labetalol** | 12/26 (46.0) | 7/12 (58.3) | 9/11 (82.0) |
| **Nifedipine** | 9/26 (34.5) | 0/12 (0.0) | 0/11 (0.0) |
| **Methyldopa** | 3/26 (11.5) | 4/12 (33.3) | 1/11 (9.0) |
| **Other** | 2/26 (8.0) | 1/12 (8.3) | 1/11 (9.0) |

For peer review only

Original research

# The quality of mixed methods studies in health services research

**Alicia O'Cathain, Elizabeth Murphy[1], Jon Nicholl**

Medical Care Research Unit, School of Health and Related Research, University of Sheffield, Sheffield; [1]School of Sociology and Social Policy, University of Nottingham, Nottingham, UK

***Objectives*: To assess the quality of mixed methods studies in health services research (HSR).**

***Methods*: We identified 118 mixed methods studies funded by the Department of Health in England between 1994 and 2004, and obtained proposals and/or final reports for 75. We applied a set of quality questions to both the proposal and report of each study, addressing the success of the study, the mixed methods design, the individual qualitative and quantitative components, the integration between methods and the inferences drawn from completed studies.**

***Results*: Most studies were completed successfully. Researchers mainly ignored the mixed methods design and described only the separate components of a study. There was a lack of justification for, and transparency of, the mixed methods design in both proposals and reports, and this had implications for making judgements about the quality of individual components in the context of the design used. There was also a lack of transparency of the individual methods in terms of clear exposition of data collection and analysis, and this was more a problem for the qualitative than the quantitative component: 42% (19/45) versus 18% (8/45) of proposals ($p = 0.011$). Judgements about integration could rarely be made due to the absence of an attempt at integration of data and findings from different components within a study.**

***Conclusions*: The HSR community could improve mixed methods studies by giving more consideration to describing and justifying the design, being transparent about the qualitative component, and attempting to integrate data and findings from the individual components.**

*Journal of Health Services Research & Policy* Vol 13 No 2, 2008: 92–98 © The Royal Society of Medicine Press Ltd 2008

## Introduction

Mixed methods studies are common in health services research (HSR).[1] They consist of two separate components of data collection and analysis within a single study: at least one quantitative method with structured data collection and statistical analysis, and at least one qualitative method with less structured data collection and thematic analysis.[2] Commissioners and consumers of research, as well as researchers themselves, need to judge whether a mixed methods study has been undertaken well or poorly, assessing whether it is good mixed methods research as well as good research. The quality of mixed methods research has been considered explicitly in health, educational and social research,[3–8] and implicitly when researchers have discussed the challenges of designing and implementing these studies.[9,10] However, the issue has received little consideration overall, with a recent search for quality criteria for mixed methods research concluding that there were none available,[7] even though attempts have been made to develop them.[3] Given that there are no agreed criteria for assessing the quality of these studies,[8] and that researchers are still debating the meaning of quality for mixed methods research,[6] it is premature to attempt to develop definitive criteria. Instead, it seems sensible to follow an approach taken by researchers considering quality in the context of synthesizing qualitative and quantitative evidence[11] and devise a set of questions which could be applied to mixed methods primary research to facilitate judgements about quality. We devised a set of 'quality questions' and applied them to proposals and reports of mixed methods studies to assess the quality of mixed methods studies in HSR.

**Alicia O'Cathain PhD**, MRC Fellow, **Jon Nicholl MSc**, Professor, Medical Care Research Unit, School of Health and Related Research, University of Sheffield, Regent Street, Sheffield S1 4DA, UK; **Elizabeth Murphy PhD**, Professor, School of Sociology and Social Policy, University of Nottingham, Nottingham, UK.

Correspondence to: a.ocathain@sheffield.ac.uk

## Methods

This research was part of a wider study exploring the use of mixed methods research in HSR. The wider study consisted of a quantitative documentary analysis of 75 mixed methods studies to determine the type

 DOI: 10.1258/jhsrp.2007.007074

For peer review only - http://bmjopen.bmj.com/site/about/guidelines.xhtml

and quality of mixed methods research undertaken, and qualitative interviews with 20 researchers to explore facilitators and barriers to exploiting the potential of this approach.[1,12]

## Devising questions about quality

We devised a framework for the quality assessment based on detailed consideration of the literature on mixed methods research in the fields of health, social and educational research. We searched the health databases MEDLINE and CINAHL. We then sought expert opinion encapsulated in key textbooks.[10,13–20] Finally we searched the Social Science Citations Index, PsycINFO, ERIC and the British Education Index to identify social, behavioural and educational research. The search for literature took place in 2003 and was updated in 2006. Quality was one of 11 issues identified in this review.

Within the literature, one suggested assessment criterion for mixed methods studies was whether they had been completed successfully in terms of adequately addressing the research questions with allocated resources.[5] Other researchers focused on the quality of methods. There was no suggestion of using a tool developed for generic use across all designs. Rather, researchers attempted to develop quality criteria by devising separate lists of criteria for the quantitative and the qualitative research.[7] Their assumption was that methods are linked to paradigms and therefore the criteria used to assess different methods should also be linked to paradigms.[7] However, not everyone agrees that methods are paradigm-specific[18] or that different criteria are needed for qualitative and quantitative research.[21] The same criteria have been proposed for both[21] although the appropriate means for judging against these criteria may differ because of the research practices employed in different methodological approaches. The mixed methods design[10] and the integration between methods[3] can be assessed as well as the individual methods. A good mixed methods study clearly justifies why a mixed methods approach is necessary or superior to another, offers transparency of the mixed methods design, and offers appropriate sampling, data collection and analysis of individual components relating to that design.[3,4,10] Thus the design may determine the criteria used to make judgements about the individual components of the study. Integration of data or findings from each component is a key part of mixed methods research,[10] distinguishing it from qualitative and quantitative studies undertaken independently. When integration occurs, it is important that data transformations are defensible, that contradictory findings are explained and convergent findings are not related to shared bias between methods.[3] Expertise may be needed within a research team to integrate at the analysis stage.[22] Finally, researchers have discussed the importance of inferences from mixed methods studies being trustworthy[6] and appropriate in the light of the design used.[3] As yet there are no criteria for assessing the quality of inferences from mixed methods research, although researchers are considering the complexity of this issue.[23]

When developing the framework for our quality questions we chose not to use a generic tool because they have variable applicability across different research designs.[24] We chose to assess the qualitative and quantitative components separately because they each contribute to the study as a whole and because the quality of one or both components may suffer as a consequence of being part of a mixed methods study.[25–27] In addition to the individual components, we included an assessment of the success of the study, the design, the integration and the inferences. Within this framework we constructed questions based on the literature review and reading the proposals and reports from four mixed methods studies in HSR.

## Identifying mixed methods studies

In 2004, mixed methods studies were identified through a systematic search of summaries of studies funded by the Department of Health, a key commissioner of health services research in England at that time. The methods have been described elsewhere[1,12] and are summarized here. Summaries of single studies funded between 1994 and 2004 through 10 programmes were read. The programmes were: Health Technology Assessment; Service Delivery and Organization; New and Emerging Applications of Technology; Policy Research Programme; and the NHS Research & Development programmes of maternal and child health, primary and secondary care interface, cardiovascular disease and stroke, forensic mental health, primary dental care, and promoting implementation of research findings. A total of 118 mixed methods studies were identified. The lead researcher of each study was written to with a request for the research proposal, the final report for completed studies and a list of any emerging publications.

## Application of quality questions

A data extraction form was devised which consisted of the quality questions with the tick box options of 'yes', 'yes, but improvements are possible', 'no', 'not enough information (NEI)' and 'not applicable (N/A)'. Space for open comments was available alongside each question, where the assessor (AOC) could record details of good and poor practice. The data extraction form was applied to each study by one researcher, first to the proposal and then to the report. Finally, any differences between the proposal and report were noted.

## Analysis

The structured data were entered into SPSS. The main analysis was descriptive, displaying the proportions of proposals and reports falling into each category of each question. The chi-squared test was used when

For Peer Review Only

comparing results for the individual qualitative and quantitative components. Open comments were quantitized[28] by transcribing them into Word, grouping them into themes, and counting the number of studies in which a theme occurred.[29]

## Results

Documentation was received for 75 mixed methods studies. Full proposals were obtained for 60% (45/75) of the studies. Final reports were only available for the 52 studies completed by the time of data collection, and were obtained for 92% (48), although one was a summary report that was too brief for inclusion in the assessment of quality, leaving 47 reports. Both a proposal and report was available for 20 studies.

### Success

The potential to produce a successfully completed study was assessed using the research proposals. In most proposals, the quantitative methods appeared to be feasible within the time and money allocated (Table 1). However, even recognizing that some aspects of qualitative research cannot be fixed at the design stage (e.g. sample size for theoretical sampling), there was not enough detail to determine the feasibility of the qualitative methods in one-third of studies – for example, no indication of numbers of interviews to be undertaken or no indication of when the qualitative research would be conducted in the study timetable. We had concerns about the feasibility of the qualitative component in another one-third of proposals. From the open comments we identified 14 proposals where a large number of qualitative interviews were planned in a short time scale – for example, 40 interviews in four months without specifying the depth of interview and analysis. In nine of these studies the report was available and in four cases considerably fewer interviews were undertaken than planned. However, concerns highlighted about the feasibility of the qualitative research did not necessarily translate into shortfalls in the final study.

We defined a successful study as one that produced everything that had been planned at the proposal stage. A direct comparison of the final study report with the proposal was only possible on the subset of 20 studies for which both were available. In other cases the assessment relied on researchers detailing the planned and implemented study within their final report. Non-completion of a whole component of a study was rare (Table 1). However, in one-fifth of reports, one of the methods within a component was not executed as planned. This tended to be due to a range of problems in the field.

### Mixed methods design

A justification for using mixed methods research was only given in one-third of proposals and reports (Table 2). A minority of studies explicitly articulated the design in terms of the priority of methods, the purpose of combining methods, the sequence of methods and the stage at which integration would or did occur. It was particularly helpful for the subsequent quality assessment of individual components if researchers were explicit about the priority of methods and the role of any less dominant method. For example, it seemed inappropriate to have 40 in-depth interviews as a preliminary aid to develop a questionnaire, but appropriate if these interviews were also to be used as a primary means of investigating the issue under study. A lack of transparency of the overall design could occur in the context of excellent description of individual components.

When the design was not discussed explicitly it was usually possible to work out the key elements from reading the documentation. In most cases the design was assessed as appropriate for addressing the research question. However, researchers rarely discussed issues of rigour in relation to the design employed. An example of addressing rigour for the design was where researchers proposed that qualitative findings would not be shared with quantitative colleagues undertaking a randomized controlled trial to minimize the possibility of contamination of that trial; in another two studies, the qualitative research was undertaken with people not participating in the trial in order to avoid contaminating the trial. While the extent to which this attention to contamination avoidance was necessary may be debatable, it constitutes some evidence that researchers had given serious consideration to design issues related to mixed methods research.

For peer review only

**Table 1** Assessment of the success of mixed methods studies in HSR

| | **Proposal (*n* = 45)** | | | | **Report (*n* = 47)** | | | |
|---|---|---|---|---|---|---|---|---|
| | **Yes** | **Yes, but** | **No** | **NEI or N/A** | **Yes** | **Yes, but** | **No** | **NEI or N/A** |
| 1 Is the quantitative component feasible? | 82% | 2% | 4% | 11% | | | | |
| 2 Is the qualitative component feasible? | 38% | 20% | 13% | 29% | | | | |
| 3 Is the mixed methods design feasible? | 51% | 0% | 7% | 42% | | | | |
| 4 Have both qualitative and quantitative components been completed? | | | | | 87% | 6% | 2% | 4% |
| 5 Were some quantitative methods planned but not executed? | | | | | 19% | 0% | 45% | 36% |
| 6 Were some qualitative methods planned but not executed? | | | | | 21% | 2% | 38% | 38% |
| 7 Did the mixed methods design work in practice? | | | | | 85% | 0% | 2% | 13% |

NEI, not enough information; N/A, not applicable


For peer review only - http://bmjopen.bmj.com/site/about/guidelines.xhtml

**Table 2** Assessment of the mixed methods design of studies in HSR

| | Proposal (*n* = 45) | | | | Report (*n* = 47) | | | |
|---|---|---|---|---|---|---|---|---|
| | Yes | Yes, but | No | NEI or N/A | Yes | Yes, but | No | NEI or N/A |
| 1 Is the use of mixed methods research justified? | 31% | 3% | 60% | 4% | 30% | 2% | 66% | 2% |
| 2 Is the design for mixing methods described? | | | | | | | | |
| Priority | 16% | 2% | 78% | 4% | 15% | 0 | 83% | 2% |
| Purpose | 42% | 0 | 53% | 4% | 34% | 4% | 60% | 2% |
| Sequence | 56% | 0 | 40% | 4% | 49% | 0 | 49% | 2% |
| Stage of integration | 24% | 0 | 71% | 4% | 21% | 0 | 77% | 2% |
| 3 Is the design clearly communicated? | 80% | 0 | 16% | 4% | 81% | 4% | 9% | 6% |
| 4 Is the design appropriate for addressing the research questions? | 87% | 2% | 2% | 9% | 87% | 0% | 2% | 11% |
| 5 Has rigour of the design been considered (proposal) or adhered to (report)? | 7% | 0 | 93% | 0% | 21% | 0% | 0% | 79% |

NEI, not enough information; N/A, not applicable

## Quantitative component

The roles of the quantitative methods were usually communicated well within proposals and reports (Table 3). However, sufficient details were sometimes not given about these methods. In eight proposals the quantitative methods were only sketchily described and in a further 13 proposals some aspects of the quantitative methods were not described, in particular, the analysis (8) and the numbers involved (5). This was less of an issue for reports but nonetheless there were still problems with sketchy description overall (4) or little or no description of the analysis (5). This lack of transparency made it difficult to assess other aspects of quality.

Validity of the methods within the quantitative components was assessed by considering the attention researchers gave to issues such as confounding and bias. Validity was explicitly discussed in two-thirds of proposals, with little evidence that the rigour of any method was compromised (Table 3). There were few examples of an individual method being compromised by the mixed methods approach. One example was a Delphi exercise which was restricted in order to fit the timetable of the qualitative fieldwork.

It was difficult to determine the sophistication of proposed analyses due to the lack of detail about analysis in the research proposals. There was more information about analyses available in research reports and here concerns were identified about the sophistication of one-quarter of quantitative analyses. We identified 12 studies where the reported quantitative results seemed simplistic, sometimes only presenting descriptive statistics with no statistical tests and in two cases using an experimental design which was then ignored in the analysis.

## Qualitative component

The roles of the qualitative methods were usually communicated well within proposals and reports (Table 4). However, qualitative methods were often not described in sufficient detail and this occurred more frequently than for the quantitative components, both within proposals ($p = 0.011$) and reports ($p = 0.08$). First, there was sketchy description of the qualitative methods overall (15 proposals and 11 reports). In three of these reports there was no description of the qualitative methods at all, only the findings. Second, there were no details about an important aspect of the qualitative research, particularly the analysis (six proposals and nine reports). Third, one method was described in detail, usually interviews with a particular group, but a further qualitative method such as observation or focus groups appeared to be 'tagged on' with no description (six proposals). Fourth, the overall size of the qualitative component was not clear, with a few

**Table 3** Assessment of the quantitative component of mixed methods studies in HSR

| | Proposal (*n* = 45) | | | | Report (*n* = 47) | | | |
|---|---|---|---|---|---|---|---|---|
| | Yes | Yes, but | No | NEI or N/A | Yes | Yes, but | No | NEI or N/A |
| 1 Is the role of each method clear? | 98% | 0% | 2% | 0% | 96% | 2% | 0% | 2% |
| 2 Is each method described in sufficient detail? | 53% | 29% | 18% | 0% | 68% | 13% | 15% | 4% |
| 3 Is each method appropriate for addressing the research question? | 93% | 0 | 2% | 4% | 98% | 0% | 0% | 2% |
| 4 Is the approach to sampling and analysis appropriate for its purpose? | 67% | 4% | 4% | 24% | 70% | 9% | 6% | 15% |
| 5 Is there expertise among applicants/authors? | 67% | 2% | 7% | 24% | 30% | 0% | 0% | 70% |
| 6 Is there expertise on the team to undertake each method? | 60% | 0% | 2% | 24% | | | | |
| 7 Have issues of validity been addressed for each method? | 64% | 0% | 30% | 7% | 49% | 4% | 40% | 6% |
| 8 Has the rigour of any method been compromised? | 7% | 0% | 91% | 2% | 9% | 4% | 83% | 4% |
| 9 Is each method sufficiently developed for its purpose? | 84% | 0% | 7% | 9% | 83% | 0% | 4% | 13% |
| 10 Is the (intended) analysis sufficiently sophisticated? | 56% | 4% | 2% | 38% | 51% | 15% | 25% | 9% |

NEI, not enough information; N/A, not applicable

For peer review only - http://bmjopen.bmj.com/site/about/guidelines.xhtml

**Table 4** Assessment of the qualitative component of mixed methods studies in HSR

| | Proposal (*n* = 45) | | | | Report (*n* = 47) | | | |
|---|---|---|---|---|---|---|---|---|
| | Yes | Yes, but | No | NEI or N/A | Yes | Yes, but | No | NEI or N/A |
| 1 Is the role of each method clear? | 87% | 0% | 9% | 4% | 92% | 4% | 4% | 0% |
| 2 Is each method described in sufficient detail? | 24% | 29% | 42% | 4% | 38% | 28% | 30% | 4% |
| 3 Is each method appropriate for addressing the research question? | 87% | 7% | 2% | 4% | 91% | 2% | 2% | 4% |
| 4 Is the approach to sampling and analysis appropriate for its purpose? | 42% | 4% | 9% | 40% | 53% | 9% | 4% | 34% |
| 5 Is there expertise among the applicants/authors? | 56% | 2% | 11% | 31% | 32% | 4% | 0% | 64% |
| 6 Is there expertise on the team to undertake each method? | 44% | 9% | 7% | 40% | | | | |
| 7 Have issues of validity been addressed for each method? | 24% | 0% | 64% | 11% | 30% | 2% | 57% | 11% |
| 8 Has the rigour of any method been compromised? | 2% | 0% | 91% | 7% | 6% | 2% | 81% | 11% |
| 9 Is each method sufficiently developed for its purpose? | 64% | 0% | 9% | 27% | 77% | 2% | 9% | 13% |
| 10 Is the (intended) analysis sufficiently sophisticated? | 40% | 4% | 7% | 49% | 51% | 13% | 19% | 17% |

NEI, not enough information; N/A, not applicable

interviews here and there throughout the study adding up to a sizeable qualitative component of over 100 interviews (10 proposals).

Validity of the methods within the qualitative components was assessed by considering the attention researchers gave to issues such as reflexivity and negative cases. Validity was not addressed within proposals for more qualitative than quantitative components ($p = 0.001$), although any apparent difference in reports was not statistically significantly different ($p = 0.100$) (Table 4). Researchers did take the validity of qualitative methods seriously in some proposals, for example, paying attention to deviant cases and peer review of transcripts.

Concerns were identified with the sophistication of one-fifth of qualitative analyses. In nine studies the reported qualitative findings remained at a descriptive level, or reported findings in a quantitative manner only, or failed to distinguish between data collected using different methods such as focus groups and interviews.

### Integration

Integration of data or findings from the different methods received little attention in either proposals or reports, with researchers rarely discussing the type of integration, how it occurred in the context of team working and who was involved in it (Table 5). Because of the lack of integration, questions about the appropriateness of integration and the effect of integration on the rigour of individual methods were irrelevant.

### Inferences

In the reports, researchers were clear about which results had emerged from which methods, and inferences seemed appropriate (Table 6). For one-fifth of studies there was a concern that the inferences were based disproportionately on one method rather than the findings of all the methods. The imbalance was as likely to be towards qualitative findings as it was towards quantitative findings.

## Discussion

### The quality of studies in HSR

Mixed methods studies tend to be successful in HSR insofar that the qualitative and quantitative components are usually completed as planned. The main quality issue identified was a lack of transparency of the

**Table 5** Assessment of integration in mixed methods studies in HSR

| | Proposal (*n* = 45) | | | | Report (*n* = 47) | | | |
|---|---|---|---|---|---|---|---|---|
| | Yes | Yes, but | No | NEI or N/A | Yes | Yes, but | No | NEI or N/A |
| 1 Is the type of integration stated? | 11% | 0% | 84% | 4% | 2% | 2% | 94% | 2% |
| 2 Is the type of integration appropriate to the design? | 16% | 0% | 0% | 84% | 34% | 0% | 2% | 64% |
| 3 Has enough time been allocated for integration? | 2% | 0% | 13% | 85% | | | | |
| 4 Is the approach to integration detailed in terms of working together as a team? | 7% | 0% | 80% | 13% | | | | |
| 5 Does the dissemination strategy detail how the mixed methods will be reported in final reports and peer-reviewed publications? | 0% | 0% | 84% | 16% | | | | |
| 6 Are the personnel who participate in the integration clearly identified? | 9% | 0% | 80% | 11% | 6% | 0% | 70% | 23% |
| 7 Did appropriate members of the team participate in integration? | | | | | 0% | 0% | 2% | 98% |
| 8 Is there evidence of communication within the team? | | | | | 19% | 0% | 6% | 75% |
| 9 Has rigour been compromised by the process of integration? | | | | | 4% | 0% | 0% | 96% |

NEI, not enough information; N/A, not applicable

For peer review only


For peer review only - http://bmjopen.bmj.com/site/about/guidelines.xhtml

**Table 6** Assessment of the inferences made in completed reports of mixed methods studies in HSR

| | Report (*n* = 47) | | | |
|---|---|---|---|---|
| | Yes | Yes, but | No | NEI or N/A |
| 1 Is there clarity about which results have emerged from which methods? | 87% | 2% | 6% | 4% |
| 2 Are inferences appropriate? | 83% | 4% | 9% | 4% |
| 3 Are the results of all the methods considered sufficiently in the interpretation? | 66% | 6% | 19% | 9% |

NEI, not enough information; N/A, not applicable

mixed methods aspects of the studies and the individual components. The qualitative components were more likely to be poorly described than the quantitative ones. To some extent the poor description of qualitative methods is not a surprising finding given the historical dominance of quantitative methods in HSR. However, it raises concerns that the HSR community may be failing on occasions to exploit the potential of qualitative methods within mixed methods studies. Where a qualitative component is in a supporting role to a more dominant method, and does not have stand-alone status in terms of independently addressing an aspect of the research question, then limited description is acceptable. However, because researchers were often not explicit about the status of methods within the study design, it was difficult to make judgements about the individual components in the context of the design used. Integration of data and findings is a key part of mixed methods research. There was no evidence that inappropriate integration was undertaken because there was a tendency for researchers to keep the qualitative and quantitative components separate rather than attempt to integrate data or findings in reports or publications.[12]

## Developing quality criteria for mixed methods studies in HSR

There was a lack of transparency in the reporting of mixed methods studies in HSR which made it difficult to assess other aspects of the quality of these studies. This has been identified as a problem facing the quality assessment of other types of studies[11] and has led to the development of guidelines for reporting studies. Creswell has suggested a list of issues to consider when designing a mixed methods study[10] and we have considered this in conjunction with the literature on the quality of mixed methods studies to suggest some guidelines for Good Reporting of A Mixed Methods Study (GRAMMS) (Box 1). We present this as guidance for researchers rather than as a formal checklist.

**Box 1** Good Reporting of A Mixed Methods Study (GRAMMS)

(1) Describe the justification for using a mixed methods approach to the research question
(2) Describe the design in terms of the purpose, priority and sequence of methods
(3) Describe each method in terms of sampling, data collection and analysis
(4) Describe where integration has occurred, how it has occurred and who has participated in it
(5) Describe any limitation of one method associated with the present of the other method
(6) Describe any insights gained from mixing or integrating methods

## Limitations

The study is based on mixed methods research funded by one commissioner in one country. The response rate to requests for documentation for mixed methods studies was good but non-responders may have been more likely to be problematic studies, biasing the findings towards higher quality studies. The questions were devised and applied by one researcher (AOC) in the context of team discussions which meant that the data extraction process was unchallenged by an external source. A coding protocol was devised to accompany the data extraction form to aid transparency and reduce intra-rater variability. However the studies could have been rated differently by another researcher. Finally, the studies included were funded between 1994 and 2004 and improvements may have occurred since then.

We have taken a technical stance in our discussions of quality in mixed methods research. However, the philosophical stance adopted by researchers may affect the quality criteria they use, and wish to see applied to their studies. Subtle realism[30] has been proposed as a philosophical position appropriate for qualitative and quantitative research in health technology assessment.[21] An implication of this stance is that researchers would need to consider whether reflexivity has been applied to the whole of a mixed methods study rather than simply the qualitative component.

## Conclusions

This is the first attempt to consider the quality of mixed methods studies in HSR. We are not offering this as a definitive approach to be used by others, but to start the debate about how to assess and improve quality. We recommend that if we use mixed methods studies in HSR then we need to be more transparent about the design and the individual components in the context of the design, and attempt to integrate data and findings from the qualitative and quantitative methods.

## Acknowledgements

Many thanks to the researchers who kindly sent copies of their study documents. The Medical Research Council funded the study through their Fellowship scheme.

For peer review only

## References

1 O'Cathain A, Murphy E, Nicholl J. Why, and how, mixed methods research is undertaken in health services research: a mixed methods study. *BMC Health Serv Res* 2007;**7**:85
2 Bryman A. Quantitative and qualitative research: further reflections on their integration. In: Brannen J, ed. *Mixing Methods: Qualitative and Quantitative Research*. Aldershot: Ashgate, 1992:57–78
3 Caracelli VJ, Riggin LJC. Mixed-method evaluation: developing quality criteria through concept mapping. *Eval Pract* 1994;**15**:139–52
4 Creswell JW, Fetters MD, Ivankova NV. Designing a mixed methods study in primary care. *Ann Fam Med* 2004;**2**:7–12
5 Datta L. A pragmatic basis for mixed-method designs. In: Greene JC, Caracelli VJ, eds. *Advances in Mixed-method Evaluation: The Challenges and Benefits of Integrating Diverse Paradigms*. San Francisco, CA: Jossey-Bass, 1997:33–46
6 Teddlie C, Tashakkori A. Major issues and controversies in the use of mixed methods in the social and behavioural sciences. In: Tashakkori A, Teddlie C, eds. *Handbook of Mixed Methods in Social and Behavioural Research*. London: Sage Publications, 2003:3–50
7 Sale JEM, Brazil K. A strategy to identify critical appraisal criteria for primary mixed method studies. *Quality and Quantity* 2004;**38**:351–65
8 Creswell JW, Plano-Clark V. *Designing and Conducting Mixed Methods Research*. Thousand Oaks, CA: Sage Publications, 2007
9 Brannen J. Combining qualitative and quantitative approaches: an overview. In: Brannen J, ed. *Mixing Methods: Qualitative and Quantitative Research*. Aldershot: Ashgate, 1992:3–38
10 Creswell JW. *Research Design. Qualitative, Quantitative, and Mixed Methods Approaches*. 2nd edn. London: Sage Publications, 2003
11 Mays N, Pope C, Popay J. Systematically reviewing qualitative and quantitative evidence to inform management and policy-making in the health field. *J Health Serv Res Policy* 2005;**10** (Suppl. 1):6–20
12 O'Cathain A, Murphy E, Nicholl J. Integration and publications as indicators of 'yield' from mixed methods studies. *Journal of Mixed Methods Research* 2007;**1**:147–63
13 Brewer J, Hunter A. *Multimethod Research*. London: Sage Publications, 1989
14 Tashakkori A, Teddlie C, eds. *Handbook of Mixed Methods in Social and Behavioural Research*. London: Sage, 2003
15 Tashakkori A, Teddlie C. *Mixed Methodology: Combining Qualitative and Quantitative Approaches*. London: Sage, 1998
16 Gorard S, Taylor C. *Combining Methods in Educational and Social Research*. Maidenhead: Open University Press, 2004
17 Brannen J, ed. *Mixing Methods: Qualitative and Quantitative Research*. Aldershot: Ashgate, 1992
18 Bryman A. *Quantity and Quality in Social Research*. London: Routledge, 1988
19 Greene JC, Caracelli VJ, eds. *Advances in Mixed-method Evaluation: The Challenges and Benefits of Integrating Diverse Paradigms*. San Francisco, CA: Jossey-Bass, 1997
20 Fielding NG, Fielding JL. *Linking Data*. Sage Publications, 1986
21 Murphy E, Dingwall R, Greatbatch D, Parker S, Watson P. Qualitative research methods in health technology assessment: a review of the literature. *Health Tech Assess* 1998;**2**:16
22 Mason J. Linking qualitative and quantitative data analysis. In: Bryman A, Burgess RG, eds. *Analysing Qualitative Data*. London: Routledge, 1994:89–110
23 Miller S. Impact of mixed methods and design on inference quality. In: Tashakkori A, Teddlie C, eds. *Handbook of Mixed Methods in Social and Behavioural Research*. London: Sage Publications, 2003:423–55
24 Katrak P, Bialocerkowski AE, Massy-Westropp N, Kumar VS, Grimmer KA. A systematic review of the content of critical appraisal tools. *BMC Med Res Meth* 2004;**4**:22
25 Silverman D. *Doing Qualitative Research. A Practical Handbook*. London: Sage Publications, 2000
26 Steckler A, Mcleroy KR, Goodman RM, Bird ST, McCormick L. Toward integrating qualitative and quantitative methods: an introduction. *Health Educ Q* 1992;**19**:1–8
27 Chen H. Applying mixed methods: a dominant methodology for the future? In: Greene JC, Caracelli VJ, eds. *Advances in Mixed-method Evaluation: The Challenges and Benefits of Integrating Diverse Paradigms*. San Francisco, CA: Jossey-Bass, 1997:61–72
28 Sandelowski M. Combining qualitative and quantitative sampling, data collection, and analysis techniques in mixed-method studies. *Res Nurs Health* 2000;**23**:246–55
29 O'Cathain A, Thomas KJ. "Any other comments?" Open questions on questionnaires – a bane or a bonus to research? *BMC Med Res Meth* 2004;**4**:25
30 Hammersley M. *What's Wrong with Ethnography?* London: Routledge, 1992

For peer review only


For peer review only - http://bmjopen.bmj.com/site/about/guidelines.xhtml

BMJ Open

# Implementation of national antenatal hypertension guidelines: a multi-centre multiple methods study

| | |
|---|---|
| Journal: | *BMJ Open* |
| Manuscript ID | bmjopen-2019-035762.R2 |
| Article Type: | Original research |
| Date Submitted by the Author: | 03-Sep-2020 |
| Complete List of Authors: | Whybrow, rebecca; King's College London, Women and Children's Health; Guy's and Saint Thomas' Hospitals NHS Trust, Division of Women and Children's Health<br>Webster, Louise; King's College London, Women and Children's Health<br>Girling, Joanna; Chelsea and Westminster Hospital NHS Foundation Trust<br>Brown, Heather; Brighton and Sussex University Hospitals NHS Trust<br>Wilson, Hannah; King's College London, Women and Children's Health<br>Sandall, Jane; Kings College, London, Women and Children's Health<br>Chappell, Dr Lucy; King's College London, Department of Women and Children's Health; Guy's and Saint Thomas' Hospitals NHS Trust, Division of Women and Children's Health |
| <b>Primary Subject Heading</b>: | Obstetrics and gynaecology |
| Secondary Subject Heading: | Cardiovascular medicine |
| Keywords: | Maternal medicine < OBSTETRICS, OBSTETRICS, Hypertension < CARDIOLOGY |
| | |

SCHOLARONE™
Manuscripts

*I, the Submitting Author has the right to grant and does grant on behalf of all authors of the Work (as defined in the below author licence), an exclusive licence and/or a non-exclusive licence for contributions from authors who are: i) UK Crown employees; ii) where BMJ has agreed a CC-BY licence shall apply, and/or iii) in accordance with the terms applicable for US Federal Government officers or employees acting as part of their official duties; on a worldwide, perpetual, irrevocable, royalty-free basis to BMJ Publishing Group Ltd ("BMJ") its licensees and where the relevant Journal is co-owned by BMJ to the co-owners of the Journal, to publish the Work in this journal and any other BMJ products and to exploit all rights, as set out in our licence.*

*The Submitting Author accepts and understands that any supply made under these terms is made by BMJ to the Submitting Author unless you are acting as an employee on behalf of your employer or a postgraduate student of an affiliated institution which is paying any applicable article publishing charge ("APC") for Open Access articles. Where the Submitting Author wishes to make the Work available on an Open Access basis (and intends to pay the relevant APC), the terms of reuse of such Open Access shall be governed by a Creative Commons licence – details of these licences and which Creative Commons licence will apply to this Work are set out in our licence referred to above.*

*Other than as permitted in any relevant BMJ Author's Self Archiving Policies, I confirm this Work has not been accepted for publication elsewhere, is not being considered for publication elsewhere and does not duplicate material already published. I confirm all authors consent to publication of this Work and authorise the granting of this licence.*

For peer review only

**Title:** Implementation of national antenatal hypertension guidelines: a multi-centre multiple methods study

Rebecca Whybrow[1], Louise Webster[1], Joanna Girling[2], Heather Brown[3], Hannah Wilson[1], Jane Sandall[1], Lucy Chappell[1]

1 Department of Women and Children's Health, King's College London, St Thomas' Hospital, London, UK.

2 Chelsea and Westminster Hospital NHS Foundation Trust, London, UK.

3 Brighton and Sussex Universities Hospital Trust, East Sussex, UK.

Corresponding author: Rebecca Whybrow, Department of Women and Children's Health, King's College London, St Thomas' Hospital, London, UK. Rebecca.whybrow@kcl.ac.uk Tel: 07804690276

**Word Count:** 4796

**Abstract:**

**Objective** To evaluate the implementation of NICE antenatal hypertension guidelines, to identify strategies to reduce incidences of severe hypertension and associated maternal and perinatal morbidity and mortality in pregnant women with chronic hypertension.

**Methods** We used a multiple-method multi-site approach to establish implementation of guidelines and the associated barriers and facilitators. We used a national survey of healthcare professionals (n=97), case-notes review (n=55) and structured observations (n=42) to assess implementation. The barriers and facilitators to implementation were identified from semi-structured qualitative interviews with healthcare professionals (n=13) and pregnant women (n=18) using inductive thematic analysis. The findings were integrated and evaluated using the Consolidated Framework for Implementation Research (CFIR).

**Setting and participants** Pregnant women with chronic hypertension and their principal carers (obstetricians, midwives, and physicians), at three NHS hospital trusts with different models of care.

**Results** We found severe hypertension to be prevalent (46% of case-notes reviewed) and target blood pressure practices to be sub-optimal (56% of women had an antenatal blood pressure target documented). Women were infrequently given information (52%) or offered choice (19%) regarding antihypertensives. Women (14/18) reported internal conflict in taking antihypertensives and non-adherence was prevalent (8/18). Women who were concordant with treatment recommendations described having mutual trust with professionals mediated through appropriate information, side-effect management and involvement in decision-making. Professionals reported needing updates and

For peer review only

tools for target blood pressure setting and shared decision-making underpinned by antihypertensive safety and effectiveness research.

**Conclusions** Women's nonadherence to antihypertensives is higher than anticipated. Sub-optimal information provision around treatment, choice of antihypertensives and target setting practices by healthcare professionals may be contributary. Understanding the reasons for non-adherence will inform education and decision-making strategies needed to address both clinician and women's behaviour. Further research into the effectiveness and long-term safety of common antihypertensives is also required.

**Strengths and limitations of this study**

- Multiple methodological approaches and an implementation framework improved the reliability, validity, and generalisability of the study.
- Structured observations were carried out using a validated tool with high interrater reliability.
- Women's medication behaviours were explored in-depth using a qualitative interview approach and have identified antihypertensive side-effects to be a factor of non-adherence in pregnant women.
- About two-fifths of women who participated in this study were from Black, Asian and minority ethnic groups, providing a diverse range of voices.
- Respondents to the survey were self-selecting and may represent a relatively interested group of healthcare professionals.

For peer review only

**BACKGROUND**

Hypertension in pregnancy is one of the leading causes of maternal mortality worldwide[1] and although mortality is declining in the UK,[2] women can still experience substantial morbidity from complications such as eclampsia and stroke.[3] Additionally, perinatal mortality remains high, with the UK population-attributable risk of stillbirth from chronic hypertension at 14%[4] and around half of all neonates born to mothers who have had severe hypertension in pregnancy being admitted to the neonatal unit.[5] The morbidity and mortality attributable to hypertension, in many cases, may be modifiable through optimal use of antihypertensive agents during pregnancy.

The National Institute for Health and Care Excellence (NICE) hypertension in pregnancy guidelines (2010)[6] and linked quality statements (2013)[7] contain a quality statement regarding the provision of information on the use of safe antihypertensive medication in pregnancy and has related guidance that recommends discontinuation of teratogenic medications such as angiotensin-converting-enzyme inhibitors or angiotensin II receptor blockers with prescribing of safe alternatives. Any prescribing of alternative antihypertensive medication should be dependent on pre-pregnancy treatment, side-effect profiles and teratogenicity. A second quality statement advocates that women taking antihypertensive medication should have a blood pressure target (usually of less than 150/100mmHg) set in pregnancy. All NICE guidelines are underpinned by the recommendation of enabling patients to actively participate in their care which includes adopting a shared decision-making approach to treatment decisions.[8]

Despite publication of the guideline almost a decade ago, the implementation and evaluation of associated determinants of uptake have not been nationally evaluated. As a result, targeted strategies to reduce maternal and perinatal morbidity (and mortality) resulting from severe hypertension remain unidentified. Using the Consolidated Framework for Implementation Research (CFIR), [9] the aim of the study was to evaluate the implementation of NICE hypertension in pregnancy guidelines, to identify strategies to reduce incidence of severe hypertension and associated maternal and perinatal morbidity and mortality in pregnant women with chronic hypertension. In many countries, there is a movement toward establishing consensus-driven standardised clinical guidelines with the aim of improving patient safety and clinical outcomes. Whilst new research continually emerges, guidelines are periodically updated and therefore remain an appropriate standard for evaluating routine clinical practice.[10]

For peer review only

## RESEARCH DESIGN AND METHODS

### Study setting and overall methodology

The CHAMPION study (Chronic Hypertension in pregnAncy iMPlementatION study) is a multiple methods evaluation of the implementation of the NICE hypertension in pregnancy guidelines (2010 and updated in 2013) in women with chronic hypertension diagnosed before 20 weeks.[6 7] Ethical approval for the CHAMPION study was provided by the National Research Ethics Service (17/LO/2041). The study aimed to evaluate the variability in implementation of hypertension management practices set out in the NICE hypertension in pregnancy guidelines (2010).[6] As all guidelines should be underpinned by the 'Patient experience in adult NHS services guideline'[8] which includes, actively involving patient in decisions about their care through information provision and shared decision-making, the provision of information and women's involvement in decision-making was also evaluated. The involvement of women in decision-making was considered integral to the implementation study because successful hypertension management strategies involve the adherence to, alongside the prescribing of, antihypertensive medication.

Implementation was assessed through multiple methods: an online national survey of healthcare professionals, designed to describe general trends in guideline implementation; through review of the maternity case-notes of women who had already given birth, a method that assessed the documentation of hypertension management occurrence in each woman's maternity record. Aspects of care that would not normally be documented or are more difficult to capture, such as in-consultation discussions and occurrence of shared decision-making were assessed through observations carried out by a midwife researcher (RW). The evaluation of the barriers and facilitators to implementation of NICE guidelines was assessed through qualitative interviews (with the same women and healthcare professionals who participated in the observation phase) using the Consolidated Framework for Implementation Research (CFIR). The study draws on CFIR as a theoretical framework to guide data collection, analysis, and interpretation. The CFIR framework specifically evaluates five key domains that influence implementation; each domain has several subgroups to it, although only those relevant to this study have been identified. These include the intervention characteristics (the NICE guidelines), the outer context (the pregnant women), the inner context (NHS maternity services), individual context (the healthcare professionals) and the process of implementation (potential strategies).

Implementation of guidelines was assessed between November 2017 to December 2018 at three NHS Trusts with typical configurations of services for pregnant women with hypertension in the UK. Hospital Trust 1 was a tertiary city centre hospital with a newly formed specialist service that included

For peer review only

consultant obstetricians, obstetric physicians and midwives who provided antenatal and intrapartum care to women with chronic hypertension within a specialist clinic; Hospital Trust 2 was a suburban district general hospital with a consultant-led antenatal clinic with antenatal midwives alongside providing care to women with a variety of pre-existing medical conditions; and Hospital Trust 3 had both a tertiary and a semi-rural hospital with a joint obstetric and physician led clinic and usual community-based midwifery care. No adjustment for clustering was required as no statistical comparison between sites was made. The NICE hypertension in pregnancy guidelines (2010)[6] had been adopted into local clinical guidelines at all three participating NHS Trusts for several years prior to the assessment of implementation.

**The National Survey**

The implementation of evidence-based practices for the management of hypertension in pregnancy was assessed through self-reporting using an online survey (surveygizmo/s3). We embedded questions relating to the uptake of the NICE hypertension in pregnancy guidelines (2010)[6] using the TIDieR framework.[11] The 12-item TIDieR checklist (brief name, why, what (materials), what (procedure), who provided, how, where, when and how much, tailoring, modifications, how well (planned), how well (actual) is an extension of the CONSORT 2010 statement (item 5) and the SPIRIT 2013 statement (item 11). Although the emphasis of the TIDieR checklist is on reporting interventions for trials, the checklist was used as a basis for this survey (but not as a reporting guideline) as it is also intended to apply across all evaluative study designs.[11] There is no single database of healthcare professionals' email addresses so national organisations including British Maternal and Fetal Medicine Society (BMFMS), Macdonald UK Obstetric Medicine Society (MOMS) and Royal College of Midwives (RCM) were asked to email the survey (April to September 2018) to their members. No fee was charged as members' contact details were not shared with us and as a result the response rate could not be calculated. Ninety-seven healthcare professionals from sixty-nine NHS Trusts responded, including 53 consultant obstetricians (55%), 16 doctors in training (16%), 22 specialist midwives (23%) and six community midwives (6%) (full copy of survey questions shown in supplementary material 1).

**Case-notes review**

The implementation of NICE guidelines (2010)[6] was also assessed through review of 100 maternity case-notes of women with chronic hypertension identified from the electronic maternity records (32, 33, 35 women per Trust). At two of the Trusts all women who had given birth in 2017 were included, whereas at the other Trust all women who had given birth over the final three months of 2017 were

For peer review only

included as this third Trust had approximately four times the number of women with chronic hypertension per annuum. In the UK, many women have abridged electronic maternity records and extensive handheld paper notes that are carried throughout pregnancy but are stored thereafter in the hospital. Both the electronic system and paper notes were obtained in the case-notes review of care. Due to use of varying terms for hypertension on the electronic system, some women identified for case-note review were excluded as they did not have chronic hypertension when the full case-notes were examined. Other reasons for exclusion included early miscarriage and transfer of care to another maternity unit. Data extraction based on the NICE hypertension in pregnancy guidelines (2010)[6] was completed by two midwife researchers (RW, HW), and minor discrepancies were resolved by discussion between the two researchers. It was not necessary to include a third reviewer as no major discrepancies were identified. Unclear or absent documentation including height, weight and body mass index or antenatal blood pressure recordings was recorded as missing data. Severe hypertension was defined as systolic blood pressure greater than or equal to 160 mmHg systolic or diastolic blood pressure greater than or equal to 110 mmHg. For the assessment of BP targets, the quality statement related to documentation of a target (or not), not to the specific numerical thresholds chosen.

**Observations**

Forty-two antenatal appointments involving 23 women with chronic hypertension and their respective doctors (nine) and midwives (five) were observed by a midwife researcher (RW) at the three NHS Trusts. Women with chronic hypertension were purposively sampled at their first obstetric antenatal appointment and, based on the availability of the midwife researcher, were approached consecutively along with their respective healthcare professionals until data saturation occurred. Staff and women gave written informed consent. Two women declined recruitment to the study. During observations, data about antenatal care provision were recorded using the Calgary-Cambridge communication guide[12] chosen for validity in relation to the research question, and its high interrater reliability. For example, offering choice is a sub-section of shared decision-making and is defined as “encourages patient to make choices and decisions to the level that they wish”. Attainment of each section and sub-sections was established through the analysis of all 42 appointments using descriptive statistics.

**Semi-structured interviews**

Views about barriers and facilitators to implementation of evidence-based guidelines were collected from nine doctors and four midwives who were providing antenatal care for women with chronic hypertension. The interviews were carried out by a midwife researcher (RW) following informed consent and took place in privacy away from the clinical setting. The interviews were audio

For peer review only

transcribed, coded and thematically analysed using inductive reasoning.[13] The codes generated formed small themes which were organised into the CFIR evaluation guide.[14] As formal implementation strategies had not been adopted beyond producing local guidance, interviewees were asked how they thought they could improve the implementation in the future.

Semi-structured interviews with 18 women recruited for antenatal observations were carried out in the third trimester with informed consent. Women were asked about their antenatal care experiences using an interview schedule which reflected the concepts from the International Consortium for Health Outcome Measure (ICHOM) maternity standards sets[15] which include women's overall satisfaction with their care during pregnancy; satisfaction with information provision and their relationships with their care providers (see supplementary material 2). ICHOM standards are internationally recognised measures that evaluate health outcomes that are important to patients (or pregnant women) and are used to improve local healthcare and compare outcomes internationally. The closed survey questions were turned into open ended questions to explore in-depth the quality of antenatal care provided. The interviews were carried out by a midwife researcher (RW) and took place away from the clinical setting, with assurance that discussions would not be shared with healthcare professionals and that participation or non-participation would not influence their care. The interviews were audio transcribed, coded, and thematically analysed using an inductive approach. Women's experiences were analysed to improve understanding of their antenatal care needs, which included how their hypertension was managed and the barriers and facilitators to the uptake of antihypertensives in pregnancy.

**Data analysis**

The quantitative and qualitative data were analysed separately before being integrated. Descriptive analysis and summary statistics were used for the quantitative data. The semi-structured interviews were thematically analysed by researchers (RW, JS and LC) using inductive techniques and typically lasted between 30 and 60 minutes.[16] The multiple methods data were integrated and analysed using the CFIR evaluation framework.[14] This included probing the inductively generated qualitative themes that related to implementation. The interpretation of the intervention constructs (characteristics, the inner and outer settings, the individual characteristics and the implementation processes) was carried out initially by the midwife researcher (RW) who collected the data, then with a second and third researcher (LC, JS) interpreting and discussing final interpretation of integrated data. Rigour was maintained through member reflection, attention to interview and transcription quality and systematic analysis. Rigour was improved using multiple data sources, a comprehensive integration framework (CFIR) and a multiple methods integration checklist.[17] Researchers were aware of, and

For peer review only - http://bmjopen.bmj.com/site/about/guidelines.xhtml

sensitive to, the way in which their roles as midwives and doctor may have shaped the generation and analysis of the qualitative data.

**Patient and Public Involvement**

A patient participant involvement (PPI) group consisting of women with experience of hypertension in pregnancy (n=7) and a maternity voices partnership group (n=15) provided feedback on the design of the study, research questions and outcome measures. The views of Black, Asian and minority ethnic women were purposively sought as they are disproportionately represented in the chronic hypertension in pregnancy population. PPI focus groups discussed what aspects of care were important to evaluate, this included the information women were given during pregnancy and whether women were involved in decisions about their care. They also provided constructively critical feedback on the patient information leaflets and consent forms.

**RESULTS**

Antenatal care for women with chronic hypertension was provided by consultant obstetricians and midwives at all three hospitals. In two of the hospitals, women with chronic hypertension had designated midwives attached to the obstetric clinic. Approximately one-third of those recruited to the study had a BMI over 30kg/m$^2$, approximately one-third were over the age of 35 and approximately two-fifths were of Black, Asian and minority ethnic backgrounds (shown in supplementary material 3). Hospital Trust 1 had four times the population of women with chronic hypertension compared to the other two units, comprising a large black minority ethnic population (many with associated co-morbidities). Perinatal outcomes from the fifty-five pregnancies identified for case-notes review showed that just under half of the women (46%) developed severe hypertension and that one in six babies were admitted to the neonatal unit (16%) (shown in supplementary material 4). At all three hospitals medical history of women with chronic hypertension was inaccurate in the maternity records system and episodes of severe hypertension were recorded only in hand-written notes.

**Implementation of NICE hypertension in pregnancy 2010 guidelines and 2013 quality standards**

Setting a blood pressure target (quality statement 3)

Both the survey and the case-notes review found the practice of setting an antenatal target blood pressure to be variable (table 1). Just over half of women with chronic hypertension had a target blood

For peer review only

pressure documented in maternity notes (44% did not) yet substantial variation in practice between hospitals existed. At Hospital Trust 1, 77% of women had a target blood pressure documented in pregnancy compared to 23% and 38% at Hospital Trusts 2 and 3 respectively (supplementary material 5). Whilst it is possible that undocumented discussions occurred during consultations, which could not be extracted from case-note review, such discussions would not be accessible on a longer term basis to the woman or to other healthcare professionals involved in her care. The survey results support the case-notes review findings as only a third of healthcare professional respondents reported always setting a target. The practice of undocumented 'unshared' target setting was identified through case-notes review. Evidence of blood pressure targets being used by healthcare professionals but not shared with the woman and other professionals ('unshared') was frequently found. In about three quarters of cases where the target blood pressure was unshared, and the blood pressure rose above systolic 150mmHg and or diastolic 100mmHg action was taken by professionals to lower it. Action was defined as making changes to blood pressure treatment, changing frequency of blood pressure monitoring or frequency of appointments (table 1).

Table 1. Variation in implementation of evidence-based care evaluated through a national survey of obstetricians and midwives and women's case-notes review at three representative NHS Trusts.

| **Care quality indicators** | **National Survey n=97 (%)** | **Case-notes review n=55 (%)** |
|---|---|---|
| **Blood pressure target setting (QS3)** | | |
| Target blood pressure 'always' set | 36 (37.1) | |
| Target blood pressure 'almost always' set | 36 (37.1) | |
| Target blood pressure 'never' set | 1 (1.0) | |
| Target blood pressure not applicable (midwife) | 24 (23.3) | |
| Target blood pressure set at first opportunity (whichever first: booking or commencement of AHT) | - | 9 (18.0) |
| Target blood pressure not documented | | 26 (43.6) |
| **Systolic target blood pressure** | | |
| <160mmHg | 8 (8.2) | |
| <150mmHg | 89 (91.8) | 2 (7.4) |
| ≤140mmHg | | 27 (49.0) |
| **Diastolic target blood pressure** | | |
| <100mmHg | 94 (96.9) | 2 (7.4) |
| ≤90mmHg | | 27 (49.0) |

For peer review only

| | | |
|---|---|---|
| Action taken to reduce blood pressure if above 150/100mmHg | | 13/17 (76.5) |
| **Safe antihypertensive prescribing (linked to QS1)** | | |
| **ACEi and ARBs cessation** | | |
| On ACEis or ARBs at antenatal booking appointment | | 4 (7.3) |
| Stopping ACEi or ARBs at first app if woman on either | | |
| Always | 57/86 (66.3) | - |
| Almost always | 27/86 (31.4) | - |
| ACEis or ARBs stopped at 1st obstetric appointment | | 4/4 (100.0) |
| **1st line AHT prescribing (non-exclusive)** | | |
| Labetalol | 85 (87.6) | 28 (50.9) |
| Nifedipine | 32 (33.0) | 9 (16.4) |
| Methyldopa | 29 (29.9) | 8 (14.5) |
| Other e.g. amlodipine | 2 (2.1) | 4 (7.3) |
| None | - | 6 (10.9) |
| **2nd line AHT prescribing (non-exclusive)** | | |
| Nifedipine | 79 (81.4) | 9 (16.4) |
| Methyldopa | 60 (61.9) | 4 (7.3) |
| Labetalol | 38 (39.2) | 3 (5.4) |
| Amlodipine | 37 (38.1) | 2 (3.6) |
| Doxazosin | 23 (23.7) | 0 (0.0) |
| Other | 5 (5.2) | 0 (0.0) |
| None | - | 37 (67.3) |

Antihypertensive information provision, decision-making and prescribing (quality statement 1 and associated guidance)

Variation in practice regarding first- and second-line prescribing was identified through both the notes review and survey (table 1). In both, labetalol was the most commonly prescribed first line and nifedipine the most commonly used second line antihypertensive agent; nevertheless, in about half of the case-notes reviewed labetalol was not the first line antihypertensive prescribed. First line prescribing is not always exclusive as it may vary by ethnicity (e.g. some doctors use labetalol as first line for many women, but nifedipine for Black women, in line with national guidelines for prescribing outside of pregnancy)[18] which may explain the variation in prescribing practice that existed

For peer review only - http://bmjopen.bmj.com/site/about/guidelines.xhtml

(supplementary material 5). Variation may also be explained by clinician preference or medication preference identified through shared decision-making.

Information provision about antihypertensive prescribing

Across all three Trusts, 52% (41/79) of the time the correct type and amount of information was provided during the consultation (measured using the Calgary-Cambridge Guide). Visual techniques such as drawing or using charts to provide information occurred during consultation in 14% (3/21) of cases.

Achieving a shared understanding: incorporating the woman's perspective

Of the survey respondents 96.9% strongly agreed or agreed that involving women with chronic hypertension in management plans during pregnancy was important. However, when asked to give examples of how they involve women, only 4.3% identified discussing risks and benefits of treatment choice and 10% of respondents identified that women could be involved in plans about antihypertensive prescribing. The observations in the three hospital trusts found that 43% of the time (41/96) shared decision-making occurred and 19% of women (3/16) were offered a choice regarding their hypertensive plans (including choice of antihypertensive).

**Barriers and Facilitators to implementation (CFIR)**

Intervention characteristics (evidence and guideline)

All professionals interviewed, except one, saw value in having national guidance and understood that the local guidelines had been adapted from the 2010 national guideline.[6] Midwives relied more on local guidelines compared to obstetricians who referred more commonly to NICE guidelines. Some of the medical professionals had been involved in the development of a NICE guideline and were aware of the strengths and limitations of producing evidence-based guidelines in terms of the need for timely updating. Professionals described difficulties in creating guidelines where there is a paucity of robust data as is sometimes the case in maternity care. Weak, out of date or absent evidence influenced doctors' decisions not to implement guidelines. Some doctors described the weaknesses in the evidence underpinning the hypertension guidelines and described relying more on recent research compared to older national guidelines (table 2). The professionals identified that further research is necessary to support evidenced-based national guidelines (figure 1).

For peer review only

Table 2. Barriers to healthcare professional's implementation of hypertension in pregnancy guidelines, based on Consolidated Framework for Implementation Research (CFIR) implementation themes.

| **CFIR implementation themes** | **Frequency** | **Codes** | **Representative answer** |
|---|---|---|---|
| **Intervention characteristics** | | | |
| Evidence strength, quality, source, and adaptability | 17 | AHT prescribing; target setting; | - "I think the fact that it says use labetalol first line is not what we do, I don't believe the evidence for labetalol being better than methyldopa is there."[H]<br>- "we can't get away from the fact that there aren't the source data there to make evidence-based guidelines."[B]<br>- So, I kept a close track of what was happening with the CHIPS study…I got a lot of information and knowledge from it."[A] |
| **Inner setting** | | | |
| Structural characteristics | 43 | Information provision; pathways and models; training and education; time | - "I don't think we have a hand-out for, to give to hypertensive women about hypertension in pregnancy"[L]<br>- "we don't have a dedicated hypertension clinic here. So, most of these women will get seen in general antenatal clinic"[I]<br>- "you have people coming in three times weekly or something for their blood pressure, really? And other people who perhaps aren't being seen enough"[I] |
| Relative priority | 26 | Guidelines; self-study; beliefs; experience; | - "Well actually I don't even know what the NICE guidelines are for hypertension, I'm not a… as my colleagues will tell you, not a huge fan of NICE, in many ways."[L]<br>- "I'm not just interested in guidelines; I'm interested in people's clinical experience…and that feel."[C] |

For peer review only

| | | | |
|---|---|---|---|
| Culture of decision-making | 19 | Patriarchy; shared decision-making; type of decision: emergency, urgent and non-urgent | - “Doctors… see it as patients not doing what they’re told”[A]<br>- “I think that there’s a balance to be had between involving women in the decisions, versus, them coming for expert recommendations”[F]<br>- “If I have a clinical situation where I want to start antihypertensives because she’s got a dangerously high blood pressure, then that discussion is inevitably truncated.” [B] |
| **Individual characteristic** | | | |
| Beliefs about the intervention | 35 | AHT medication; AHT safety and side-effects; target setting | - “National guidelines do not sanction any particular antihypertensive, or that the, the drug licenses do not sanction any particular antihypertensive”[B]<br>- “I think that might be something we’re not quite as good at as we should be about defining a target for women….I suspect it’s something we don’t really document and clarify”[H] |
| Self-efficacy | 17 | Women’s concordance/ desire for involvement/ first language | - “I think sometimes women don’t necessarily want to make the decision”[D]<br>- “There’s a lot of ‘mumsnet’….and I would say they take a, that advice just as seriously as they do the advice that we give them here.”[C] |
| **Process of implementation** | | | |
| Engaging people and process of implementation | 16 | Using guidelines; updates, toolkits, and information; shared decision-making | - “Awareness for people, if you’re a busy jobbing healthcare practitioner, keeping up to date with each new area”[H]<br>- “Practical toolkits to help with that consultation”[B]<br>- Evidenced based information having it more readily available for patient”[D] |

For peer review only

| | | | |
|---|---|---|---|
| Opinion leaders; Champions; | 5 | Utilisation of opinion leaders/ champions in implementation | - "I find as a midwife sometimes you're a bit powerless, you know what the guidelines are, but depending on the doctor you're working with, tends to be the influencing factor on the decisions that are made… so it seems to be clinician-based guidelines sometimes, rather than the trust or national guidelines"[D1] |

For peer review only

[1] Letters [A-M] represent the healthcare professionals interviewed

Inner setting (organisation structure and culture)

The most frequently cited barriers to implementing high quality care for women with chronic hypertension were linked to the structure and organisation of antenatal care. Interviewees reported that a lack of consensus and guidance exists relating to models of care (such as whether specialist services would improve outcomes through better implementation) and pathways of care (such as frequency of blood pressure and medication reviews) (table 2). Evidence-based recommendations on models, and pathways of care, were identified as future facilitators to providing optimal antenatal care (figure 1). Whilst most healthcare professionals initially described the uptake of the guidelines as a clinical priority during the interviews, clinicians identified difficulty with keeping up with recommendations and using them alongside clinical judgement as barriers to implementation (table 2).

Healthcare professionals considered the absence of written information a barrier to the uptake of antihypertensives in women with hypertension (table 2). A degree of paternalism exists in relation to involving women in decisions about their care. In principle, clinicians would like to involve women in decision-making, yet they gave many examples of situations where they would exercise restraint in doing so (table 2). Education and tools to support shared decision-making were identified as facilitators to optimizing antenatal care for women with hypertension (figure 1).

Characteristics of individuals (beliefs, knowledge, and self-efficacy)

Interview analysis identified doctors' and midwives' knowledge and beliefs as the second most frequently cited barrier and facilitator to the implementation of hypertension management guidelines (table 2). There existed confusion about whether the guidelines sanction one antihypertensive medication over another for the management of chronic hypertension and if so, what evidence was used to support this. Likewise, confusion about blood pressure targets was described frequently as outcomes from a recent randomised controlled trial superseded the pre-dated national guidelines (table 2). Whilst midwives experienced less self-efficacy than the doctors, doctors still experienced difficulties in this area. They occasionally described the women's beliefs and views as a barrier to implementing the recommendations (table 2).

For peer review only

336 Outer setting (women's views and experiences)

337 The quality of antenatal care experience was affected by women's internal conflict. There was also a high degree of variability in medication adherence (defined as, a blanket term factoring the extent to which patients' drug dosing histories conform, or not, to their corresponding prescribed drug dosing regimen).[19] and concordance (defined as, an agreement after negotiation between a woman and a healthcare professional that respects the beliefs and wishes of the woman in determining whether, when, and how medicines are to be taken).[20] Analysis identified that women require quality information about antihypertensives and their side-effects, blood pressure ranges in pregnancy, as well as support to actively participate in decision-making.

345

346 *Internal Conflict*

347 The majority (14 of 18) of women experienced internal conflict relating to the management of their hypertension during pregnancy, defined as a state of uncertainty about the course of action to take often in relation to making choices involving risk or uncertainty of outcomes (8) (figure 2a). The causes of internal conflict were identified as a lack of information provision, poorly managed side-effects, women's personal beliefs and factors relating to the healthcare professional (table 3).

352

For peer review only

Table 3. Barriers to women's uptake of hypertension in pregnancy guidelines

| **CFIR outer context themes - Women's internal conflict** | **Frequency** | **Codes** | **Representative answer** |
|---|---|---|---|
| Information | 30 | Medication (choices, dose, effectiveness, safety, interactions); severity of HTN; effect of HTN on pregnancy | - "[I wanted to know] how safe it is, about the dosage, about the, taking the med-, this medication, about the side-effects and so and so and so, if they think any other option for me, or if this medication is not working, what will be the other option for me"[J]<br>- "He was, you still need to carry on with your ramipril. I know I can't take it. It says in the leaflet not to take once you've hit 6 weeks, you need to stop. So, he was like oh, and then he phoned here, and he said oh well just take what you took before"[H] |
| Side-effects | 21 | Maternal side-effects; fetal side-effects; Interactions ; allergies; choices | - "They gave me first three, twice a day, then I was so giddy where I couldn't, if I take, I had to sleep all day for two days…Then I complained, but they still say to still take tablet."[I]<br>- "I'm on 18 pills a day, I do worry a bit about how they kind of potentially interact with each other and affect the baby"[F] |

| | | | |
|---|---|---|---|
| Beliefs | 17 | Hypertension status; understanding HTN; effectiveness AHT; safety AHT | - "I felt like I had to justify why I wasn't taking my tablet, which to me didn't seem right, 'cause if it, if my blood pressure was normal, and I took a tablet, surely my blood pressure then would be low?"[Q]<br>- "cause everything I take my baby takes. So, it's like, what happens if my child comes out and then they're addicted to something, or they're high-strung because of something, or they're really moody and they're crying all the time because of the medicine I've had to take for the past 4 months"[L] |
| HCP factors | 17 | Continuity; listening to women; explaining regimes, mutual trust; communication | - "My issue has been where I've seen somebody who doesn't know the history, and typically they are a more junior doctor, and typically they are ticking a box and following a flow chart….the doctor said, you know, we're going to come to an agreement together but there was absolutely no discussion, she had no interest in what I had to say."[K] |
| External factors | 7 | Family and friends; internet; access to services | - "My dad had been on beta blockers, which is what labetalol is, when he was younger, and he found, he was very ill on them, so he gave me a really negative impression of them"[P2] |

[2] Letters [A-R] represent the pregnant women interviewed

For peer review only

*Concordance*

All women identified as concordant with healthcare professional management plans described being adherent to their antihypertensives. Facilitators to concordance included trust in the healthcare professional, mediated through information about safety of antihypertensives in pregnancy, knowledge about target blood pressure in pregnancy hypertension, acknowledgement of medication side-effects and a positive interaction with the healthcare professional (including communication and approach to decision-making) (figure 2b).

*Adherence*

Internal conflict was an important determinant of non-adherence (figure 2a) as only the women who expressed internal conflict reported non-adherence to antihypertensive medication. Around half (8 of 18) the women interviewed described non-adherence to prescribed antihypertensives at some point during pregnancy with three women non-adherent at the time of interview (third trimester). However, nine of 14 women describing internal conflict were adherent at the time of interview which was mediated by the 'responsibility of motherhood' rather than concordance with the hypertension management plan (figure 2b).

Process of implementation (implementation strategies)

All three Trusts had a consultant obstetrician who led the care of women with chronic hypertension and could be considered the opinion leader. Two of three Trusts had a named midwife or team of midwives who specialised in the care of these women and were potential champions. However, influencers and champions were not always utilised to support guideline implementation. Further, as implementation of the guidelines had not been audited in any of the Trusts, although some outcome data was routinely collected and analysed, opportunities to address unwanted variability were being missed. These findings are supported by the national survey which found only a quarter of the Trusts collected and analysed the outcomes of women with chronic hypertension in pregnancy.

For peer review only

**DISCUSSION**

Women in this study (14/18) reported conflict relating to the uptake of prescribed antihypertensives in pregnancy and in many cases (8/14) internal conflict resulted in non-adherence. The most commonly cited reasons for conflict were lack of information provision, the side-effects experienced from the medication, beliefs about safety of medication and uncertainty about normal blood pressure ranges in pregnancy. Adherence to antihypertensives in conflicted pregnant women was mediated through a responsibility to motherhood rather than through a trusting partnership with healthcare professionals (supported by information provision, management of side-effects and relational factors) as found in concordant adherent women. Despite this, our findings demonstrated that optimal information provision about antihypertensives and shared decision-making occurred infrequently during antenatal consultations. Our findings also illustrated that the implementation of blood pressure target setting was sub-optimal as a result of 'unshared' or undocumented target setting and in some cases an absence of target setting.

A major strength of the study is the recruitment of Black, Asian and minority ethnic women to both the research (40%) and in the PPI planning stage as these women are disproportionally represented in the chronic hypertension in pregnancy population. A further strength is the use of multiple methodological approaches and an implementation framework in order to improve reliability, validity and generalisability. However, results from the national survey may overstate compliance with national guidance. The survey was sent out to healthcare professionals from professional organisations; respondents were therefore self-selecting and may represent a relatively interested group of healthcare professionals. The non-response rate is also unknown. The structured observations were carried out using a validated tool with high interrater reliability.[12] However, the observations were carried out by one midwife researcher which may affect the validity of the findings. Finally, the purposive sampling of healthcare professionals providing routine antenatal care for women with chronic hypertension resulted in a focus on lead carers (consultant obstetricians, obstetric medicine specialists and named midwives) being interviewed, rather than doctors in training and midwives in acute areas such as the maternity assessment unit.

The emergence of implementation science in recent years has identified that a gap between research findings and clinical practice exists, and that clinical guideline production does not ensure evidence-based practices are routinely adopted.[21] A recent study in British Colombia evaluated the implementation of recently published pregnancy hypertension guidelines and its associated effect on maternal and perinatal outcomes.[22] Following guideline dissemination the study reported a fall of

For peer review only

about a third in combined adverse maternal health outcomes (3.1% to 1.9%) but did not report a significant reduction in adverse perinatal outcomes.[22] However, the wanted and unwanted variability in guidance uptake was not reported and the underlying mechanisms that influenced outcomes is not described. Our study uses an implementation framework by which variability in the implementation of existing guidelines could be described and mechanisms that support and hinder their uptake can be analysed, uniquely identifying strategies to improve the uptake of guidance and reduce maternal and fetal morbidity. Critically, although the NICE hypertension in pregnancy guidelines[6] have been recently updated, the core hypertension management recommendations remain unchanged, as do the quality statements. Therefore, the findings of this study remain important and relevant to those wanting to improve implementation.

The study also adds to the small body of antihypertensive adherence in pregnancy research that has found antihypertensive side-effects are a determinant of non-adherence. One recent randomised controlled trial identified 11% of those included in randomisation discontinued the antihypertensive due to side-effects.[23] Through the qualitative interview approach that enabled in depth exploration of women's medication behaviours, our study found about 40% of all women did not adhere to their prescribed antihypertensives at some point during pregnancy. This number compared more similarly to an internet-based study of 210 pregnant women undertaken in Europe, America and Australia which identified a 32.9% non-adherence rate in women taking cardiovascular medications in pregnancy.[24] These findings are supported by similar smaller questionnaire-based studies of pregnant women's medication adherence. [25 26] Our study may have identified higher rates of non-adherence due to the nature of qualitative interviewing that explore in-depth women's experiences and therefore unpick medication behaviours in a way that quantitative studies cannot.

Women's adherence to antihypertensives in pregnancy was found to be sub-optimal, and strategies to improve adherence are likely to reduce incidences of severe hypertension and prevent associated morbidity (and mortality). [27] These include improved information provision about anti-hypertensives and blood pressure targets as well as embedding shared decision-making into practice. Improvements in target blood pressure setting practices overall are also likely to reduce incidences of severe hypertension and prevent associated morbidity (and mortality). [3 5]

This study adds to the body of research that already exists outside of pregnancy which demonstrates that implementation of guidelines is not optimally achieved through the process of diffusion.[21] Although there was some evidence that some aspects of implementation were improved by having a specialist service for hypertension, this is likely to be most easily justified in areas where there is a high prevalence of chronic hypertension. Therefore, strategies to improve implementation in wider

For peer review only

settings are required. Professionals require guideline updates, implementation toolkits (to improve target blood pressure setting practices, standardised information about antihypertensives and in consultation aids) as well as support to have better conversations with their patients about medication choices and to improve the involvement of the women in the decision-making. Professionals also need to buy into the evidence that underpins the guidance. Maternal and perinatal outcomes, which includes episodes of severe hypertension, should be collected annually, and used to support informed discussions about optimising antenatal care for this group of women.

Further research into the effectiveness and long-term safety of common antihypertensives in pregnancy and breastfeeding to support evidenced-based guidelines is required.[28] Future research may also wish to evaluate strategies to reduce women's conflict regarding their antihypertensive use in pregnancy and establish the effect of interventions on maternal concordance and health outcomes. However, without further evidence relating to the safety and effectiveness of common antihypertensives it is unclear if further reductions in maternal and fetal morbidity can be achieved through prescribing practices. Future research should also focus on active implementation of blood pressure target setting and pathways for those with outside of target blood pressure readings. This is likely to reduce morbidity as target blood pressure setting in pregnancy has been shown to reduce incidences of severe hypertension.[3 5] Policymakers may also wish to consider further studies that identify effective models and pathways of care for reducing adverse perinatal outcomes within the context of pregnancy hypertension.

**CONCLUSION**

Maternal and neonatal morbidity resulting from severe hypertension in pregnancy is prevalent. [1 4 5] This evaluation of the implementation of the NICE hypertension in pregnancy guidelines (2010)[6] addresses strategies to reduce the number of episodes of severe hypertension and has identified sub-optimal target setting practices, poor information provision for pregnant women and variability in prescribing practices. Women's non-adherence to antihypertensives is higher than previously reported and this is likely to be contributing to adverse perinatal outcomes. Analysis of the domains that influence implementation of the guidelines have identified that education and decision-making strategies are needed to address both clinician and women's behaviour. Further research into the effectiveness and long-term safety of common antihypertensives is also required.

a. Contributor statement – RW, LC and JS conceived of the study, the manuscript, and analyses, with contributions from LW, JG, HB and HW. RW was responsible for data management and data analysis. All authors reviewed, critically revised, and approved the manuscript.

For peer review only

b. Competing interests – None declared

c. Funding – This work was supported by the National Institute for Health Research (Research Professorship RP-2014-05-019) and by the National Institute for Health Research (NIHR) Collaboration for Leadership in Applied Health Research and Care South London (NIHR CLAHRC South London) at King’s College Hospital NHS Foundation Trust. Jane Sandall is an NIHR Senior Investigator and is supported by the National Institute for Health Research (NIHR) Applied Research Collaboration South London (NIHR ARC South London) at King’s College Hospital NHS Foundation Trust. The views expressed are those of the author[s] and not necessarily those of the NIHR or the Department of Health and Social Care.

d. Data sharing statement - All data relevant to the study are included in the article.

For peer review only

References

1. Bramham K, Parnell B, Nelson-Piercy C, et al. Chronic hypertension and pregnancy outcomes: systematic review and meta-analysis. *BMJ : British Medical Journal* 2014;348:g2301. doi: 10.1136/bmj.g2301

2. Knight M NM, Tuffnell D, Kenyon S, Shakespeare J, Brocklehurst P, Kurinczuk JJ (Eds.) on behalf of MBRRACE-UK. . Saving Lives, Improving Mothers' Care – Surveillance of maternal deaths in the UK 2012−14 and lessons learned to inform maternity care from the UK and Ireland Confidential Enquiries into Maternal Deaths and Morbidity 2009-14, 2016.

3. Magee LA, von Dadelszen P, Rey E, et al. Less-tight versus tight control of hypertension in pregnancy. *N Engl J Med* 2015;372(5):407-17. doi: 10.1056/NEJMoa1404595 [published Online First: 2015/01/30]

4. Flenady V, Koopmans L, Middleton P, et al. Major risk factors for stillbirth in high-income countries: a systematic review and meta-analysis. *Lancet* 2011;377(9774):1331-40. doi: 10.1016/S0140-6736(10)62233-7 [published Online First: 2011/04/19]

5. Magee LA, von Dadelszen P, Singer J, et al. The CHIPS Randomized Controlled Trial (Control of Hypertension in Pregnancy Study): Is Severe Hypertension Just an Elevated Blood Pressure? *Hypertension* 2016;68(5):1153-59. doi: 10.1161/HYPERTENSIONAHA.116.07862 [published Online First: 2016/09/14]

6. (NICE) NIfHCE. Hypertension in pregnancy CG107 2010 [Available from: https://www.nice.org.uk/guidance/cg107

7. (NICE) NIfHCE. Hypertension in pregnancy. Quality Standard (QS35), 2013.

8. (NICE) NIfHCe. Patient Experience in Adult NHS Services: Improving the Experience of Care for People Using Adult NHS Services: Patient Experience in Generic Terms. London2012.

9. Kirk MA, Kelley C, Yankey N, et al. A systematic review of the use of the Consolidated Framework for Implementation Research. *Implementation Science* 2016;11(1):72. doi: 10.1186/s13012-016-0437-z

10. Kirkpatrick DH, Burkman RT. Does Standardization of Care Through Clinical Guidelines Improve Outcomes and Reduce Medical Liability? *Obstetrics & Gynecology* 2010;116(5):1022-26. doi: 10.1097/AOG.0b013e3181f97c62

11. Hoffmann TC, Glasziou PP, Boutron I, et al. Better reporting of interventions: template for intervention description and replication (TIDieR) checklist and guide. *BMJ : British Medical Journal* 2014;348:g1687. doi: 10.1136/bmj.g1687

12. Burt J, Abel G, Elmore N, et al. Assessing communication quality of consultations in primary care: initial reliability of the Global Consultation Rating Scale, based on the Calgary-Cambridge Guide to the Medical Interview. *BMJ Open* 2014;4(3):e004339. doi: 10.1136/bmjopen-2013-004339

13. Braun V, Clarke V. Using thematic analysis in psychology. *Qualitative Research in Psychology* 2006;3(2):77-101. doi: 10.1191/1478088706qp063oa

14. Damschroder LJ, Aron DC, Keith RE, et al. Fostering implementation of health services research findings into practice: a consolidated framework for advancing implementation science. *Implementation science : IS* 2009;4:50. doi: 10.1186/1748-5908-4-50 [published Online First: 2009/08/12]

15. International consortium for health outcome measurements i. Pregnancy and childbirth standard set. 2016

16. Pope C, Ziebland S, Mays N. Qualitative research in health care. Analysing qualitative data. *BMJ* 2000;320(7227):114-16. doi: 10.1136/bmj.320.7227.114

17. O'Cathain A, Murphy E, Nicholl J. The quality of mixed methods studies in health services research. *Journal of health services research & policy* 2008;13(2):92-8. doi: 10.1258/jhsrp.2007.007074 [published Online First: 2008/04/18]

For peer review only

18. excellence Nifhac. Hypertension in adults: diagnosis and management (NG136). 2019

19. Vrijens B, Vincze G, Kristanto P, et al. Adherence to prescribed antihypertensive drug treatments: longitudinal study of electronically compiled dosing histories. *BMJ* 2008;336(7653):1114-17. doi: 10.1136/bmj.39553.670231.25

20. Dickinson D, Wilkie P, Harris M. Taking medicines: concordance is not compliance. *BMJ* 1999;319(7212):787. doi: 10.1136/bmj.319.7212.787

21. Rangachari P, Rissing P, Rethemeyer K. Awareness of evidence-based practices alone does not translate to implementation: insights from implementation research. *Quality management in health care* 2013;22(2):117-25. doi: 10.1097/QMH.0b013e31828bc21d [published Online First: 2013/04/02]

22. von Dadelszen P, Sawchuck D, McMaster R, et al. The Active Implementation of Pregnancy Hypertension Guidelines in British Columbia. *Obstetrics & Gynecology* 2010;116(3):659-66. doi: 10.1097/AOG.0b013e3181eb669d

23. Webster Louise M, Myers Jenny E, Nelson-Piercy C, et al. Labetalol Versus Nifedipine as Antihypertensive Treatment for Chronic Hypertension in Pregnancy. *Hypertension* 2017;70(5):915-22. doi: 10.1161/HYPERTENSIONAHA.117.09972

24. Lupattelli A, Spigset O, Nordeng H. Adherence to medication for chronic disorders during pregnancy: results from a multinational study. *International Journal of Clinical Pharmacy* 2014;36(1):145-53. doi: 10.1007/s11096-013-9864-y

25. Matsui D. Adherence with Drug Therapy in Pregnancy. *Obstetrics and Gynecology International* 2012;2012:5. doi: 10.1155/2012/796590

26. Abheiden CNH, van Reuler AVR, Fuijkschot WW, et al. Aspirin adherence during high-risk pregnancies, a questionnaire study. *Pregnancy Hypertension: An International Journal of Women's Cardiovascular Health* 2016;6(4):350-55. doi: https://doi.org/10.1016/j.preghy.2016.08.232

27. Abalos E, Duley L, Steyn DW, et al. Antihypertensive drug therapy for mild to moderate hypertension during pregnancy. *Cochrane Database Syst Rev* 2018;10:CD002252. doi: 10.1002/14651858.CD002252.pub4 [published Online First: 2018/10/03]

28. Excellence NIfHaC. Hypertension in pregnancy: diagnosis and management

NICE guideline [NG133]. 2019

Figure 1. Interpretation of integrated analysis: a strategy for improved implementation of evidence-based hypertension in pregnancy management

Figure 2a. Women's adherence and concordance with prescribed antihypertensives. Numbers 1-18 represent interviewed women and their experiences of anti-hypertensive prescribing during pregnancy. Women who experienced a change in their adherence or in the reporting of internal conflict are plotted more than once in different bubbles. 2b. Facilitators of women's adherence and of concordance.

For peer review only

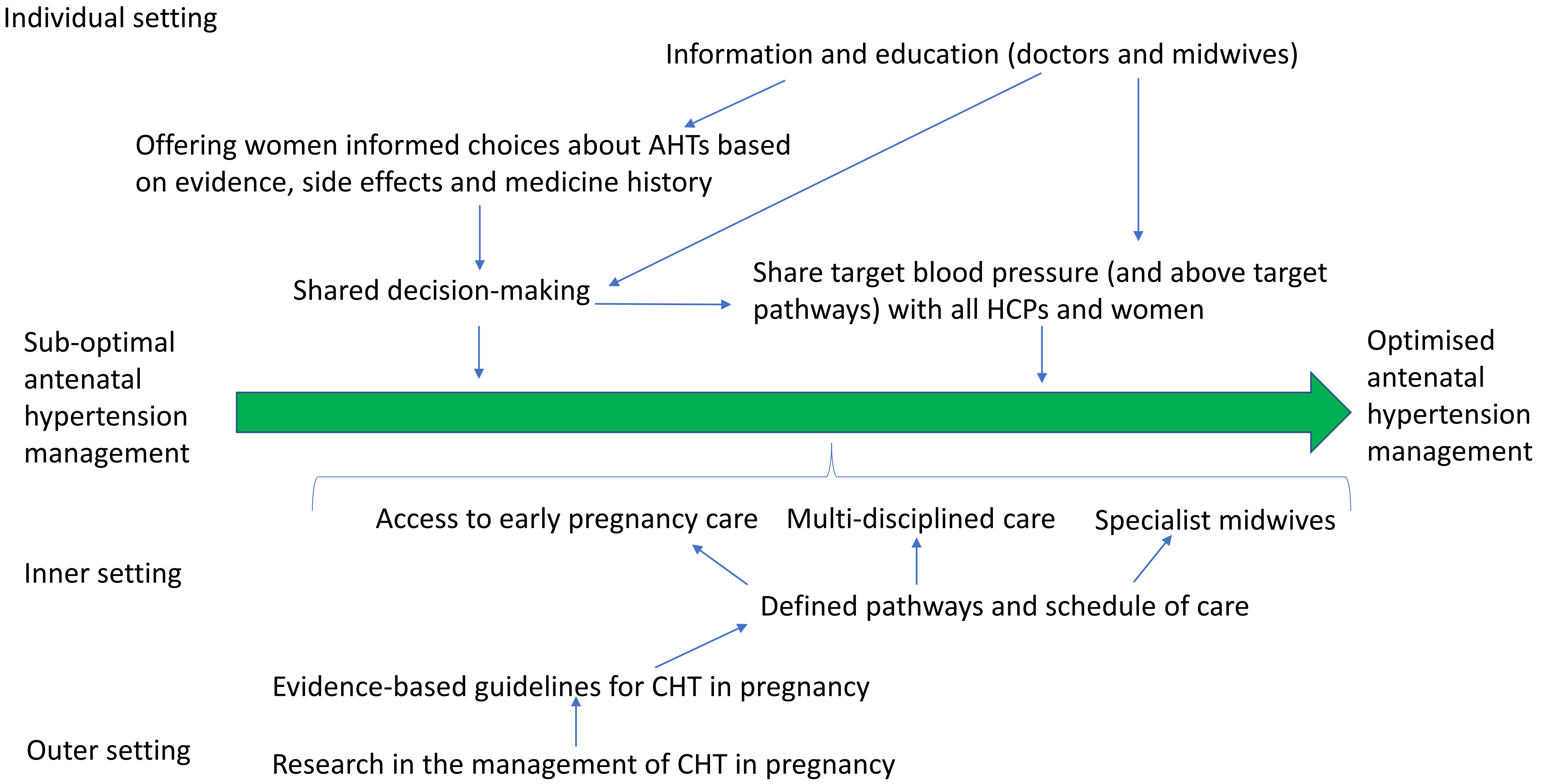
Individual setting
Information and education (doctors and midwives)
Offering women informed choices about AHTs based on evidence, side effects and medicine history
Shared decision-making
Share target blood pressure (and above target pathways) with all HCPs and women
Sub-optimal antenatal hypertension management
Optimised antenatal hypertension management
Access to early pregnancy care
Multi-disciplined care
Specialist midwives
Inner setting
Defined pathways and schedule of care
Evidence-based guidelines for CHT in pregnancy
Outer setting
Research in the management of CHT in pregnancy

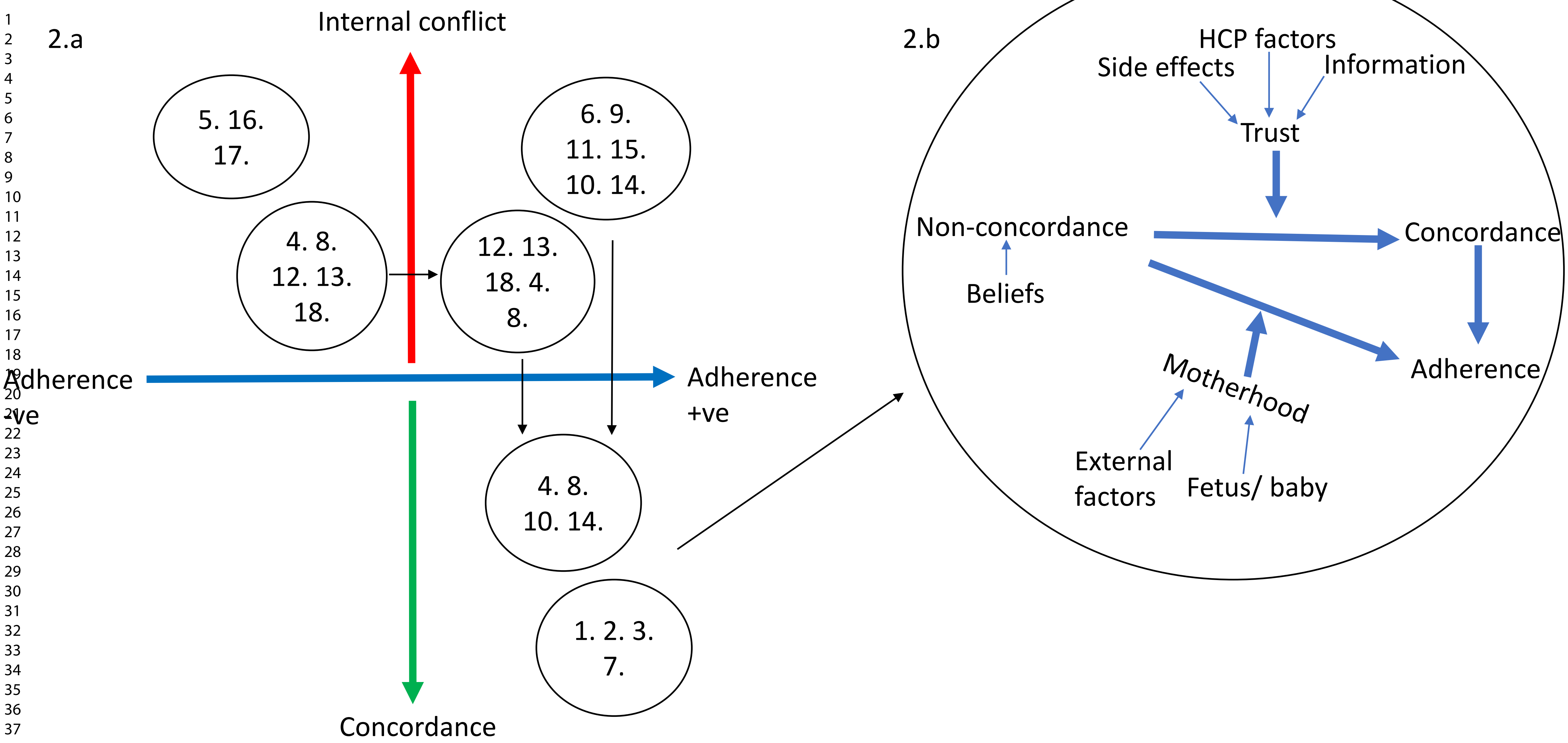
2.a
Internal conflict
5. 16. 17.
6. 9. 11. 15. 10. 14.
4. 8. 12. 13. 18.
12. 13. 18. 4. 8.
Adherence -ve
Adherence +ve
4. 8. 10. 14.
1. 2. 3. 7.
Concordance
2.b
HCP factors
Side effects
Information
Trust
Non-concordance
Concordance
Beliefs
Adherence
Motherhood
External factors
Fetus/ baby

Supplementary file 1

Chronic hypertension in pregnancy – healthcare professional survey

| Respondents | Number (97) | Percentage % |
|---|---|---|
| Obstetrician<br>Of which are consultants | 69<br>53 | 71.1<br>55 |
| Midwife<br>Of which are specialist/ senior midwife | 28<br>22 | 28.9<br>22.7 |
| NHS hospital trusts represented<br>(including England, Northern Ireland, Scotland and Wales) | 69 | - |

Question 1: If you see a pregnant woman with chronic hypertension who is currently taking either ACEIs or ARBs (e.g. at the beginning of pregnancy), how often would you ask her to stop taking them?

| Response | Number (97) | Percentage (%) |
|---|---|---|
| Always | 57 | 57.8 |
| Almost always | 27 | 27.8 |
| About two thirds of the time | 1 | 1 |
| About half of the time | 4 | 4.1 |
| About a third of the time | 0 | 0 |
| Very rarely | 1 | 1 |
| Never | 3 | 3.1 |
| Missing | 4 | 4.1 |

Question 2: What do you usually use as your first line anti-hypertensive treatment(s) for women with chronic hypertension in pregnancy?

| Anti-hypertensive (non-exclusive) | Number (97) | Percentage (%) |
|---|---|---|
| Labetalol | 85 | 87.6 |
| Methyldopa | 29 | 29.9 |
| Nifedipine | 32 | 33.0 |
| Amlodipine | 2 | 2.1 |

Question 3: What additional anti-hypertensive medication do you use for treating women with chronic hypertension in pregnancy?

| Anti-hypertensive (non-exclusive) | Number (97) | Percentage (%) |
|---|---|---|
| Amlodipine | 37 | 38.1 |
| Atenolol | 2 | 2.1 |
| Doxazosin | 23 | 23.7 |
| Enalapril | 1 | 1.0 |
| Hydralazine (oral) | 2 | 2.1 |
| Labetalol | 38 | 39.2 |
| Methyldopa | 60 | 61.9 |
| Metoprolol | 1 | 1.0 |
| Nifedipine | 79 | 81.4 |

For peer review only

Question 4: How frequently do you set a blood pressure target for women with chronic hypertension in pregnancy who need anti-hypertensive treatment (assuming no other co-morbidity) (mmHg)?

| **Answer** | **Number (97)** | **Percentage %** |
|---|---|---|
| Always | 36 | 37.1 |
| Almost always | 36 | 37.1 |
| About two thirds of the time | 8 | 8.2 |
| About half of the time | 3 | 3.1 |
| About a third of the time | 4 | 4.1 |
| Very rarely | 3 | 3.1 |
| Never | 1 | 1.0 |
| Other<br>In the guidelines but compliance unknown<br>Frequency not described | 6<br>2<br>4 | 6.2 |

Question 5: What blood pressure target do you usually set for pregnant women with chronic hypertension (assuming no other co-morbidity) (mmHg)?

| **Systolic** | **Number (97)** | **Percentage %** | **Median (IQR1-IQR3)** |
|---|---|---|---|
| 120 | 2 | 2.1 | |
| 125 | 0 | 0.0 | |
| 130 | 6 | 6.2 | |
| 135 | 2 | 2.1 | |
| 140 | 33 | 34.0 | |
| 145 | 0 | 0.0 | |
| 150 | 40 | 41.2 | |
| 155 | 1 | 1.0 | |
| 160 | 8 | 8.2 | |
| Missing | 4 | 4.1 | |
| Median | | | 150 (140-150) |

| **Diastolic** | **Number (97)** | **Percentage %** | **Median (IQR1-IQR3)** |
|---|---|---|---|
| 80 | 9 | 9.3 | |
| 85 | 7 | 7.2 | |
| 90 | 37 | 38.1 | |
| 95 | 8 | 8.2 | |
| 100 | 27 | 27.8 | |
| 110 | 3 | 3.1 | |
| Missing | 5 | 5.2 | |
| Median | | | 90 (90-100) |

For peer review only

Question 6: How often do you prescribe Aspirin for women with chronic hypertension in pregnancy?

| Answer | Number (97) | Percentage % |
|---|---|---|
| Always | 53 | 54.6 |
| Almost always | 36 | 37.1 |
| About two thirds of the time | 5 | 5.2 |
| About half of the time | 2 | 2.1 |
| Very rarely | 1 | 1.0 |

Question 7: At what gestation do these women usually receive their first Aspirin prescription?

| Answer | Number (97) | Percentage % |
|---|---|---|
| Before 12 weeks | 41 | 42.3 |
| 12-15+6 weeks | 52 | 53.6 |
| 16-19+6 weeks | 1 | 1.0 |
| Missing answer | 3 | 3.1 |

Question 8: For a woman with uncomplicated chronic hypertension in pregnancy (i.e. no additional risk factors), how many routine fetal growth scans do they receive (excluding nuchal and anomaly scans)?

| Additional scans | Number (97) | Percentage % | Median (IQR1-IQR3) |
|---|---|---|---|
| None | 4 | 4.1 | |
| 1 | 12 | 12.4 | |
| 2 | 23 | 23.7 | |
| 3 | 37 | 38.1 | |
| 4 | 21 | 21.6 | |
| >4 | 1 | 1.0 | |
| | | | 3 (2-3) |

Question 9: When do you usually plan birth for women with chronic hypertension whose blood pressure is controlled below 160/110?

| Gestation | Number (97) | Percentage (%) | Median (IQR1-IQR3) |
|---|---|---|---|
| Before 34 weeks | 3 | 3.1 | |
| 34-34+6 weeks | 2 | 2.1 | |
| 35-35+6 weeks | 2 | 2.1 | |
| 36-36+6 weeks | 4 | 4.1 | |
| 37-37+6 weeks | 27 | 27.8 | |
| 38-38+6 weeks | 36 | 37.1 | |
| 39-39+6 weeks | 41 | 42.3 | |
| 40-41 weeks | 28 | 28.9 | |
| Await spontaneous labour | 5 | 5.2 | |
| Other – individualised | 4 | 4.2 | |
| | | | 38.5<br>(37-39) |

For peer review only

Question 10: Involving pregnant women who have chronic hypertension in their pregnancy and birth planning is an important part of the consultation?

| **Sentiment** | **Number (97)** | **Percentage (%)** |
|---|---|---|
| Agree Strongly | 79 | 81.4 |
| Agree | 15 | 15.5 |
| Slightly Agree | 2 | 2.1 |
| Slightly disagree | 0 | 0.0 |
| Disagree | 0 | 0.0 |
| Disagree Strongly | 1 | 1.0 |

Question 11: If you wish, can you give an example of how you enable women to be actively involved in their care?

| **Themes** | **Number (47)** | **Percentage %** |
|---|---|---|
| Total responses | 47 | |
| SDM in the following areas | | |
| • Home BP | 10 | 21 |
| • Monitoring BP | 6 | 12.8 |
| • Anti-hypertensives | 5 | 10.6 |
| • Planning birth (induction of labour) | 17 | 36 |
| • Organisation of care | 4 | 8.5 |
| Discussing risks and benefits | 2 | 4.3 |
| How to identify pre-eclampsia | 2 | 4.3 |

Question 12: In your maternity unit what term/s best describes the antenatal care provided to most women with chronic hypertension?

| **Care provision** | **Number (97)** | **Percentage %** |
|---|---|---|
| Named consultant-led general antenatal clinic (maternal medicine clinic) | 63 (7) | 64.9 (7.2) |
| Consultant-led specialist hypertension in pregnancy clinic | 25 | 25.8 |
| Multi-disciplinary clinic with additional medical professional | 20 | 20.6 |
| Consultant obstetrician and midwife antenatal clinic | 15 | 15.5 |
| Shared-care GP/ obstetrician/ midwife | 7 | 7.2 |
| Specialist midwifery care (e.g. medical conditions team) | 6 | 6.2 |
| Hospital midwifery care | 1 | 1.0 |
| Community based midwifery care | 4 | 4.1 |
| Day assessment unit | 2 | 2.1 |

For peer review only

Question 13: In your maternity unit when do the pregnant women with chronic hypertension usually first get seen by an obstetrician?

| Gestation | Number (97) | Percentage % |
|---|---|---|
| Before 12 weeks | 24 | 24.7 |
| 12-15+6 weeks | 63 | 64.9 |
| 16-27+6 weeks | 9 | 9.3 |
| Missing data | 1 | 1.0 |

Question 14: Do you or someone in your unit specifically collect and analyse the outcomes of women with chronic hypertension in pregnancy annually?

| Response | Number (97) | Percentage (%) |
|---|---|---|
| Yes | 24 | 24.7 |
| No | 67 | 69.0 |
| Unsure | 4 | 4.1 |
| Some aspects | 2 | 2.0 |

For peer review only

Supplementary file 2

**Interview topic guide for clinicians:**

- Descriptions of the general approach to practice and how clinicians approach treatment decisions
- Discussion about the sources of evidence and knowledge that influence practice in general
- Participants' beliefs and experiences of using or having contact with clinical guidance (NICE in particular),
- Participants' views regarding how EBM and clinical guidelines could be better mobilised into practice

Interview schedule:

- Introductions
- Confidentiality
- I am interviewing you today for the CHAMPION study about chronic hypertension, you provide antenatal care for women with CHP is that right?
- Can you tell me a about your CHP clinic and your clinical practice in relation to chronic hypertension in pregnancy?
- How do you approach decision-making, for example commencing or changing hypertensive medication or delivery the baby early?
- What are you views and experiences of involving women in decision about their care or treatment plan?
- How do you source evidence and develop knowledge around hypertension in pregnancy?
- What are you experiences of clinical guidance e.g. NICE/ RCOG?
- How do you think we could better implement evidence-based medicine into clinical practice?

Reference: Grove, A., Clarke, A. and Currie, G. (2015) 'The barriers and facilitators to the implementation of clinical guidance in elective orthopaedic surgery: a qualitative study protocol', Implementation Science, 10(1), 81.

For peer review only

**Women's experience of their care**

- Introductions
- Confidentiality
- During this pregnancy you have been treated for chronic hypertension is that right?
- Can you tell me a bit about your high blood pressure and your pregnancy?
- How satisfied are you with the results of your care during your pregnancy?
- Thinking about your care during your pregnancy…Were you given information about your choices for maternity care?
- Were you given enough information to help you decide about your care?
- Were you given information at the right time to help you decide about your care?
- Did you have confidence and trust in the staff caring for you?

Reference: International Consortium for Health Outcomes Measurement. Pregnancy and Childbirth Standard Set and Reference Guide. 2016. http://www.ichom.org/medical-conditions/pregnancy-and-childbirth/.

Supplementary file 3

Maternal demographics of women observed, interviewed and included for case-note review. Women interviewed are a subset of those observed. Case-notes identified for review are a different cohort of women.

| Women demographics | Observed n=28 (%) | Interviewed n=18 (%) | Case-notes n=55 (%) |
|---|---|---|---|
| **Ethnicity** | | | |
| White British | 9 (32.0) | 7 (39.0) | 15 (27.3) |
| White Other | 6 (21.0) | 4 (22.0) | 8 (14.5) |
| Black | 9 (32.0) | 5 (28.0) | 18 (32.7) |
| Asian | 2 (7.0) | 1 (5.5) | 8 (14.5) |
| Any other | 2 (7.0) | 1 (5.5) | 6 (10.9) |
| **Parity at booking** | | | |
| 0 | 9 (32.0) | 7 (39.0) | 15 (27.3) |
| 1 | 11(39.0) | 7 (39.0) | 21 (38.2) |
| 2 | 7 (25.0) | 4 (22.0) | 10 (18.2) |
| 3 | 0 (0.0) | 0 (0.0) | 6 (10.9) |
| 4 | 0 (0.0) | 0 (0.0) | 2 (3.6) |
| 5 | 1 (4.0) | 0 (0.0) | 1 (1.8) |
| **Age** | | | |
| 20-34 | 17 (61.0) | 11 (61.0) | 23 (41.8) |
| 35-39 | 7 (25.0) | 5 (28.0) | 21 (38.9) |
| 40-44 | 4 (14.0) | 2 (11.0) | 11 (20.4) |
| **BMI** | | | |
| <18.5 | 0 (0.0) | 0 (0.0) | 1/52 (1.9) |
| 18.5-24.9 | 7 (25) | 6 (33.3) | 13/52 (25.0) |
| 25-29.9 | 10 (36) | 6 (33.3) | 13/52 (25.0) |
| 30-34.9 | 9 (32) | 5 (28.0) | 11/52 (21.2) |
| 35-39.0 | 2 (7) | 1 (5.5) | 6/52 (11.5) |
| >40.0 | 0 (0) | 0 (0.0) | 8/52 (7.7) |

For peer review only

Supplementary file 4

Pregnancy and birth outcomes – Case notes review

| **Outcomes** | **Case notes review**<br>**Nominator/denominator (%)** |
|---|---|
| Women with episode of severe hypertension | 25/55 (45.5) |
| 1st trimester episode | 2/40 (5.0) |
| 2nd trimester episode | 13/40 (32.5) |
| 3rd trimester episode | 25/40 (62.5) |
| Birth weight - median<br>(IQR1 – IQR3) | 2927.5<br>(2592.5 - 3200) |
| Admission to NNU | 9/55 (16.4) |

For peer review only

Supplementary file 5

Target blood pressure setting and prescribing practices per Trust – as derived from case-note review

| | **Hospital Trust 1 n=29 (%)** | **Hospital Trust 2 n=13 (%)** | **Hospital Trust 3 n=13 (%)** |
|---|---|---|---|
| **Target BP documented <150/100mmHg** | 20/26 (77.0) | 3/13 (23.0) | 5 (38.0) |
| **Labetalol** | 12/26 (46.0) | 7/12 (58.3) | 9/11 (82.0) |
| **Nifedipine** | 9/26 (34.5) | 0/12 (0.0) | 0/11 (0.0) |
| **Methyldopa** | 3/26 (11.5) | 4/12 (33.3) | 1/11 (9.0) |
| **Other** | 2/26 (8.0) | 1/12 (8.3) | 1/11 (9.0) |

For peer review only

Original research

# The quality of mixed methods studies in health services research

**Alicia O'Cathain, Elizabeth Murphy[1], Jon Nicholl**

Medical Care Research Unit, School of Health and Related Research, University of Sheffield, Sheffield; [1]School of Sociology and Social Policy, University of Nottingham, Nottingham, UK

***Objectives*: To assess the quality of mixed methods studies in health services research (HSR).**

***Methods*: We identified 118 mixed methods studies funded by the Department of Health in England between 1994 and 2004, and obtained proposals and/or final reports for 75. We applied a set of quality questions to both the proposal and report of each study, addressing the success of the study, the mixed methods design, the individual qualitative and quantitative components, the integration between methods and the inferences drawn from completed studies.**

***Results*: Most studies were completed successfully. Researchers mainly ignored the mixed methods design and described only the separate components of a study. There was a lack of justification for, and transparency of, the mixed methods design in both proposals and reports, and this had implications for making judgements about the quality of individual components in the context of the design used. There was also a lack of transparency of the individual methods in terms of clear exposition of data collection and analysis, and this was more a problem for the qualitative than the quantitative component: 42% (19/45) versus 18% (8/45) of proposals ($p = 0.011$). Judgements about integration could rarely be made due to the absence of an attempt at integration of data and findings from different components within a study.**

***Conclusions*: The HSR community could improve mixed methods studies by giving more consideration to describing and justifying the design, being transparent about the qualitative component, and attempting to integrate data and findings from the individual components.**

*Journal of Health Services Research & Policy* Vol 13 No 2, 2008: 92–98 © The Royal Society of Medicine Press Ltd 2008

## Introduction

Mixed methods studies are common in health services research (HSR).[1] They consist of two separate components of data collection and analysis within a single study: at least one quantitative method with structured data collection and statistical analysis, and at least one qualitative method with less structured data collection and thematic analysis.[2] Commissioners and consumers of research, as well as researchers themselves, need to judge whether a mixed methods study has been undertaken well or poorly, assessing whether it is good mixed methods research as well as good research. The quality of mixed methods research has been considered explicitly in health, educational and social research,[3–8] and implicitly when researchers have discussed the challenges of designing and implementing these studies.[9,10] However, the issue has received little consideration overall, with a recent search for quality criteria for mixed methods research concluding that there were none available,[7] even though attempts have been made to develop them.[3] Given that there are no agreed criteria for assessing the quality of these studies,[8] and that researchers are still debating the meaning of quality for mixed methods research,[6] it is premature to attempt to develop definitive criteria. Instead, it seems sensible to follow an approach taken by researchers considering quality in the context of synthesizing qualitative and quantitative evidence[11] and devise a set of questions which could be applied to mixed methods primary research to facilitate judgements about quality. We devised a set of 'quality questions' and applied them to proposals and reports of mixed methods studies to assess the quality of mixed methods studies in HSR.

**Alicia O'Cathain PhD**, MRC Fellow, **Jon Nicholl MSc**, Professor, Medical Care Research Unit, School of Health and Related Research, University of Sheffield, Regent Street, Sheffield S1 4DA, UK; **Elizabeth Murphy PhD**, Professor, School of Sociology and Social Policy, University of Nottingham, Nottingham, UK.

Correspondence to: a.ocathain@sheffield.ac.uk

## Methods

This research was part of a wider study exploring the use of mixed methods research in HSR. The wider study consisted of a quantitative documentary analysis of 75 mixed methods studies to determine the type

 DOI: 10.1258/jhsrp.2007.007074

For peer review only - http://bmjopen.bmj.com/site/about/guidelines.xhtml

and quality of mixed methods research undertaken, and qualitative interviews with 20 researchers to explore facilitators and barriers to exploiting the potential of this approach.[1,12]

## Devising questions about quality

We devised a framework for the quality assessment based on detailed consideration of the literature on mixed methods research in the fields of health, social and educational research. We searched the health databases MEDLINE and CINAHL. We then sought expert opinion encapsulated in key textbooks.[10,13–20] Finally we searched the Social Science Citations Index, PsycINFO, ERIC and the British Education Index to identify social, behavioural and educational research. The search for literature took place in 2003 and was updated in 2006. Quality was one of 11 issues identified in this review.

Within the literature, one suggested assessment criterion for mixed methods studies was whether they had been completed successfully in terms of adequately addressing the research questions with allocated resources.[5] Other researchers focused on the quality of methods. There was no suggestion of using a tool developed for generic use across all designs. Rather, researchers attempted to develop quality criteria by devising separate lists of criteria for the quantitative and the qualitative research.[7] Their assumption was that methods are linked to paradigms and therefore the criteria used to assess different methods should also be linked to paradigms.[7] However, not everyone agrees that methods are paradigm-specific[18] or that different criteria are needed for qualitative and quantitative research.[21] The same criteria have been proposed for both[21] although the appropriate means for judging against these criteria may differ because of the research practices employed in different methodological approaches. The mixed methods design[10] and the integration between methods[3] can be assessed as well as the individual methods. A good mixed methods study clearly justifies why a mixed methods approach is necessary or superior to another, offers transparency of the mixed methods design, and offers appropriate sampling, data collection and analysis of individual components relating to that design.[3,4,10] Thus the design may determine the criteria used to make judgements about the individual components of the study. Integration of data or findings from each component is a key part of mixed methods research,[10] distinguishing it from qualitative and quantitative studies undertaken independently. When integration occurs, it is important that data transformations are defensible, that contradictory findings are explained and convergent findings are not related to shared bias between methods.[3] Expertise may be needed within a research team to integrate at the analysis stage.[22] Finally, researchers have discussed the importance of inferences from mixed methods studies being trustworthy[6] and appropriate in the light of the design used.[3] As yet there are no criteria for assessing the quality of inferences from mixed methods research, although researchers are considering the complexity of this issue.[23]

When developing the framework for our quality questions we chose not to use a generic tool because they have variable applicability across different research designs.[24] We chose to assess the qualitative and quantitative components separately because they each contribute to the study as a whole and because the quality of one or both components may suffer as a consequence of being part of a mixed methods study.[25–27] In addition to the individual components, we included an assessment of the success of the study, the design, the integration and the inferences. Within this framework we constructed questions based on the literature review and reading the proposals and reports from four mixed methods studies in HSR.

## Identifying mixed methods studies

In 2004, mixed methods studies were identified through a systematic search of summaries of studies funded by the Department of Health, a key commissioner of health services research in England at that time. The methods have been described elsewhere[1,12] and are summarized here. Summaries of single studies funded between 1994 and 2004 through 10 programmes were read. The programmes were: Health Technology Assessment; Service Delivery and Organization; New and Emerging Applications of Technology; Policy Research Programme; and the NHS Research & Development programmes of maternal and child health, primary and secondary care interface, cardiovascular disease and stroke, forensic mental health, primary dental care, and promoting implementation of research findings. A total of 118 mixed methods studies were identified. The lead researcher of each study was written to with a request for the research proposal, the final report for completed studies and a list of any emerging publications.

## Application of quality questions

A data extraction form was devised which consisted of the quality questions with the tick box options of 'yes', 'yes, but improvements are possible', 'no', 'not enough information (NEI)' and 'not applicable (N/A)'. Space for open comments was available alongside each question, where the assessor (AOC) could record details of good and poor practice. The data extraction form was applied to each study by one researcher, first to the proposal and then to the report. Finally, any differences between the proposal and report were noted.

## Analysis

The structured data were entered into SPSS. The main analysis was descriptive, displaying the proportions of proposals and reports falling into each category of each question. The chi-squared test was used when

For peer review only

comparing results for the individual qualitative and quantitative components. Open comments were quantitized[28] by transcribing them into Word, grouping them into themes, and counting the number of studies in which a theme occurred.[29]

## Results

Documentation was received for 75 mixed methods studies. Full proposals were obtained for 60% (45/75) of the studies. Final reports were only available for the 52 studies completed by the time of data collection, and were obtained for 92% (48), although one was a summary report that was too brief for inclusion in the assessment of quality, leaving 47 reports. Both a proposal and report was available for 20 studies.

### Success

The potential to produce a successfully completed study was assessed using the research proposals. In most proposals, the quantitative methods appeared to be feasible within the time and money allocated (Table 1). However, even recognizing that some aspects of qualitative research cannot be fixed at the design stage (e.g. sample size for theoretical sampling), there was not enough detail to determine the feasibility of the qualitative methods in one-third of studies – for example, no indication of numbers of interviews to be undertaken or no indication of when the qualitative research would be conducted in the study timetable. We had concerns about the feasibility of the qualitative component in another one-third of proposals. From the open comments we identified 14 proposals where a large number of qualitative interviews were planned in a short time scale – for example, 40 interviews in four months without specifying the depth of interview and analysis. In nine of these studies the report was available and in four cases considerably fewer interviews were undertaken than planned. However, concerns highlighted about the feasibility of the qualitative research did not necessarily translate into shortfalls in the final study.

We defined a successful study as one that produced everything that had been planned at the proposal stage. A direct comparison of the final study report with the proposal was only possible on the subset of 20 studies for which both were available. In other cases the assessment relied on researchers detailing the planned and implemented study within their final report. Non-completion of a whole component of a study was rare (Table 1). However, in one-fifth of reports, one of the methods within a component was not executed as planned. This tended to be due to a range of problems in the field.

### Mixed methods design

A justification for using mixed methods research was only given in one-third of proposals and reports (Table 2). A minority of studies explicitly articulated the design in terms of the priority of methods, the purpose of combining methods, the sequence of methods and the stage at which integration would or did occur. It was particularly helpful for the subsequent quality assessment of individual components if researchers were explicit about the priority of methods and the role of any less dominant method. For example, it seemed inappropriate to have 40 in-depth interviews as a preliminary aid to develop a questionnaire, but appropriate if these interviews were also to be used as a primary means of investigating the issue under study. A lack of transparency of the overall design could occur in the context of excellent description of individual components.

When the design was not discussed explicitly it was usually possible to work out the key elements from reading the documentation. In most cases the design was assessed as appropriate for addressing the research question. However, researchers rarely discussed issues of rigour in relation to the design employed. An example of addressing rigour for the design was where researchers proposed that qualitative findings would not be shared with quantitative colleagues undertaking a randomized controlled trial to minimize the possibility of contamination of that trial; in another two studies, the qualitative research was undertaken with people not participating in the trial in order to avoid contaminating the trial. While the extent to which this attention to contamination avoidance was necessary may be debatable, it constitutes some evidence that researchers had given serious consideration to design issues related to mixed methods research.

For peer review only

**Table 1** Assessment of the success of mixed methods studies in HSR

| | Proposal (*n* = 45) | | | | Report (*n* = 47) | | | |
|---|---|---|---|---|---|---|---|---|
| | Yes | Yes, but | No | NEI or N/A | Yes | Yes, but | No | NEI or N/A |
| 1 Is the quantitative component feasible? | 82% | 2% | 4% | 11% | | | | |
| 2 Is the qualitative component feasible? | 38% | 20% | 13% | 29% | | | | |
| 3 Is the mixed methods design feasible? | 51% | 0% | 7% | 42% | | | | |
| 4 Have both qualitative and quantitative components been completed? | | | | | 87% | 6% | 2% | 4% |
| 5 Were some quantitative methods planned but not executed? | | | | | 19% | 0% | 45% | 36% |
| 6 Were some qualitative methods planned but not executed? | | | | | 21% | 2% | 38% | 38% |
| 7 Did the mixed methods design work in practice? | | | | | 85% | 0% | 2% | 13% |

NEI, not enough information; N/A, not applicable


For peer review only - http://bmjopen.bmj.com/site/about/guidelines.xhtml

**Table 2** Assessment of the mixed methods design of studies in HSR

| | Proposal (*n* = 45) | | | | Report (*n* = 47) | | | |
|---|---|---|---|---|---|---|---|---|
| | **Yes** | **Yes, but** | **No** | **NEI or N/A** | **Yes** | **Yes, but** | **No** | **NEI or N/A** |
| 1 Is the use of mixed methods research justified? | 31% | 3% | 60% | 4% | 30% | 2% | 66% | 2% |
| 2 Is the design for mixing methods described? | | | | | | | | |
| Priority | 16% | 2% | 78% | 4% | 15% | 0 | 83% | 2% |
| Purpose | 42% | 0 | 53% | 4% | 34% | 4% | 60% | 2% |
| Sequence | 56% | 0 | 40% | 4% | 49% | 0 | 49% | 2% |
| Stage of integration | 24% | 0 | 71% | 4% | 21% | 0 | 77% | 2% |
| 3 Is the design clearly communicated? | 80% | 0 | 16% | 4% | 81% | 4% | 9% | 6% |
| 4 Is the design appropriate for addressing the research questions? | 87% | 2% | 2% | 9% | 87% | 0% | 2% | 11% |
| 5 Has rigour of the design been considered (proposal) or adhered to (report)? | 7% | 0 | 93% | 0% | 21% | 0% | 0% | 79% |

NEI, not enough information; N/A, not applicable

## Quantitative component

The roles of the quantitative methods were usually communicated well within proposals and reports (Table 3). However, sufficient details were sometimes not given about these methods. In eight proposals the quantitative methods were only sketchily described and in a further 13 proposals some aspects of the quantitative methods were not described, in particular, the analysis (8) and the numbers involved (5). This was less of an issue for reports but nonetheless there were still problems with sketchy description overall (4) or little or no description of the analysis (5). This lack of transparency made it difficult to assess other aspects of quality.

Validity of the methods within the quantitative components was assessed by considering the attention researchers gave to issues such as confounding and bias. Validity was explicitly discussed in two-thirds of proposals, with little evidence that the rigour of any method was compromised (Table 3). There were few examples of an individual method being compromised by the mixed methods approach. One example was a Delphi exercise which was restricted in order to fit the timetable of the qualitative fieldwork.

It was difficult to determine the sophistication of proposed analyses due to the lack of detail about analysis in the research proposals. There was more information about analyses available in research reports and here concerns were identified about the sophistication of one-quarter of quantitative analyses. We identified 12 studies where the reported quantitative results seemed simplistic, sometimes only presenting descriptive statistics with no statistical tests and in two cases using an experimental design which was then ignored in the analysis.

## Qualitative component

The roles of the qualitative methods were usually communicated well within proposals and reports (Table 4). However, qualitative methods were often not described in sufficient detail and this occurred more frequently than for the quantitative components, both within proposals ($p = 0.011$) and reports ($p = 0.08$). First, there was sketchy description of the qualitative methods overall (15 proposals and 11 reports). In three of these reports there was no description of the qualitative methods at all, only the findings. Second, there were no details about an important aspect of the qualitative research, particularly the analysis (six proposals and nine reports). Third, one method was described in detail, usually interviews with a particular group, but a further qualitative method such as observation or focus groups appeared to be 'tagged on' with no description (six proposals). Fourth, the overall size of the qualitative component was not clear, with a few

**Table 3** Assessment of the quantitative component of mixed methods studies in HSR

| | Proposal (*n* = 45) | | | | Report (*n* = 47) | | | |
|---|---|---|---|---|---|---|---|---|
| | **Yes** | **Yes, but** | **No** | **NEI or N/A** | **Yes** | **Yes, but** | **No** | **NEI or N/A** |
| 1 Is the role of each method clear? | 98% | 0% | 2% | 0% | 96% | 2% | 0% | 2% |
| 2 Is each method described in sufficient detail? | 53% | 29% | 18% | 0% | 68% | 13% | 15% | 4% |
| 3 Is each method appropriate for addressing the research question? | 93% | 0 | 2% | 4% | 98% | 0% | 0% | 2% |
| 4 Is the approach to sampling and analysis appropriate for its purpose? | 67% | 4% | 4% | 24% | 70% | 9% | 6% | 15% |
| 5 Is there expertise among applicants/authors? | 67% | 2% | 7% | 24% | 30% | 0% | 0% | 70% |
| 6 Is there expertise on the team to undertake each method? | 60% | 0% | 2% | 24% | | | | |
| 7 Have issues of validity been addressed for each method? | 64% | 0% | 30% | 7% | 49% | 4% | 40% | 6% |
| 8 Has the rigour of any method been compromised? | 7% | 0% | 91% | 2% | 9% | 4% | 83% | 4% |
| 9 Is each method sufficiently developed for its purpose? | 84% | 0% | 7% | 9% | 83% | 0% | 4% | 13% |
| 10 Is the (intended) analysis sufficiently sophisticated? | 56% | 4% | 2% | 38% | 51% | 15% | 25% | 9% |

NEI, not enough information; N/A, not applicable

For peer review only - http://bmjopen.bmj.com/site/about/guidelines.xhtml

**Table 4** Assessment of the qualitative component of mixed methods studies in HSR

| | Proposal (*n* = 45) | | | | Report (*n* = 47) | | | |
|---|---|---|---|---|---|---|---|---|
| | Yes | Yes, but | No | NEI or N/A | Yes | Yes, but | No | NEI or N/A |
| 1 Is the role of each method clear? | 87% | 0% | 9% | 4% | 92% | 4% | 4% | 0% |
| 2 Is each method described in sufficient detail? | 24% | 29% | 42% | 4% | 38% | 28% | 30% | 4% |
| 3 Is each method appropriate for addressing the research question? | 87% | 7% | 2% | 4% | 91% | 2% | 2% | 4% |
| 4 Is the approach to sampling and analysis appropriate for its purpose? | 42% | 4% | 9% | 40% | 53% | 9% | 4% | 34% |
| 5 Is there expertise among the applicants/authors? | 56% | 2% | 11% | 31% | 32% | 4% | 0% | 64% |
| 6 Is there expertise on the team to undertake each method? | 44% | 9% | 7% | 40% | | | | |
| 7 Have issues of validity been addressed for each method? | 24% | 0% | 64% | 11% | 30% | 2% | 57% | 11% |
| 8 Has the rigour of any method been compromised? | 2% | 0% | 91% | 7% | 6% | 2% | 81% | 11% |
| 9 Is each method sufficiently developed for its purpose? | 64% | 0% | 9% | 27% | 77% | 2% | 9% | 13% |
| 10 Is the (intended) analysis sufficiently sophisticated? | 40% | 4% | 7% | 49% | 51% | 13% | 19% | 17% |

NEI, not enough information; N/A, not applicable

For peer review only

interviews here and there throughout the study adding up to a sizeable qualitative component of over 100 interviews (10 proposals).

Validity of the methods within the qualitative components was assessed by considering the attention researchers gave to issues such as reflexivity and negative cases. Validity was not addressed within proposals for more qualitative than quantitative components ($p = 0.001$), although any apparent difference in reports was not statistically significantly different ($p = 0.100$) (Table 4). Researchers did take the validity of qualitative methods seriously in some proposals, for example, paying attention to deviant cases and peer review of transcripts.

Concerns were identified with the sophistication of one-fifth of qualitative analyses. In nine studies the reported qualitative findings remained at a descriptive level, or reported findings in a quantitative manner only, or failed to distinguish between data collected using different methods such as focus groups and interviews.

## Integration

Integration of data or findings from the different methods received little attention in either proposals or reports, with researchers rarely discussing the type of integration, how it occurred in the context of team working and who was involved in it (Table 5). Because of the lack of integration, questions about the appropriateness of integration and the effect of integration on the rigour of individual methods were irrelevant.

## Inferences

In the reports, researchers were clear about which results had emerged from which methods, and inferences seemed appropriate (Table 6). For one-fifth of studies there was a concern that the inferences were based disproportionately on one method rather than the findings of all the methods. The imbalance was as likely to be towards qualitative findings as it was towards quantitative findings.

# Discussion

## The quality of studies in HSR

Mixed methods studies tend to be successful in HSR insofar that the qualitative and quantitative components are usually completed as planned. The main quality issue identified was a lack of transparency of the

**Table 5** Assessment of integration in mixed methods studies in HSR

| | Proposal (*n* = 45) | | | | Report (*n* = 47) | | | |
|---|---|---|---|---|---|---|---|---|
| | Yes | Yes, but | No | NEI or N/A | Yes | Yes, but | No | NEI or N/A |
| 1 Is the type of integration stated? | 11% | 0% | 84% | 4% | 2% | 2% | 94% | 2% |
| 2 Is the type of integration appropriate to the design? | 16% | 0% | 0% | 84% | 34% | 0% | 2% | 64% |
| 3 Has enough time been allocated for integration? | 2% | 0% | 13% | 85% | | | | |
| 4 Is the approach to integration detailed in terms of working together as a team? | 7% | 0% | 80% | 13% | | | | |
| 5 Does the dissemination strategy detail how the mixed methods will be reported in final reports and peer-reviewed publications? | 0% | 0% | 84% | 16% | | | | |
| 6 Are the personnel who participate in the integration clearly identified? | 9% | 0% | 80% | 11% | 6% | 0% | 70% | 23% |
| 7 Did appropriate members of the team participate in integration? | | | | | 0% | 0% | 2% | 98% |
| 8 Is there evidence of communication within the team? | | | | | 19% | 0% | 6% | 75% |
| 9 Has rigour been compromised by the process of integration? | | | | | 4% | 0% | 0% | 96% |

NEI, not enough information; N/A, not applicable


For peer review only - http://bmjopen.bmj.com/site/about/guidelines.xhtml

**Table 6** Assessment of the inferences made in completed reports of mixed methods studies in HSR

| | Report (*n* = 47) | | | |
|---|---|---|---|---|
| | Yes | Yes, but | No | NEI or N/A |
| 1 Is there clarity about which results have emerged from which methods? | 87% | 2% | 6% | 4% |
| 2 Are inferences appropriate? | 83% | 4% | 9% | 4% |
| 3 Are the results of all the methods considered sufficiently in the interpretation? | 66% | 6% | 19% | 9% |

NEI, not enough information; N/A, not applicable

mixed methods aspects of the studies and the individual components. The qualitative components were more likely to be poorly described than the quantitative ones. To some extent the poor description of qualitative methods is not a surprising finding given the historical dominance of quantitative methods in HSR. However, it raises concerns that the HSR community may be failing on occasions to exploit the potential of qualitative methods within mixed methods studies. Where a qualitative component is in a supporting role to a more dominant method, and does not have stand-alone status in terms of independently addressing an aspect of the research question, then limited description is acceptable. However, because researchers were often not explicit about the status of methods within the study design, it was difficult to make judgements about the individual components in the context of the design used. Integration of data and findings is a key part of mixed methods research. There was no evidence that inappropriate integration was undertaken because there was a tendency for researchers to keep the qualitative and quantitative components separate rather than attempt to integrate data or findings in reports or publications.[12]

## Developing quality criteria for mixed methods studies in HSR

There was a lack of transparency in the reporting of mixed methods studies in HSR which made it difficult to assess other aspects of the quality of these studies. This has been identified as a problem facing the quality assessment of other types of studies[11] and has led to the development of guidelines for reporting studies. Creswell has suggested a list of issues to consider when designing a mixed methods study[10] and we have considered this in conjunction with the literature on the quality of mixed methods studies to suggest some guidelines for Good Reporting of A Mixed Methods Study (GRAMMS) (Box 1). We present this as guidance for researchers rather than as a formal checklist.

**Box 1** Good Reporting of A Mixed Methods Study (GRAMMS)

(1) Describe the justification for using a mixed methods approach to the research question
(2) Describe the design in terms of the purpose, priority and sequence of methods
(3) Describe each method in terms of sampling, data collection and analysis
(4) Describe where integration has occurred, how it has occurred and who has participated in it
(5) Describe any limitation of one method associated with the present of the other method
(6) Describe any insights gained from mixing or integrating methods

## Limitations

The study is based on mixed methods research funded by one commissioner in one country. The response rate to requests for documentation for mixed methods studies was good but non-responders may have been more likely to be problematic studies, biasing the findings towards higher quality studies. The questions were devised and applied by one researcher (AOC) in the context of team discussions which meant that the data extraction process was unchallenged by an external source. A coding protocol was devised to accompany the data extraction form to aid transparency and reduce intra-rater variability. However the studies could have been rated differently by another researcher. Finally, the studies included were funded between 1994 and 2004 and improvements may have occurred since then.

We have taken a technical stance in our discussions of quality in mixed methods research. However, the philosophical stance adopted by researchers may affect the quality criteria they use, and wish to see applied to their studies. Subtle realism[30] has been proposed as a philosophical position appropriate for qualitative and quantitative research in health technology assessment.[21] An implication of this stance is that researchers would need to consider whether reflexivity has been applied to the whole of a mixed methods study rather than simply the qualitative component.

## Conclusions

This is the first attempt to consider the quality of mixed methods studies in HSR. We are not offering this as a definitive approach to be used by others, but to start the debate about how to assess and improve quality. We recommend that if we use mixed methods studies in HSR then we need to be more transparent about the design and the individual components in the context of the design, and attempt to integrate data and findings from the qualitative and quantitative methods.

## Acknowledgements

Many thanks to the researchers who kindly sent copies of their study documents. The Medical Research Council funded the study through their Fellowship scheme.

For peer review only

## References

1 O'Cathain A, Murphy E, Nicholl J. Why, and how, mixed methods research is undertaken in health services research: a mixed methods study. *BMC Health Serv Res* 2007;**7**:85
2 Bryman A. Quantitative and qualitative research: further reflections on their integration. In: Brannen J, ed. *Mixing Methods: Qualitative and Quantitative Research*. Aldershot: Ashgate, 1992:57–78
3 Caracelli VJ, Riggin LJC. Mixed-method evaluation: developing quality criteria through concept mapping. *Eval Pract* 1994;**15**:139–52
4 Creswell JW, Fetters MD, Ivankova NV. Designing a mixed methods study in primary care. *Ann Fam Med* 2004;**2**:7–12
5 Datta L. A pragmatic basis for mixed-method designs. In: Greene JC, Caracelli VJ, eds. *Advances in Mixed-method Evaluation: The Challenges and Benefits of Integrating Diverse Paradigms*. San Francisco, CA: Jossey-Bass, 1997:33–46
6 Teddlie C, Tashakkori A. Major issues and controversies in the use of mixed methods in the social and behavioural sciences. In: Tashakkori A, Teddlie C, eds. *Handbook of Mixed Methods in Social and Behavioural Research*. London: Sage Publications, 2003:3–50
7 Sale JEM, Brazil K. A strategy to identify critical appraisal criteria for primary mixed method studies. *Quality and Quantity* 2004;**38**:351–65
8 Creswell JW, Plano-Clark V. *Designing and Conducting Mixed Methods Research*. Thousand Oaks, CA: Sage Publications, 2007
9 Brannen J. Combining qualitative and quantitative approaches: an overview. In: Brannen J, ed. *Mixing Methods: Qualitative and Quantitative Research*. Aldershot: Ashgate, 1992:3–38
10 Creswell JW. *Research Design. Qualitative, Quantitative, and Mixed Methods Approaches*. 2nd edn. London: Sage Publications, 2003
11 Mays N, Pope C, Popay J. Systematically reviewing qualitative and quantitative evidence to inform management and policy-making in the health field. *J Health Serv Res Policy* 2005;**10** (Suppl. 1):6–20
12 O'Cathain A, Murphy E, Nicholl J. Integration and publications as indicators of 'yield' from mixed methods studies. *Journal of Mixed Methods Research* 2007;**1**:147–63
13 Brewer J, Hunter A. *Multimethod Research*. London: Sage Publications, 1989
14 Tashakkori A, Teddlie C, eds. *Handbook of Mixed Methods in Social and Behavioural Research*. London: Sage, 2003
15 Tashakkori A, Teddlie C. *Mixed Methodology: Combining Qualitative and Quantitative Approaches*. London: Sage, 1998
16 Gorard S, Taylor C. *Combining Methods in Educational and Social Research*. Maidenhead: Open University Press, 2004
17 Brannen J, ed. *Mixing Methods: Qualitative and Quantitative Research*. Aldershot: Ashgate, 1992
18 Bryman A. *Quantity and Quality in Social Research*. London: Routledge, 1988
19 Greene JC, Caracelli VJ, eds. *Advances in Mixed-method Evaluation: The Challenges and Benefits of Integrating Diverse Paradigms*. San Francisco, CA: Jossey-Bass, 1997
20 Fielding NG, Fielding JL. *Linking Data*. Sage Publications, 1986
21 Murphy E, Dingwall R, Greatbatch D, Parker S, Watson P. Qualitative research methods in health technology assessment: a review of the literature. *Health Tech Assess* 1998;**2**:16
22 Mason J. Linking qualitative and quantitative data analysis. In: Bryman A, Burgess RG, eds. *Analysing Qualitative Data*. London: Routledge, 1994:89–110
23 Miller S. Impact of mixed methods and design on inference quality. In: Tashakkori A, Teddlie C, eds. *Handbook of Mixed Methods in Social and Behavioural Research*. London: Sage Publications, 2003:423–55
24 Katrak P, Bialocerkowski AE, Massy-Westropp N, Kumar VS, Grimmer KA. A systematic review of the content of critical appraisal tools. *BMC Med Res Meth* 2004;**4**:22
25 Silverman D. *Doing Qualitative Research. A Practical Handbook*. London: Sage Publications, 2000
26 Steckler A, Mcleroy KR, Goodman RM, Bird ST, McCormick L. Toward integrating qualitative and quantitative methods: an introduction. *Health Educ Q* 1992;**19**:1–8
27 Chen H. Applying mixed methods: a dominant methodology for the future? In: Greene JC, Caracelli VJ, eds. *Advances in Mixed-method Evaluation: The Challenges and Benefits of Integrating Diverse Paradigms*. San Francisco, CA: Jossey-Bass, 1997:61–72
28 Sandelowski M. Combining qualitative and quantitative sampling, data collection, and analysis techniques in mixed-method studies. *Res Nurs Health* 2000;**23**:246–55
29 O'Cathain A, Thomas KJ. "Any other comments?" Open questions on questionnaires – a bane or a bonus to research? *BMC Med Res Meth* 2004;**4**:25
30 Hammersley M. *What's Wrong with Ethnography?* London: Routledge, 1992

For peer review only


For peer review only - http://bmjopen.bmj.com/site/about/guidelines.xhtml
